# Synthesis of Pertyolides A, B, and C: A Synthetic Procedure to C_17_-Sesquiterpenoids and a Study of Their Phytotoxic Activity

**DOI:** 10.1021/acs.jnatprod.1c00396

**Published:** 2021-08-09

**Authors:** David M. Cárdenas, Carlos Rial, Rosa M. Varela, José M. G. Molinillo, Francisco A. Macías

**Affiliations:** † Allelopathy Group, Department of Organic Chemistry, Institute of Biomolecules (INBIO), Campus CEIA3, School of Science, University of Cadiz, C/República Saharaui 7, 11510 Puerto Real, Cádiz, Spain

## Abstract

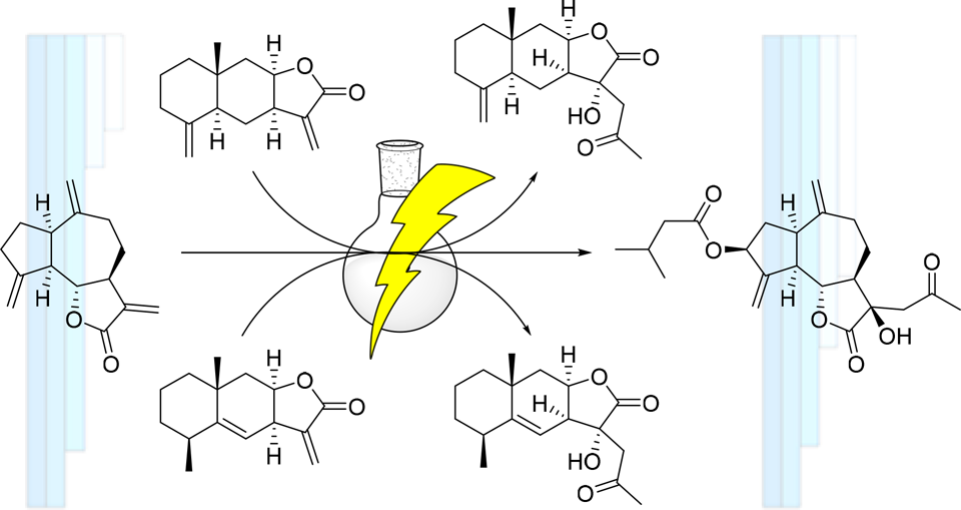

C_17_-sesquiterpenoids are a group of natural products that have been recently discovered. These compounds have the peculiarity of lacking the α,β-methylene butyrolactone system, which is known to be quite relevant for many of the biological activities reported for sesquiterpene lactones. Unfortunately, the biological interest of C_17_-sesquiterpenoids has not been studied in-depth, mainly due to the poor isolation yields in which they can be obtained from natural sources. Therefore, in order to allow a deeper study of these novel molecules, we have worked out a synthetic pathway that provides C_17_-sesquiterpenoids in enough quantities from easily accessible sesquiterpene lactones to enable a more thorough investigation of their bioactivities. With this synthesis method, we have successfully synthesized, for the first time, three natural C_17_-sesquiterpenoids, pertyolides A, B, and C, with good overall yields. Furthermore, we have also evaluated their phytotoxicity against etiolated wheat coleoptiles and corroborated that pertyolides B and C present strong phytotoxic activity.

Herbicide resistance is one of the major troublesome phenomena faced by modern agriculture and the agro-food industry. Herbicide resistance has been defined by the Herbicide Resistance Action Committee as “the inherited ability of a plant to survive and reproduce following exposure to a dose of herbicide that would normally be lethal to the wildtype”.^1^ The main reason herbicide resistance has become a huge threat to agriculture worldwide is the intensive and even abusive use of synthetic herbicides. Nowadays, more than 200 active ingredients have been registered as herbicides, but only 29 mechanisms of action have been described.^2^

In order to fight herbicide resistance, it is necessary to find new herbicides with mechanisms of action different from those exhibited by current herbicides. Natural products present themselves as an attractive alternative to synthetic herbicides, as they have been demonstrated to possess strong phytotoxic activities and they are frequently synthesized by certain organisms through complex pathways that lead to molecules that employ different mechanisms of action from those used by current herbicides.^3^

Sesquiterpene lactones (SLs) are one of the largest families of natural products. They can be mainly isolated from the aerial parts of certain plants from the Compositae family, although they can also be found in other plant families such as Umbelliferae, Lauraceae, or Magnoliaceae.^4^ The interest in the use of SLs for their allelopathic effects lies on the fact that they are produced by numerous weeds for the same purpose. They have been deeply studied in the past, and it has been demonstrated that they not only play a defensive role for the plants that generate them, but also possess a large range of biological activities of interest both in the fields of allelopathy^5−8^ and medicine.^9−13^

Many studies support that the α-methylene-butyrolactone system plays an essential role in the bioactivities of SLs.^14^ The mechanism of action of SLs is associated with their α-methylene-butyrolactone moiety, which acts as a strong and selective alkylating agent with nucleophile substrates, such as the sulfhydryl groups that are present in proteins, through Michael addition.^15^ However, the presence of such system may not be strictly necessary for the bioactivity of SLs. In fact, the most immediate and direct factor for the cytotoxic activity of SLs is the presence of α,β-unsaturated keto group not necessarily related to the carbonyl group in their lactone ring.^16^ In another study it was observed that the lack of said system would not significantly reduce the stimulation of the germination of parasitic plants.^17^

In recent studies, a natural group of sesquiterpene lactone derivatives, commonly referred to as C_17_-sesquiterpenoids, with an unusual carbon skeleton formed by 17 carbons, has been discovered.^18−21^ It has been observed that its α,β-unsaturated system conjugated to the lactone ring of C-_17_-sesquiterpenoids has been modified and that a C_2_ unit has been appended to it, generally, with polar functionalizations, such as a keto or hydroxy group. This fact may suggest that these molecules perform their bioactivity through a mechanism of action not related to the α,β-methylene-butyrolactone system or that the modified fragment improves the physicochemical properties of the molecule, like, for example, the aqueous solubility. SLs are known to exhibit poor aqueous solubility, which may hinder their bioactivity because of their inability to reach their site of action. The modifications that take place in their α,β-unsaturated system may just serve to optimize their physicochemical properties or their spatial arrangement, which would result in an increase of their activities.^22^

Even though C_17_-sesquiterpenoids have been successfully isolated from natural sources, not many studies have been found regarding their biological interest. Up to the present, only a handful of studies regarding their anti-inflammatory and antitumor activities have been conducted.^18−21^ No data regarding their phytotoxic activity or their mechanism of action have been reported, mainly due to the poor yields that have been obtained until now through isolation.

In order to further study the biological properties of C_17_-sesquiterpenoids, we have synthesized three C_17_-sesquiterpenoids and evaluated their phytotoxic activity. The synthesis of the C_17_-sesquiterpenoids comprises two key steps: a photochemical addition of acetaldehyde to the α,β-unsaturated double bond of the lactone ring in order to add the C_2_ chain and a hydroxylation in the α position of the lactone group. By applying this synthesizing procedure, we have succeeded to synthesize for the first time pertyolides A (**1**), B (**2**), and C (**3**) using isoalantolactone (**4**), alantolactone (**5**), and dehydrocostuslactone (**6**) as starting materials, respectively. We have also evaluated, for the first time, the phytotoxic activities of these molecules against etiolated wheat coleoptiles.
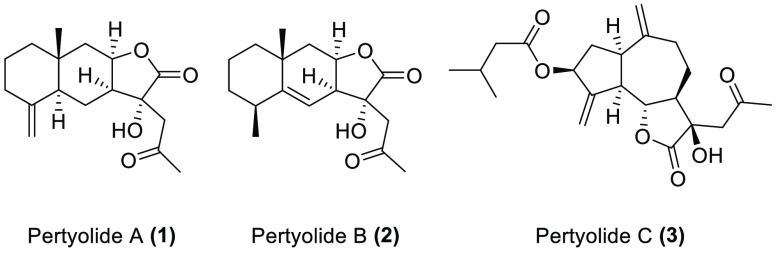


## Results and Discussion

Isoalantolactone (**4**) and alantolactone (**5**) were isolated by column chromatography from the methanolic extract of dried *Inula helenium* roots.^23^ From 160 g of methanolic extract, 1.5 g of **4** (0.9% yield) and 4.5 g of **5** (2.8% yield) were obtained as colorless crystalline solids. Compound **6** was obtained from a *Saussurea lappa* root extract following the procedure described in the literature.^24^ From 50 g of *S. lappa* root extract, 2.3 g of **6** (5.0% yield) were obtained as a colorless crystalline solid.

**Scheme 1:**
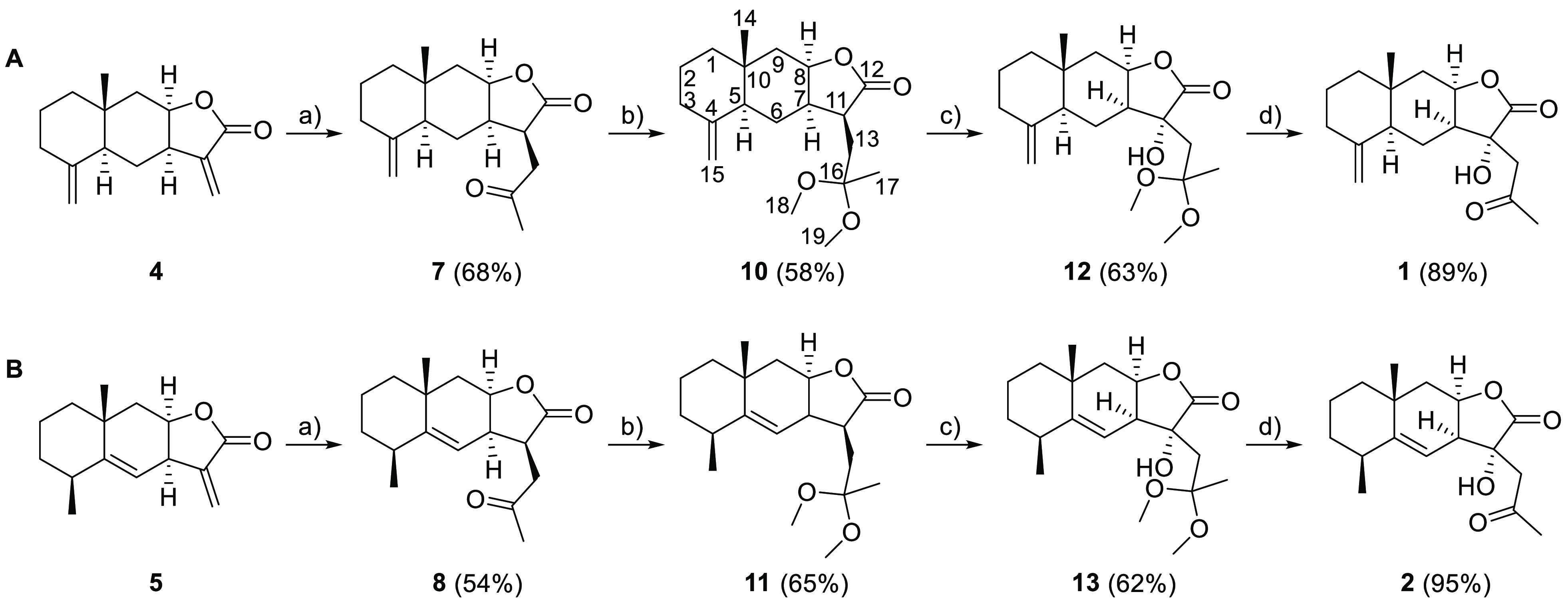
Synthesis of (A) Pertyolide A from Isoalantolactone and (B) Pertyolide B from Alantolactone CH_3_CHO, hυ. TMOF/MeOH, *p*-TsOH, rt. (1) KHDMS, THF, O_2_, −73 °C. (2) P(OCH_2_CH_3_)_3_, THF, −20 °C. Acetone/H_2_O 95:5, *p*-TsOH, rt.

**Scheme 2:**
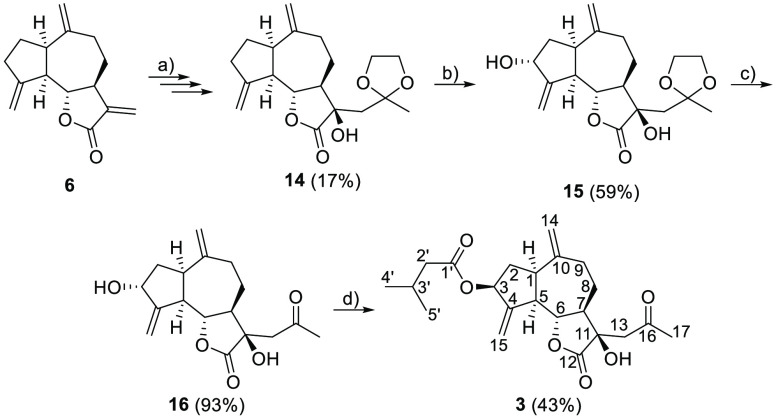
Synthesis of Pertyolide C from Dehydrocostus Lactone Previous work^24^ (3 steps). SeO_2_, TBHP. Acetone/H_2_O 95:5, *p*-TsOH, rt. DIAD, PPh_3_, (CH_3_)_2_CHCH_2_COOH.

After successfully isolating the starting materials, we applied the synthesizing routes shown in Schemes 1 and 2 to obtain pertyolides A, B, and C. Compounds **1** and **2** were synthesized in four steps. The photochemical addition of acetaldehyde to the starting sesquiterpene lactones yields the 1,4-dicarbonyl derivatives **7** (68% yield) and **8** (54% yield) with a C_17_ skeleton following the procedure described in the literature.^25−27^ (For NMR data, see Table S3, Supporting Information).

**Figure 1:**
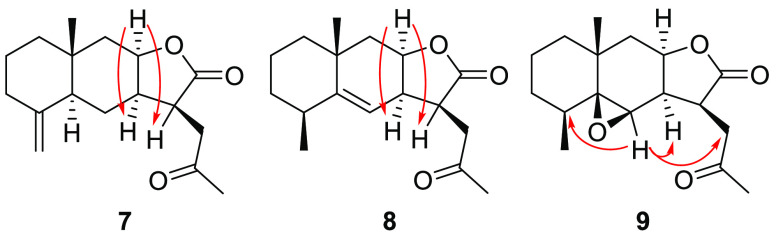
Positive NOE correlations observed for compounds **7**, **8**, and **9**.

When the photochemical addition was carried out with alantolactone, the epoxide byproduct **9** (Figure 1) was also generated along with the desired 1,4-dicarbonyl derivative **8**. It is unclear how the photoaddition would lead to the formation of the epoxide from the endocyclic double bond. The authors believe that two parallel reactions take place: the desired photochemical addition of acetaldehyde at position C-13 and also an undesired epoxidation which may be due to an interaction between the singlet oxygen generated and the already mentioned double bond. Different trials were conducted modifying the reaction time to optimize the yield of the desired product, and the best results were obtained with 2 h of reaction time. NOESY experiments were carried out to determine the relative configuration of the C-11 center of derivatives **7** and **8** (Figure 1), as well as the orientation of the epoxide group of **9**. The C_3_ chain was determined to be β-oriented due to the positive NOE effects between H-8 and H-7 and H-11. On the other hand, the epoxy group was determined to be β-oriented due to the positive NOE correlations between H-6 and H-4, H-7 and H-13. The absolute configuration of the molecules has been established from the known absolute configuration of the precursors.

After obtaining **7** and **8**, the new carbonyl group at position 16 must be masked to enable the later regioselective hydroxylation at position 11. The procedures described in our previous work were applied to mask the keto group.^24^ In the first instance, the keto group was treated with 2-ethyl-2-methyl-1,3-dioxolane (MED) as a protective agent, to obtain the corresponding cyclic ketal. However, after several attempts using different Lewis acids (*p*-TsOH, aluminum trichloride (AlCl_3_) and boron trifluoride-ethyl etherate (BF_3_·C_4_H_10_O), as well as different temperature levels and reaction times, the target cyclic ketal was never obtained. A number of trials were also conducted by applying a Dean–Stark trap procedure, where ethylene glycol was used as the protective agent, but in most cases the starting material remained unchanged or degradation products were observed.

In order to mask the carbonyl group and obtain the desired acyclic ketals (**10** and **11**), a different strategy was devised that consisted in the protection of the keto group by means of trimethyl orthoformate (TMOF) in the presence of anhydrous MeOH and catalytic amounts of *p*-TsOH.^28^ As a result, the acyclic ketal derivatives **10** and **11** were obtained at 58% and 65% yields, respectively (refer to Table S4 for NMR data).

Once the ketal derivatives (**10** and **11**) had been obtained, hydroxylation at the α position of the lactone group was carried out by peroxidation followed by a reduction step in one pot according to the procedure described in the literature.^29^ The treatment of the derivative **10** yielded a mixture of the desired hydroxylated ketal **12** (63%) and **1** (22%), while the derivative **11** yielded 62% of **13** and 18% of **2**. The attempts to purify the hydroxylated ketals **12** and **13** were conducted by means of column chromatography and HPLC. However, they could not be successfully separated and instead, 9:1 mixtures with their respective pertyolide as minor constituent was obtained.

It is well-known that ketals are labile in acidic media and can be easily deprotected through hydrolysis or transketalization. Some reports suggest that acyclic acetals can be alternatively deprotected in aqueous solution without the presence of an acid.^30^ Accordingly, during the workup of the reaction, the crude is treated with Sörensen buffer (an aqueous solution of Na_2_HPO_4_ and KH_2_PO_4_, pH 7.2). The authors believe that this step is responsible for the partial hydrolysis of the derivatives **12** and **13**.

The complete deprotection of the ketals **12** and **13** was performed by transketalization in an acetone:H_2_O 95:5 mixture with catalytic amounts of *p*-TsOH according to the procedure described in the literature.^27^ This procedure produced pertyolide A (**1**) and B (**2**) both as crystalline solids in 89% and 95% yields, respectively. The NMR and the spectroscopy data of the synthesized **1** and **2** match those reported in the bibliography^18^ (see Table S1 for more detailed NMR data and Tables S6 and S7 for a comparison with the NMR data reported for the isolated **1** and **2**).

Pertyolide C (**3**) was synthesized through six steps as shown in Scheme 2. According to the procedure described in our previous work,^24^**14** was synthesized from **6** in three steps. Then, an allylic oxidation step using selenium dioxide and *tert*-butyl hydroperoxide (TBHP)^31^ yielded the dihydroxylated ketal **15** in 59%. The compound **15** was then deprotected via transketalization under the same conditions used for pertyolides A and B, and a 93% yield of the compound **16** was obtained (see Table S5 for the NMR data of the compounds **15** and **16**).

The last step of the synthetic pathway consists of the esterification of the hydroxy group at C-3 using isovaleric acid with complete Walden inversion of the alcohol stereocenter. This can be done in one pot through the Mitsunobu reaction. The treatment of the compound **16** under the conditions described in the literature,^32^ i.e., with isovaleric acid, diisopropylazodicarboxylate (DIAD), and triphenylphosphine (PPh_3_), produced pertyolide C (**3**) as a colorless oil at 43% yield. The relative configuration of the C-3 center was determined by the positive NOE correlations between H-3 and H-1 and H-5, which proved that the isovalerate group introduced is β-oriented. The absolute configuration of the molecule has been established from the known absolute configuration of the precursors. The NMR data and the spectroscopy data of the synthesized pertyolide C match those reported in the bibliography^18^ (see Table S2 for more detailed NMR data and Table S8 for a comparison with the NMR data reported for isolated **3**).

The phytotoxic activities of the new compounds were evaluated against etiolated wheat coleoptiles following the procedure developed by our research group.^33^ The usage of etiolated wheat coleoptiles is a common practice in the field of allelopathy in order to evaluate phytotoxic activity. This is a rapid and sensitive procedure that can be applied to a wide range of bioactive substances, such as plant growth regulators, herbicides, or mycotoxins.^34,35^

**Figure 2:**
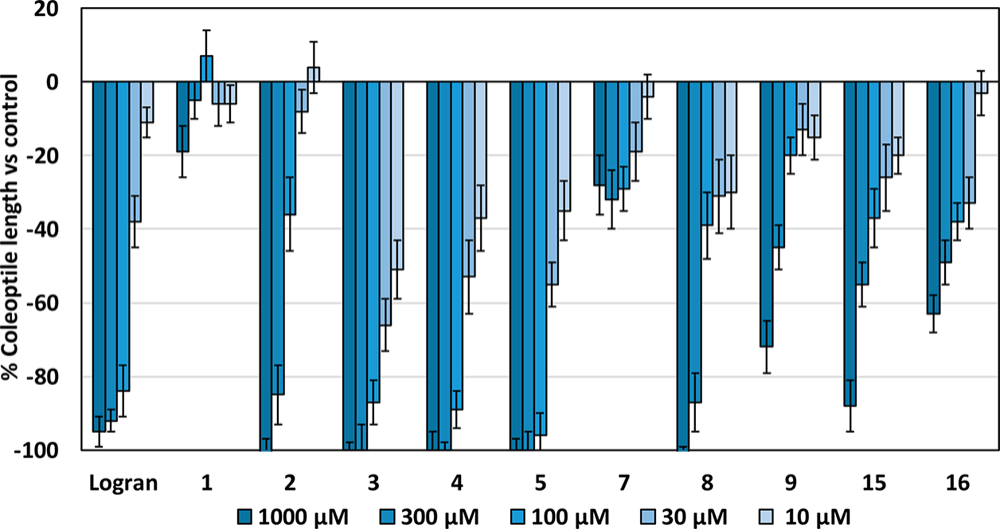
Bioassay data on the phytotoxicity profiles of the assayed compounds against etiolated wheat coleoptiles.

The recorded data on the activity profiles of each molecule (Figure 2) were fitted to a sigmoidal dose–response curve to determine their IC_50_ values (Table 1) and establish a comparison of the bioactivities of the synthesized molecules against that of the commercial herbicide Logran (active ingredients 59.4% terbutryn and 0.6% triasulfuron), which had been used as the positive control. The ketals **10**, **12**, **11**, and **13** were not evaluated due to their instability in aqueous media. All of the starting materials, dehydrocostuslactone (**6**), isoalantolactone (**4**), and alantolactone (**5**) exhibited strong inhibition levels (>90%), comparable to that of the commercial herbicide Logran, when used at concentrations above 100 μM, although with a significant drop in their activity at lower concentrations. In the case of the eudesmanolide C_17_-sesquiterpenoids synthesized, we could observe substantially different behaviors, where isoalantolactone derivatives, i.e., both the ketone derivative **7** and pertyolide A (**1**) did not show any relevant activity even at the highest concentrations tested. On the contrary, their isomers, i.e., compound **8** and pertyolide B (**2**), presented strong inhibitory activity at concentrations above 300 μM with a significant drop at lower concentrations.

**Table 1:** IC_50_ Values Calculated for the Molecules Tested against Etiolated Wheat Coleoptiles

	IC_50_ (μM)	RMSE^a^	*R*^2^
Logran^b^	38	6	0.9980
1^c^	–	–	–
2	130	10	0.9698
3	12	2	0.9903
4	22	4	0.9883
5	20	6	0.9828
7	–	–	–
8	120	13	0.9356
9	440	5	0.9878
15	230	8	0.9717
16	290	13	0.9091

a Root mean square error.

b Logran was used as positive control.

c No data is shown when 50% of inhibition is not achieved at the highest concentration tested (1000 μM).

The profiles of the compounds **2** and **8** reveal that even after eliminating the α-methylene-butyrolactone system, the molecules still have a strong inhibitory activity at concentrations above 300 μM with a drop of their activity below that concentration level. By contrast, a huge drop of the activity can be observed in compounds **1** and **7** compared to that of compound **4**. This may suggest that, in the specific case of the isoalantolactone skeleton, the α-methylene-butyrolactone moiety is important for the phytotoxic activity of isoalantolactone or that the modifications that take place in the C_2_ appended skeleton prevent the molecules from reaching their site of action. When establishing a correlation between a chemical structure and a bioactivity there are many properties, such as steric and electronic aspects, lipophilicity, or aqueous solubility, that play an essential role in the capability of the molecules to reach their site of action inside a living organism and exert their biological effects. It is possible that the structural modifications of isoalantolactone alter these properties, hindering the capability of the molecule to interact with its biological target.

In the case of the synthesized guaianolide C_17_-sesquiterpenoids, our previous work demonstrates that similarly to alantolactone, the elimination of the α-methylene-butyrolactone system only caused a slight drop in the phytotoxic activity of the molecules. Another slight drop of the activity by both compounds **15** and **16** was observed when the second hydroxy group was introduced at position C-3. However, pertyolide C (**3**) experienced a large improvement of its activity after the hydroxy group at position C-3 had been modified by adding the isovalerate group. In fact, pertyolide C (**3**) exhibited the strongest inhibitory profile of all the molecules and at every concentration level within the range tested (1000 μM to 10 μM) with an outstanding IC_50_ value of 12 μM, which is lower than that of dehydrocostuslactone (**6**) (170 μM)^24^ or the commercial herbicide Logran (38 μM).

According to the results obtained from the bioassays with etiolated wheat coleoptiles, some of the target C_17_-sesquiterpenoids and intermediates synthesized present similar or even superior activity to the original sesquiterpene lactone containing the α-methylene-butyrolactone system. No clear trend could be observed to support that the elimination of the said moiety has a negative impact on the phytotoxic activity of the molecules. This led us to think that the phytotoxic activity of C_17_-sesquiterpenoids may take place at a different site of action in the molecule and that they may have a specific mechanism of action other than the Michael addition reactions that are commonly associated with the activity of sesquiterpene lactones.

In conclusion, we have applied a previously designed general synthetic pathway that has allowed the synthesis, for the first time, of three natural C_17_-sesquiterpenoids, pertyolides A (**1**), B (**2**), and C (**3**) with 29%, 26%, and 4% overall yields through two key steps: the photochemical addition of acetaldehyde to the α-methylene-butyrolactone system of the sesquiterpene lactone and a hydroxylation at the α position of the lactone group. Furthermore, the phytotoxic activities of the target molecules, as well as their intermediates, were evaluated against etiolated wheat coleoptiles to determine their profile of activity and their IC_50_ values. Pertyolide C (**3**) presented the highest inhibitory activity with an outstanding IC_50_ value of 12 μM. The data obtained from the bioassays suggest that the α-methylene-butyrolactone moiety is not essential for these types of molecules to present phytotoxic properties.

## Experimental Section

### General Experimental Procedures

The melting points were determined by means of a Kofler hot bench. The optical rotations were measured on a Jasco P-2000 polarimeter using CHCl_3_ as solvent. The FTIR spectra were obtained using a PerkinElmer Spectrum TWO IR spectrophotometer. The major absorptions in the IR spectra are given as wavenumbers (υ̃) in cm^–1^. ^1^H NMR and ^13^C NMR spectra were recorded on Agilent spectrometers at 400/100 and 500/125 MHz using CDCl_3_ (Magnisolv, Merck) or C_6_D_6_ (Magnisolv, Merck) as internal reference. The solvents residual peaks were set to δ 7.26 ppm for ^1^H NMR and δ 77.0 ppm for ^13^C NMR in the case of CDCl_3_ and δ 7.16 ppm for ^1^H NMR and δ 128.1 ppm for ^13^C NMR in the case of C_6_D_6_. The exact masses were measured on a UPLC-QTOF-ESI (Waters Synapt G2) high-resolution mass spectrometer (HRTOFESIMS). The reactions were monitored by thin layer chromatography using Merck Kiesegel 60 F_254_ normal phase plates. The resulting products were purified by column chromatography using silica gel Geduran Si 60 (0.063–0.200 mm) or by HPLC using a Merck-Hitachi *D-2500* with refractive index detector and a Merck LiChrospher 60 (10 μm, 250 × 10 mm) column. The reagents for the synthetic procedures were supplied by either Sigma-Aldrich Co., Merck, Alfa Aesar, or Acros Organics. The solvents used for purification were supplied by VWR International. Logran Extra 60 WG (Syngenta Agro, S. A.) was used as positive control in the etiolated wheat coleoptile bioassay.

### Extraction and Isolation of the Starting Materials (**4**, **5**, and **6**)

Isoalantolactone (**4**) and alantolactone (**5**) were isolated from commercial *Inula helenium* roots purchased from “Centro dietético Víquez-Herbolario Cádiz” (Cádiz, Spain). Dry roots (1.0 kg) were extracted with MeOH for 3 days. The methanolic extract was filtered under vacuum and the solvent evaporated under reduced pressure. The extract obtained (160 g) was redissolved in MeOH and further purified by column chromatography using a hexane:EtOAc 95:5 mixture; 1.5 g of isoalantolactone (0.9% yield) and 4.5 g of alantolactone (2.8% yield) were obtained as colorless crystalline solids.

Dehydrocostuslactone (**6**) was isolated from a *Saussurea lappa* root oil extract purchased from Pierre Chauvet S.A. The extract, (50 g) was dissolved in CH_2_Cl_2_ and purified by column chromatography using a hexane:EtOAc 95:5 mixture; 2.3 g of dehydrocostuslactone (5.0% yield) were obtained as a colorless crystalline solid.

### Synthesis of the 1,4-Dicarbonyl Derivatives (**7** and **8**)

The sesquiterpene lactones **4** and **5** (100 mg each) were each dissolved in 100 mL of previously distilled acetaldehyde and introduced in a modified Hanovia reactor fitted with a Pyrex jacket. A 125 W medium pressure Hg lamp model Radium from Radium was set into the reactor at a distance of approximately 10.0 cm from the reaction mixture. An aqueous solution of NiSO_4_·6H_2_O (46 g) and CoSO_4_·7H_2_O (14g) in 100 mL of H_2_O was used as filter solution to restrict the wavelengths to a small window around 300 nm and avoid the formation of undesired byproducts. The reaction was stirred for 1 or 2 h (1 h for **4** and 2 h for **5**) at room temperature (rt) and the solvent was evaporated under reduced pressure. During this process small amounts of cyclohexane were added to eliminate the acetic acid generated as a reaction byproduct. The crude product was purified by column chromatography using a mixture of hexane:EtOAc 9:1 to 6:4 and either the corresponding 1,4-dicarbonyl derivative **7** at 68% yield (80.9 mg, 2.93 × 10^–1^ mmol) or a mixture of the compound **8** at 54% yield (64.2 mg, 2.32 × 10^–1^ mmol) as well as a 19% yield of the compound **9** (23.9 mg, 8.17 × 10^–2^ mmol) were obtained.

#### (3*S*,3a*R*,4a*S*,8a*R*,9a*R*)-8a-Methyl-5-methylene-3-(2-oxopropyl)decahydronaphtho[2,3-*b*]furan-2(3*H*)-one (**7**)

Crystalline solid; mp 131–134 °C; [α]_Na_^25^ +89.8 (*c* 0.230, CHCl_3_); IR (film) υ̃_max_ 1746, 1716 cm^–1^; ^1^H NMR (CDCl_3_, 400 MHz) δ 4.73 (1H, d, *J* = 1.2 Hz, H-15a), 4.51 (1H, ddd, *J* = 4.2, 4.2, 1.7 Hz, H-8), 4.39 (1H, d, *J* = 1.2 Hz, H-15b), 3.22 (1H, ddd, *J* = 9.3, 6.4, 4.5 Hz, H-11), 2.97 (1H, dd, *J* = 18.7, 4.5 Hz, H-13a), 2.63 (1H, dd, *J* = 18.7, 9.3 Hz, H-13b), 2.56 (1H, dddd, *J* = 12.3, 6.4, 6.2, 4.2 Hz, H-7), 2.28 (1H, m, H-3a), 2.20 (3H, s, H-17a, b, c), 2.12 (1H, dd, *J* = 15.5, 1.7 Hz, H-9a), 1.94 (1H, ddd, *J* = 18.6, 12.7, 5.8 Hz, H-3b), 1.74 (1H, brd, *J* = 12.5 Hz, H-5), 1.52 (2H, m, H-2a, b), 1.48 (1H, m, H-1a), 1.44 (1H, dd, *J* = 15.5, 4.2 Hz, H-9b), 1.33 (1H, ddd, *J* = 13.2, 6.2, 2.4 Hz, H-6a), 1.19 (1H, m, H1b), 1.03 (1H, ddd, *J* = 13.2, 12.5, 12.3 Hz, H-6b), 0.74 (3H, s, H-14a, b, c); ^13^C NMR (CDCl_3_,100 MHz) δ 205.9 (C, C-16), 178.0 (C, C-12), 149.2 (C, C-4), 106.2 (CH_2_, C-15), 78.3 (CH, C-8), 46.2 (CH, C-5), 42.6 (CH, C-11), 42.0 (CH_2_, C-1), 41.3 (CH_2_, C-9), 38.7 (CH, C-7), 38.4 (CH_2_, C-13), 36.6 (CH_2_, C-3), 34.7 (C, C-10), 30.0 (CH_3_, C-17), 22.5 (CH_2_, C-2), 21.3 (CH_2_, C-6), 17.6 (CH_3_, C-14); HRESIMS *m*/*z* 277.1810 [M + H]^+^ (calcd for C_17_H_25_O_3_, 277.1804).

#### (3*S*,3a*R*,5*S*,8a*R*,9a*R*)-5,8a-Dimethyl-3-(2-oxopropyl)-3a,5,6,7,8,8a,9,9a-octahydronaphtho[2,3-*b*]furan-2(3*H*)-one (**8**)

Colorless oil; IR (film) υ̃_max_ 1761, 1715 cm^–1^; ^1^H NMR (CDCl_3_, 500 MHz) δ 4.89 (1H, d *J* = 3.1 Hz, H-6), 4.81 (1H, brddd, *J* = 5.6, 3.2, 2.8 Hz, H-8), 3.30 (1H, ddd, *J* = 9.5, 8.7, 3.9 Hz, H-11), 3.26 (1H, ddd *J* = 8.7, 5.6, 3.1 Hz, H-7), 3.00 (1H, dd, *J* = 18.7, 3.9 Hz, H-13a), 2.66 (1H, dd, *J* = 18.7, 9.5 Hz, H-13b), 2.45 (1H, m, H-4), 2.25 (3H,s, H-17a, b, c), 2.11 (1H, dd, *J* = 14.8, 3.2 Hz, H-9a), 1.82 (1H, m, H-2a), 1.60 (1H, brddd, *J* = 12.8, 4.7, 3.3, Hz, H-1a), 1.54 (2H, m, H-3a, b), 1.52 (1H, dd, *J* = 14.8, 2.8 Hz, H-9b), 1.43 (1H, dddd, *J* = 13.7, 6.8, 3.5, 3.3 Hz, H-2b), 1.22 (3H, s, C-14a, b, c), 1.13 (3H,d, *J* = 7.6 Hz, H-15a, b, c), 1.12 (1H, m, H-1b); ^13^C NMR (CDCl_3_,125 MHz) δ 206.1 (C, C-16), 177.9 (C, C-12), 151.6 (C, C-5), 115.0 (CH, C-6), 77.6 (CH, C-8), 42.8 (CH_2_, C-9), 42.2 (CH_2_, C-1), 41.3 (CH, C-11), 40.0 (CH_2_, C-13), 38.5 (CH, C-4), 37.3 (CH, C-7), 33.1 (C, C-10), 32.8 (CH_2_, C-3), 30.0 (CH_3_, C-17), 28.7 (CH_3_, C-14), 23.2 (CH_3_, C-15), 16.8 (CH_2_, C-2); HRESIMS *m*/*z* 277.1800 [M + H]^+^ (calcd for C_17_H_25_O_3_, 277.1804).

#### (1a*R*,2*S*,5a*R*,6a*R*,9*S*,9a*R*,9b*S*)-2,5a-Dimethyl-9-(2-oxopropyl)octahydro-2*H*-oxireno[2′,3′:4,4a]naphtho[2,3-*b*]furan-8(9*H*)-one (**9**)

Crystalline solid, mp 115–119 °C; [α]_Na_^25^ +31.7 (*c* 0.480, CHCl_3_), IR (film) υ̃_max_ 1750, 1715 cm^–1^; ^1^H NMR (CDCl_3_, 500 MHz) δ 4.65 (1H, brdd, *J* = 7.7, 3.0, 2.7 Hz, H-8), 3.44 (1H, ddd, *J* = 10.8, 9.9, 4.1 Hz, H-11), 3.28 (1H, ddd, *J* = 10.8, 7.7, 1.2 Hz, H-7), 3.13 (1H, dd, *J* = 17.7, 4.1 Hz, H-13a), 2.79 (1H, dd, *J* = 18.7, 9.9 Hz, H-13b), 2.51 (1H, brs, H-6), 2.28 (3H, s, H-17a, b, c), 1.82 (1H, dd, *J* = 14.8, 3.0 Hz, H-9a), 1.82 (1H, m, H-3a), 1.81 (1H, m, H-2a), 1.60 (1H, dd, *J* = 14.8, 2.7 Hz, H-9b), 1.49 (1H, m, H-3b), 1.48 (1H, m, H-2b), 1.41 (2H, m, H-1a, b), 1.32 (1H, m, H-4), 1.20 (3H, s, H-14a, b, c), 1.12 (3H, d, *J* = 7.8 Hz, H-15a, b, c); ^13^C NMR (CDCl_3_, 125 MHz) δ 205.1 (C, C-16), 177.5 (C, C-12), 76.2 (CH, C-8), 68.3 (C, C-5), 57.6 (CH, C-6), 40.3 (CH_2_, C-13), 38.7 (CH_2_, C-9), 38.0 (CH, C-11), 37.81 (CH_2_, C-1), 37.75 (CH, C-4), 35.3 (CH, C-7), 32.1 (C, C-10), 29.9 (CH_3_, C-17), 29.7 (CH_2_, C-3), 24.0 (CH_3_, C-14), 17.8 (CH_3_, C-15), 16.5 (CH_2_, C-2); HRESIMS *m*/*z* 315.1553 [M + Na]^+^ (calcd for C_17_H_24_O_4_Na, 315.1572).

### Synthesis of the Ketal Derivatives (**10** and **11**)

Each of the 1,4-dicarbonyl derivatives **7** and **8** (100 mg each) was dissolved in a mixture of anhydrous MeOH/trimethyl orthoformate (due to the poor solubility of the molecules in the mixture, its volume and proportions varied according to the substrate used) in a 25 mL flask and a catalytic amount of *p*-TsOH was added. The reaction was stirred at rt for 12 h and then neutralized using 0.1 mL of trimethylamine. Saturated aqueous Na_2_CO_3_ solution (20 mL) was added to the crude and then it was extracted thrice using EtOAc. The organic layers were combined, dried by means of Na_2_SO_4_ and the solvent was evaporated under reduced pressure. The crude product was purified by column chromatography using a mixture of hexane:EtOAc 9:1 to 6:4 and the corresponding ketal derivative **10** at 58% yield (67.7 mg, 2.10 × 10^–1^ mmol) or the compound **11** at 65% yield (75.8 mg, 2.35 × 10^–1^ mmol) was obtained.

#### (3*S*,3a*R*,4a*S*,8a*R*,9a*R*)-3-(2,2-Dimethoxypropyl)-8a-methyl-5-methylenedecahydronaphtho[2,3-*b*]furan-2(3*H*)-one (**10**)

Crystalline solid; mp 118–122 °C; [α]_Na_^25^ +40.5 (*c* 0.074, CHCl_3_); IR (film) υ̃_max_ 1764 cm^–1^; ^1^H NMR (CDCl_3_, 500 MHz) δ 4.78 (1H, brs, H-15a), 4.47 (1H, brs, H-15b), 4.47 (1H, m, H-8), 2.76 (1H, m, H-11), 3.20 (3H, s, H-18a, b, c; H-19a, b, c), 2.48 (1H, dddd, *J* = 12.3, 10.5, 5.9, 4.6 Hz, H-7), 2.33 (brd, *J* = 12.5 Hz, H-3a), 2.25 (1H, brd, *J* = 14.9 Hz, H-13a), 2.17 (1H, brd, *J* = 15.3 Hz, H-9a), 2.00 (1H, brddd, *J* = 12.5, 12.2, 6.6 Hz, H-3b), 1.85 (1H, dd, *J* = 14.9, 8.8 Hz, H-13b), 1.80 (1H, brd, *J* = 12.3 Hz, H-5), 1.67 (1H, ddd, *J* = 13.2, 5.9, 2.2 Hz, H-6a), 1.58 (2H, m, H-2a, b), 1.54 (1H, m, H-1a), 1.46 (1H, dd, *J* = 15.3, 4.1 Hz, H-9b), 1.32 (3H, s, H-17a, b, c), 1.22 (1H, m, H-1b), 1.07 (1H, ddd, *J* = 13.2, 12.3, 12.3 Hz, H-6b), 0.79 (3H, s, H-14a, b, c); ^13^C NMR (CDCl_3_, 125 MHz) δ 178.7 (C, C-12), 149.4 (C, C-4), 106.5 (CH_2_, C-15), 101.1 (C, C-16), 78.0 (CH, C-8), 48.5 (CH_3_, C-18), 48.3 (CH_3_, C-19), 46.6 (CH, C-5), 43.7 (CH, C-11), 42.2 (CH_2_, C-1), 41.6 (CH_2_, C-9), 40.0 (CH, C-7), 36.8 (CH_2_, C-3), 34.9 (C, C-10), 30.7 (CH_2_, C-13), 22.7 (CH_2_, C-2), 21.8 (CH_2_, C-6), 21.3 (CH_3_, C-17), 17.8 (CH_3_, C-14); HRESIMS *m*/*z* 345.2041 [M + Na]^+^ (calcd for C_19_H_30_O_4_Na, 345.2042).

#### (3*S*,3a*R*,5*S*,8a*R*,9a*R*)-3-(2,2-Dimethoxypropyl)-5,8a-dimethyl-3a,5,6,7,8,8a,9,9a-octahydronaphtho[2,3-*b*]furan-2(3*H*)-one (**11**)

Colorless oil; [α]_Na_^25^ +62.5 (*c* 0.470, CHCl_3_); IR (film) υ̃_max_ 1741 cm^–1^; ^1^H NMR (C_6_D_6_, 500 MHz) δ 4.95 (1H, brs, H-6), 4.27 (1H, m, H-8), 3.48 (3H, s, H-18a, b, c), 3.04 (3H, s, H-19a, b, c), 2.92 (1H, m, H-7), 2.43 (1H, m, H-4), 2.28 (1H, brddd, *J* = 5.3, 5.0, 3.6 Hz, H-11), 2.21 (1H, ddd, *J* = 13.4, 5.0, 0.9 Hz, H-13a), 1.80 (1H, ddd, *J* = 13.3, 3.8, 3.8 Hz, H-2a), 1.75 (1H, dd, *J* = 14.2, 3.7 Hz, H-9a), 1.70 (1H, dd, *J* = 13.4, 5.3 Hz, H-13b), 1.52 (2H, m, H-3a, b), 1.50 (1H, m, H-1a), 1.45 (1H, dd, *J* = 14.2, 2.6 Hz. H-9b), 1.42 (3H, s, H-14a, b, c), 1.33 (1H, m, H-2b), 1.07 (1H, m, H-1b), 1.19 (3H, d, *J* = 7.6 Hz, H-15a, b, c), 1.11 (3H, s, H-17a, b, c); ^13^C NMR (C_6_D_6_, 125 MHz) δ 174.4 (C, C-12), 122.0 (CH, C-6), 97.8 (C, C-16), 64.5 (CH, C-8), 51.2 (CH_3_, C-18), 47.6 (CH_3_, C-19), 45.9 (CH_2_, C-9), 43.4 (CH_2_, C-1), 41.4 (CH, C-11), 39.1 (CH, C-4), 35.3 (CH, C-7), 34.3 (CH_2_, C-13), 34.2 (C, C-10), 33.8 (CH_2_, C-3), 28.3 (CH_3_, C-14), 23.2 (CH_3_, C-17), 22.8 (CH_3_, C-15), 17.7 (CH_2_, C-2); HRESIMS *m*/*z* 345.2031 [M + Na]^+^ (calcd for C_19_H_30_O_4_Na, 345.2042).

### Synthesis of the Hydroxylated Ketal Derivatives (**12** and **13**)

Each of the ketal derivatives **10** or **11** (100 mg each) was dissolved in 25 mL of dry THF in a 100 mL flask at −73 °C using a liquid air/acetone bath under a nitrogen atmosphere. KHDMS solution (0.7 M; 4.5 mL) was slowly added into the mixture and allowed to react for 30 min while keeping the temperature at −73 °C. After this time, dry oxygen was directly bubbled into the reaction mixture for 1 h. Then, 50 μL of triethyl phosphite were added and the mixture was slowly warmed to −20 °C. After reaching this temperature, 20 mL of Sörensen buffer (945 mg of anhydrous Na_2_HPO_4_ in 50 mL of H_2_O + 908 mg of anhydrous KH_2_PO_4_ in 50 mL of H_2_O, pH = 7.2) were added and the mixture was extracted thrice using 20 mL of EtOAc. The combined organic layers were dried with anhydrous Na_2_SO_4_ and the solvent was evaporated under reduced pressure. The crude product was purified by column chromatography with a mixture of hexane:EtOAc 9:1 to 6:4, the corresponding hydroxylated ketal derivatives **12** at 63% yield (73.5 mg, 2.18 × 10^–1^ mmol) along with 22% of the compound **1** (13.3 mg, 4.58 × 10^–2^ mmol) or **13** at 62% yield (72.0 mg, 2.14 × 10^–1^ mmol) along with a 18% yield of the compound **2** (10.1 mg, 3.48 × 10^–2^ mmol) were obtained.

#### (3*R*,3a*S*,4a*S*,8a*R*,9a*R*)-3-(2,2-Dimethoxypropyl)-3-hydroxy-8a-methyl-5-methylenedecahydronaphtho[2,3-*b*]furan-2(3*H*)-one (**12**)

The physical properties of **12** were not determined because it was not possible to fully purify it from **1**.

#### (3*R*,3a*S*,5*S*,8a*R*,9a*R*)-3-(2,2-Dimethoxypropyl)-3-hydroxy-5,8a-dimethyl-3a,5,6,7,8,8a,9,9a-octahydronaphtho[2,3-*b*]furan-2(3*H*)-one (**13**)

The physical properties of the compound **13** were not determined since it could not be fully separated from compound **2**.

### Synthesis of the Dihydroxylated Ketal (**15**)

(11*S*)-11-Hydroxy-13-((2-methyl-1,3-dioxolan-2-yl)methyl)costuslactone **(14)** (100 mg, 2.99 × 10^–1^ mmol) was dissolved in 15 mL of CHCl_3_ in a 50 mL flask. SeO_2_ (8.3 mg, 7.44 × 10^–2^ mmol) was added to the mixture and stirred vigorously. Then, 280 μL (2.91 mmol) of *tert*-butyl hydroperoxide were added dropwise over 10 min (28 μL/min). Once the addition had been completed, the reaction was kept stirring for 35 min at rt. The crude product was filtered through silica gel to eliminate the SeO_2_. Then, the solvent was evaporated under reduced pressure and the crude product was purified by column chromatography with a mixture of hexane:EtOAc 9:1 to 6:4. The compound **15** was obtained in 59% yield (62.5 mg, 1.87 × 10^–1^ mmol).

#### (3*R*,3a*R*,6a*R*,8*R*,9a*R*,9b*R*)-3,8-Dihydroxy-3-((2-methyl-1,3-dioxolan-2-yl)methyl)-6,9-dimethylenedecahydroazuleno[4,5-*b*]furan-2(3*H*)-one (**15**)

Colorless oil; [α]_Na_^25^ +21.1 (*c* 0.146, CHCl_3_); IR (film) υ̃_max_ 3402, 1764 cm^–1^; ^1^H NMR (CDCl_3_, 500 MHz) δ 5.46 (1H, brdd, *J* = 2.0, 1.4 Hz, H-15a), 5.36 (1H, brdd, *J* = 2.0, 1.4 Hz, H-15b), 4.91 (1H, s, H-14a), 4.77 (1H, s, H-14b), 4.67 (1H, brddd, *J* = 6.5, 5.5, 1.4 Hz, H-3), 4.12 (1H, dd, *J* = 9.6, 9.6 Hz, H-6), 3.98 (3H, m, H-18a, b, c; H-19-a, b, c), 3.09 (1H, brddd, *J* = 9.2, 7.6, 6.1 Hz, H-1), 2.97 (1H, brddd, *J* = 9.6, 9.2, 1.6 Hz, H-5), 2.51 (1H, ddd, *J* = 12.6, 4.8, 4.8 Hz, H-9a), 2.30 (1H, d, *J* = 14.8 Hz. H-13a), 2.26 (1H, ddd, *J* = 11.7, 9.6, 3.6 Hz, H-7), 2.19 (1H, d, *J* = 14.8 Hz, H-13b), 2.15 (1H, ddd, *J* = 13.6, 6.5, 6.1 Hz, H-2a), 1.99 (1H, ddd, *J* = 12.6, 12.0, 4.6 Hz, H-9b), 1.92 (1H, dddd, *J* = 13.2, 4.8, 4.6, 3.6 Hz, H-8a), 1.88 (1H, ddd, *J* = 13.6, 7.6, 5.5 Hz, H-2b), 1.63 (1H, dddd, *J* = 13.2, 12.0, 11.7, 4.8 Hz, H-8b), 1.42 (3H, s, H-17a, b, c); ^13^C NMR (CDCl_3_, 125 MHz) δ 176.6 (C, C-12), 154.4 (C, C-4), 149.1 (C, C-10), 113.2 (CH_2_, C-15), 112.5 (CH_2_, C-14), 108.6 (C, C-16), 83.7 (CH, C-6), 75.4 (C, C-11), 74.4 (CH, C-3), 64.3 (CH_2_, C-18), 64.0 (CH_2_, C-19), 49.9 (CH, C-5), 49.8 (CH, C-7), 44.2 (CH, C-1), 42.5 (CH_2_, C-13), 39.6 (CH_2_, C-2), 36.5 (CH_2_, C-9), 25.7 (CH_3_, C-17), 25.6 (CH_2_, C-8); HRESIMS *m*/*z* 373.1631 [M + Na]^+^ (calcd for C_19_H_26_O_6_Na, 373.1627).

### Synthesis of the Hydroxylated 1,4-Dicarbonyl Derivatives (**1**, **2**, and **16**)

Each hydroxylated ketal derivative **12**, **13**, or **15** (100 mg each) was dissolved in 2 mL of an acetone:H_2_O 95:5 mixture in a 25 mL flask and catalytic amounts of *p*-TsOH were added. The mixture was stirred overnight at rt. A saturated aqueous NaHCO_3_ solution (2 mL) was added and the crude product was extracted thrice using EtOAc. The combined organic layers were dried with anhydrous Na_2_SO_4_ and the solvent was evaporated under reduced pressure. The crude product was purified by column chromatography with a mixture of hexane:EtAOc 9:1 to 6:4; 89% yield of pertyolide A (**1**) (76.6 mg, 2.62 × 10^–1^ mmol), 95% yield of Pertyolide B (**2**) (82.5 mg, 2.82 × 10^–1^ mmol) and 93% yield of the dihydroxylated 1,4-dicarbonyl derivative **16** (81.3 mg, 2.65 × 10^–1^ mmol) were obtained.

#### Pertyolide A (**1**)

Crystalline solid; [α]_Na_^25^ +39.3 (*c* 0.115, CHCl_3_), lit [α]_D_^25^ +42.0 (*c* 0.100, MeOH);^18^^1^H NMR (CDCl_3_, 500 MHz) δ 5.04 (1H, ddd, *J* = 4.2, 4.1, 2.0 Hz, H-8), 4.79 (1H, brdd, *J* = 1.5, 1.4 Hz, H-15a), 4.43 (1H, brdd, *J* = 1.5, 1.4 Hz, H-15b), 3.00 (1H, d, *J* = 17.4 Hz, H-13a), 2.64 (1H, d, *J* = 17.4 Hz, H-13b), 2.39 (1H, ddd, *J* = 12.8, 6.1, 4.1 Hz, H-7), 2.34 (3H, s, H-17a, b, c), 2.32 (1H, ddd, *J* = 12.8, 4.2, 2.2 Hz, H-3a), 2.20 (1H, dd, *J* = 15.6, 2.0 Hz, H-9a), 2.00 (1H, ddd, *J* = 12.8, 12.6, 5.8 Hz, H-3b), 1.80 (1H, brdd, *J* = 12.3, 1.4 Hz, H-5), 1.58 (2H, m, H-2a, b), 1.53 (1H, m, H-1a), 1.46 (1H, dd, *J* = 15.6, 4.2 Hz, H-9b), 1.42 (1H, ddd, *J* = 13.1, 6.1, 2.5 Hz, H-6a), 1.24 (1H, m, H-1b), 1.05 (1H, ddd, *J* = 13.1, 12.8, 12.3 Hz, H-6b), 0.78 (3H, s, H-14a, b, c); ^13^C NMR (CDCl_3_, 125 MHz) δ 210.3 (C, C-16), 175.4 (C, C-12), 149.2 (C, C-4), 106.3 (CH_2_, C-15), 79.5 (C, C-11), 77.5 (CH, C-8), 46.8 (CH, C-7), 46.5 (CH, C-5), 42.1 (CH_2_, C-1), 42.0 (CH_2_, C-13), 41.2 (CH_2_, C-9), 36.7 (CH_2_, C-3), 34.5 (C, C-10), 31.9 (CH_3_, C-17), 22.6 (CH_2_, C-2), 21.1 (CH_2_, C-6), 17.8 (CH_3_, C-14); HRESIMS *m*/*z* 315.1558 [M + Na]^+^ (calcd for C_19_H_26_O_6_Na, 315.1572).

#### Pertyolide B (**2**)

Crystalline solid; mp 101–103 °C; [α]_Na_^25^ −20.8 (*c* 0.292, CHCl_3_), lit [α]_D_^25^ +1.0 (*c* 0.100, MeOH);^18^^1^H NMR (CDCl_3_, 500 MHz) δ 5.13 (1H, brddd, *J* = 5.5, 3.3, 2.7 Hz, H-8), 4.94 (1H, d, *J* = 3.5 Hz, H-6), 3.01 (1H, dd, *J* = 5.5, 3.5 Hz, H-7), 2.95 (1H, d, *J* = 17.5 Hz, H-13a), 2.65 (1H, d, *J* = 17.5 Hz, H-13b), 2.46 (1H, m, H-4), 2.14 (1H, dd, *J* = 14.9, 3.3 Hz, H-9a), 2.33 (3H, s, H-17a, b, c), 1.82 (1H, ddddd, *J* = 13.7, 12.9, 12.6, 4.9, 3.4 Hz, H-2a), 1.59 (1H, m, H-1a), 1.56 (2H, m, H-3a, b), 1.51 (1H, dd, *J* = 14.9, 2.7 Hz, H-9b), 1.43 (1H, dddd, *J* = 13.7, 7.2, 3.5, 3.4 Hz, H-2b), 1.21 (3H, s, H-14a, b, c), 1.13 (3H, d, *J* = 7.5 Hz, H-15a, b, c), 1.12 (1H, m, H-1b); ^13^C NMR (CDCl_3_, 125 MHz) δ 210.3 (C, C-16), 175.3 (C, C-12), 152.6 (C, C-5), 113.5 (CH, C-6), 79.0 (C, C-11), 77.1 (CH, C-8), 46.9 (CH, C-7), 43.4 (CH_2_, C-13), 42.5 (CH_2_, C-9), 42.1 (CH_2_, C-1), 38.6 (CH, C-4), 33.0 (C, C-10), 32.8 (CH_2_, C-3), 31.8 (CH_3_, C-17), 28.6 (CH_3_, C-14), 23.0 (CH_3_, C-15), 16.7 (CH_2_, C-2); HRESIMS *m*/*z* 293.1753 [M + H]^+^ (calcd for C_17_H_25_O_4_, 293.1753).

#### (3*R*,3a*R*,6a*R*,8*R*,9a*R*,9b*R*)-3,8-Dihydroxy-6,9-dimethylene-3-(2-oxopropyl)decahydroazuleno[4,5-*b*]furan-2(3*H*)-one (**16**)

Colorless oil; [α]_Na_^25^ +10.5 (*c* 0.166, CHCl_3_); IR (film) υ̃_max_ 3386, 1763, 1706 cm^–1^; ^1^H NMR (CDCl_3_, 500 MHz) δ 5.46 (1H, brdd, *J* = 2.0, 1.3 Hz, H-15a), 5.37 (1H, brdd, *J* = 2.0, 1.3 Hz, H-15b), 4.92 (1H, s, H-14a), 4.79 (1H, s, H-14b), 4.66 (1H, brddd, *J* = 6.3, 5.2, 1.3 Hz, H-3), 4.23 (1H, dd, *J* = 9.6, 9.6 Hz, H-6), 3.08 (1H, m, H-1), 3.00 (1H, brddd, *J* = 11.0, 9.6, 1.7 Hz, H-5), 2.81 (1H, d, *J* = 16.7 Hz, H-13a), 2.64 (1H, d, *J* = 16.7 Hz, H13b), 2.54 (1H, ddd, *J* = 12.9, 4.6, 4.6 Hz, H-9a), 2.31 (3H, s, H-17a, b, c), 2.13 (1H, ddd, *J* = 13.6, 6.3, 6.3 Hz, H-2a), 1.97 (1H, m, H-7), 1.96 (1H, m, H-9b), 1.88 (1H, ddd, *J* = 13.6, 7.7, 5.2 Hz, H-2b), 1.74 (2H, m, H-8a, b); ^13^C NMR (CDCl_3_, 125 MHz) δ 209.9 (C, C-16), 175.8 (C, C-12), 154.1 (C, C-4), 148.7 (C, C-10), 113.4 (CH_2_, C-15), 112.8 (CH_2_, C-14), 83.9 (CH, C-6), 76.3 (C, C-11), 74.3 (CH, C-3), 51.7 (CH, C-7), 49.5 (CH, C-5), 44.4 (CH_2_, C-13), 44.1 (CH, C-1), 39.5 (CH_2_, C-2), 36.2 (CH_2_, C-9), 32.1 (CH_3_, C-17), 25.5 (CH_2_, C-8); HRESIMS *m*/*z* 329.1371 [M + Na]^+^ (calcd for C_17_H_22_O_5_Na, 329.1365).

### Synthesis of Pertyolide C (**3**)

Triphenylphosphine (92 mg, 3.59 × 10^–1^ mmol) was dissolved in 2 mL of anhydrous THF in a 25 mL flask under a nitrogen atmosphere. Isovaleric acid (39 μL, 3.59 × 10^–1^ mmol) was added into the mixture and then it was cooled to 0 °C using an ice/water bath. Compound **16** (100 mg, 3.26 × 10^–1^ mmol) dissolved in 1 mL of anhydrous THF (under a nitrogen atmosphere) was added to the mixture. Then, 69 μL (3.59 × 10^–1^ mmol) of diisopropylazodicarboxylate were also added and the mixture was allowed to react for 90 min at rt. When the reaction was completed, the solvent was evaporated under reduced pressure and the crude was redissolved in EtOAc and filtered through silica to eliminate the triphenylphosphine oxide generated. The crude product was purified by column chromatography with a mixture of hexane:EtOAc 9:1 to 6:4. Pertyolide C (**3**) was obtained at 43% yield (54.8 mg, 1.40 × 10^–1^ mmol).

#### Pertyolide C (**3**)

Colorless oil; [α]_Na_^25^ +72.9 (*c* 0.300, CHCl_3_), lit [α]_D_^25^ +17.0 (*c* 0.100, MeOH);^18^^1^H NMR (CDCl_3_, 500 MHz) δ 5.55 (1H, dddd, *J* = 7.9, 6.7, 2.0, 2.0 Hz, H-3), 5.42 (1H, dd, *J* = 2.0, 2.0 Hz, H-15a), 5.29 (1H, dd, *J* = 2.0, 2.0 Hz, H-15b), 4.92 (1H, s, H-14a), 4.91 (1H, s, H-14b), 4.36 (1H, dd, *J* = 9.6, 9.6 Hz, H-6), 2.87 (1H, ddd, *J* = 8.9, 8.1, 7.9 Hz, H-1), 2.80 (1H, d, *J* = 16.8 Hz, H-13a), 2.75 (1H, brdddd, *J* = 9.6, 8.9, 2.0, 2.0 Hz, H-5), 2.63 (1H, d, *J* = 16.8 Hz, H-13b), 2.49 (1H, ddd, *J* = 13.0, 5.2, 5.2 Hz, H-9a), 2.44 (1H, ddd, *J* = 13.9, 7.9, 7.9 Hz, H-2a), 2.31 (3H, s, H-17a, b, c), 2.23 (2H, brdd, *J* = 7.2, 1.7 Hz, H-2′a, b), 2.12 (1H, brsepd, *J* = 6.6, 0.8 Hz, H-3′), 1.99 (1H, ddd, *J* = 13.0, 9.8, 5.3 Hz, H-9b), 1.96 (1H, ddd, *J* = 10.5, 9.6, 5.0 Hz, H-7), 1.78 (1H, m, H-8), 1.76 (1H, m, H-2b), 0.97 (3H, d, *J* = 6.6 Hz, H-4′a, b, c; H-5′a, b, c); ^13^C NMR (CDCl_3_, 125 MHz) δ 209.9 (C, C-16), 175.8 (C, C-12), 172.9 (C, C-1′), 148.2 (C, C-10), 148.0 (C, C-4), 114.2 (CH_2_, C-15), 113.8 (CH_2_, C-14), 82.9 (CH, C-6), 76.2 (C, C-11), 74.3 (CH, C-3), 51.7 (CH, C-7), 50.1 (CH, C-5), 44.3 (CH_2_, C-13), 44.2 (CH, C-1), 43.6 (CH_2_, C-2′), 36.2 (CH_2_, C-2), 34.5 (CH_2_, C-9), 32.0 (CH_3_, C-17), 25.7 (CH, C-3′), 25.0 (CH_2_, C-8), 22.40 (CH_3_, C-4′), 22.37 (CH_3_, C-5′); HRESIMS *m*/*z* 413.1938 [M + Na]^+^ (calcd for C_22_H_30_O_6_Na, 413.1940).

### Coleoptile Bioassay

Wheat seeds (*Triticum aestivum* L. cv. Burgos) were sown in water-moistened 15 cm diameter Petri dishes and grown in the dark at 25 ± 1 °C for 4 days. The roots and caryopsis were removed from the shoots. The latter had 2.0 mm of their apexes cut off by means of a van der Weij guillotine and discarded. The adjacent 4.0 mm sections of the coleoptiles were used for the bioassay. All the manipulations were performed under green safelight to prevent their growth.^36^ The compounds were predissolved in DMSO and diluted in a phosphate/citrate buffer solution containing 2% sucrose at pH 5.6 to obtain the final concentrations for the assays (1000, 300, 100, 30, and 10 μM) with a consistent 0.1% DMSO content.

Different solutions of the standard commercialized herbicide Logran (active ingredients 59.4% terbutryn and 0.6% triasulfuron) at the aforementioned concentrations were also prepared to be used as the positive control samples. A solution of 0.1% DMSO in the phosphate/citrate buffer was also prepared to be used as the negative control samples.

Five coleoptiles (4.0 mm length) were placed in different test tubes and 2.0 mL of either the test compounds, the positive or the negative control samples were added into the tubes (three replicates per dilution). The tubes were then rotated at 0.25 rpm for 24 h at 25 ± 1 °C in the dark by means of a tube roller device.

After this time, the length of the coleoptiles was digitalized and its statistical evaluation was performed by means of Welch’s test.^37^ The results were expressed as percentage differences from the negative control, so that the positive values would represent stimulation and the negative values would indicate the inhibition level achieved against the elongation of the coleoptiles.

## Supporting Information Available

NMR tables, 1D and 2D NMR, and FTIR spectra (PDF)
