# Supplementary material for: Synthesis of Pertyolides A, B, and C: A Synthetic Procedure to C17-Sesquiterpenoids and a Study of Their Phytotoxic Activity
Source: J Nat Prod. 2021 Aug 9;84(8):2295–302. doi: 10.1021/acs.jnatprod.1c00396 (PMC8638260; doi:10.1021/acs.jnatprod.1c00396)
Supplement: Supplementary file 1 — np1c00396_si_001.pdf [file np1c00396_si_001.pdf]

SUPPORT INFORMATION FOR

SYNTHESIS OF PERTYOLIDES A, B AND C: A SYNTHETIC PROCEDURE TO C_{17}-SESQUITERPENOIDS AND A STUDY OF THEIR PHYTOTOXIC ACTIVITY

David M. Cárdenas, Carlos Rial, Rosa M. Varela, José M. G. Molinillo, Francisco A. Macías*

Allelopathy Group, Department of Organic Chemistry, Institute of Biomolecules (INBIO), Campus CEIA3, School of Science, University of Cadiz. C/ República Saharaui, 7, 11510, Puerto Real (Cádiz), Spain

Table of Contents

Table S1. ^{1}H NMR and ^{13}C NMR (500/125 MHz) Data for derivatives **1** and **2**, $CDCl_3$ S1
Table S2. ^{1}H NMR and ^{13}C NMR (500/125 MHz) Data for the compounds **3** and **9**, $CDCl_3$ S2
Table S3. ^{1}H NMR and ^{13}C NMR Data for derivatives **7** and **8**, $CDCl_3$ S3
Table S4. ^{1}H NMR and ^{13}C NMR (500/125 MHz) Data for derivatives **10** and **11** S4
Table S5. ^{1}H NMR and ^{13}C NMR (500/125 MHz) Data for derivatives **15** and **16**, $CDCl_3$ S5
Table S6. ^{1}H NMR and ^{13}C NMR comparison for synthetized and isolated **1** S6
Table S7. ^{1}H NMR and ^{13}C NMR comparison for synthetized and isolated **2** S7
Table S8. ^{1}H NMR and ^{13}C NMR comparison for synthetized and isolated **3** S8
Figure S1. ^{1}H-NMR (500 MHz) spectrum of **1** in $CDCl_3$ S9
Figure S2. ^{13}CNMR (125 MHz) spectrum of **1** in $CDCl_3$ S10
Figure S3. ^{1}H-^{1}H-COSY spectrum of **1** in $CDCl_3$ S11
Figure S4. HSQC spectrum of **1** in $CDCl_3$ S12
Figure S5. HMBC spectrum of **1** in $CDCl_3$ S13
Figure S6. IR spectrum of **1** S14
Figure S7. ^{1}H-NMR (500 MHz) spectrum of **2** in $CDCl_3$ S15
Figure S8. ^{13}CNMR (125 MHz) spectrum of **2** in $CDCl_3$ S16
Figure S9. ^{1}H-^{1}H-COSY spectrum of **2** in $CDCl_3$ S17
Figure S10. HSQC spectrum of **2** in $CDCl_3$ S18
Figure S11. HMBC spectrum of **2** in $CDCl_3$ S19
Figure S12. IR spectrum of **2** S20
Figure S13. ^{1}H-NMR (500 MHz) spectrum of **3** in $CDCl_3$ S21
Figure S14. ^{13}CNMR (125 MHz) spectrum of **3** in $CDCl_3$ S22
Figure S15. ^{1}H-^{1}H-COSY spectrum of **3** in $CDCl_3$ S23
Figure S16. HSQC spectrum of **3** in $CDCl_3$ S24
Figure S17. HMBC spectrum of **3** in $CDCl_3$ S25
Figure S18. 1D-NOESY spectrum of **3** in $CDCl_3$ S26
Figure S19. IR spectrum of **3** S27
Figure S20. ^{1}H-NMR (500 MHz) spectrum of **4** in $CDCl_3$ S28
Figure S21. ^{13}CNMR (125 MHz) spectrum of **4** in $CDCl_3$ S29
Figure S22. ^{1}H-^{1}H-COSY spectrum of **4** in $CDCl_3$ S30
Figure S23. HSQC spectrum of **4** in $CDCl_3$ S31
Figure S24. HMBC spectrum of **4** in $CDCl_3$ S32
Figure S25. IR spectrum of **4** S33
Figure S26. ^{1}H-NMR (500 MHz) spectrum of **5** in $CDCl_3$ S34
Figure S27. ^{13}CNMR (125 MHz) spectrum of **5** in $CDCl_3$ S35
Figure S28. ^{1}H-^{1}H-COSY spectrum of **5** in $CDCl_3$ S36
Figure S29. HSQC spectrum of **5** in $CDCl_3$ S37
Figure S30. HMBC spectrum of **5** in $CDCl_3$ S38
Figure S31. IR spectrum of **5** S39
Figure S32. ^{1}H-NMR (400 MHz) spectrum of **7** in $CDCl_3$ S40
Figure S33. ^{13}CNMR (100 MHz) spectrum of **7** in $CDCl_3$ S41
Figure S34. ^{1}H-^{1}H-COSY spectrum of **7** in $CDCl_3$ S42
Figure S35. HSQC spectrum of **7** in $CDCl_3$ S43
Figure S36. HMBC spectrum of **7** in $CDCl_3$ S44
Figure S37. 1D-NOESY spectrum of **7** in $CDCl_3$ S45
Figure S38. IR spectrum of **7** S46
Figure S39. ^{1}H-NMR (500 MHz) spectrum of **8** in $CDCl_3$ S47
Figure S40. ^{13}CNMR (125 MHz) spectrum of **8** in $CDCl_3$ S48
Figure S41. ^{1}H-^{1}H-COSY spectrum of **8** in $CDCl_3$ S49
Figure S42. HSQC spectrum of **8** in $CDCl_3$ S50

Figure S43. HMBC spectrum of **8** in $CDCl_3$ S51
Figure S44. 1D-NOESY spectrum of **8** in $CDCL_3$ S52
Figure S45. IR spectrum of **8** S53
Figure S46. ^{1}H-NMR (500 MHz) spectrum of **9** in $CDCl_3$ S54
Figure S47. ^{13}CNMR (125 MHz) spectrum of **9** in $CDCl_3$ S55
Figure S48. ^{1}H-^{1}H-COSY spectrum of **9** in $CDCl_3$ S56
Figure S49. HSQC spectrum of **9** in $CDCl_3$ S57
Figure S50. HMBC spectrum of **9** in $CDCl_3$ S58
Figure S51. 1D-NOESY spectrum of **9** in $CDCl_3$ S59
Figure S52. IR spectrum of **9** S60
Figure S53. 3D model of **9** S61
Figure S54. ^{1}H-NMR (500 MHz) spectrum of **10** in $CDCl_3$ S62
Figure S55. ^{13}CNMR (125 MHz) spectrum of **10** in $CDCl_3$ S63
Figure S56. ^{1}H-^{1}H-COSY spectrum of **10** in $CDCl_3$ S64
Figure S57. HSQC spectrum of **10** in $CDCl_3$ S65
Figure S58. HMBC spectrum of **10** in $CDCl_3$ S66
Figure S59. IR spectrum of **10** S67
Figure S60. ^{1}H-NMR (500 MHz) spectrum of **11** in C_6D_6 .... S68
Figure S61. ^{13}CNMR (125 MHz) spectrum of **11** in C_6D_6 .... S69
Figure S62. ^{1}H-^{1}H-COSY spectrum of **11** in C_6D_6 .... S70
Figure S63. HSQC spectrum of **11** in C_6D_6 .... S71
Figure S64. HMBC spectrum of **11** in C_6D_6 .... S72
Figure S65. IR spectrum of **11** S73
Figure S66. ^{1}H-NMR (500 MHz) spectrum of **15** in $CDCl_3$ S74
Figure S67. ^{13}CNMR (125 MHz) spectrum of **15** in $CDCl_3$ S75
Figure S68. ^{1}H-^{1}H-COSY spectrum of **15** in $CDCl_3$ S76
Figure S69. HSQC spectrum of **15** in $CDCl_3$ S77
Figure S70. HMBC spectrum of **15** in $CDCl_3$ S78
Figure S71. IR spectrum of **15** S79
Figure S72. ^{1}H-NMR (500 MHz) spectrum of **16** in $CDCl_3$ S80
Figure S73. ^{13}CNMR (125 MHz) spectrum of **16** in $CDCl_3$ S81
Figure S74. ^{1}H-^{1}H-COSY spectrum of **16** in $CDCl_3$ S82
Figure S75. HSQC spectrum of **16** in $CDCl_3$ S83
Figure S76. HMBC spectrum of **16** in $CDCl_3$ S84
Figure S77. IR spectrum of **16** S85

Table S1. ^{1}H NMR and ^{13}C NMR (500/125 MHz) Data for derivatives **1** and **2**, $CDCl_3$.

	**1**		**2**	
Position	δ_C, type	δ_H (J in Hz)	δ_C, type	δ_H (J in Hz)
1	42.1, CH_2	1.53, m<br>1.24, m	42.1, CH_2	1.59, m<br>1.12, m
2	22.6, CH_2	1.58, m	16.7, CH_2	1.82, ddddd (13.7, 12.9, 12.6, 4.9, 3.4)<br>1.43, dddd (13.7, 7.2, 3.5, 3.4)
3	36.7, CH_2	2.32, ddd (12.8, 4.2, 2.2)<br>2.00, ddd (12.8, 12.6, 5.8)	32.8, CH_2	1.56, m
4	149.2, C	-	38.6, CH	2.46, m
5	46.5, CH	1.80, brdd (12.3, 1.4)	152.6, C	-
6	21.1, CH_2	1.42, ddd (13.1, 6.1, 2.5)<br>1.05, ddd (13.1, 12.8, 12.3)	113.5, CH	4.94, d (3.5)
7	46.8, CH	2.39, ddd (12.8, 6.1, 4.1)	46.9, CH	3.01, dd (5.5, 3.5)
8	77.5, CH	5.04, ddd (4.2, 4.1, 2.0)	77.1, CH	5.13, brddd (5.5, 3.3, 2.7)
9	41.2, CH_2	2.20, dd (15.6, 2.0)<br>1.46, dd (15.6, 4.2)	42.5, CH_2	2.14, dd (14.9, 3.3)<br>1.51, dd (14.9, 2.7)
10	34.5, C	-	33.0, C	-
11	79.5, C	-	79.0, C	-
12	175.4, C	-	175.3, C	-
13	42.0, CH_2	3.00, d (17.4)<br>2.64, d (17.4)	43.4, CH_2	2.95, d (17.5)<br>2.65, d (17.5)
14	17.8, CH_3	0.78, s	28.6, CH_3	1.21, s
15	106.3, CH_2	4.79, brdd (1.5, 1.4)<br>4.43, brdd (1.5, 1.4)	23.0, CH_3	1.13, d (7.5)
16	210.3, C	-	210.3, C	-
17	31.9, CH_3	2.34, s	31.8, CH_3	2.33, s

Table S2. ^{1}H NMR and ^{13}C NMR (500/125 MHz) Data for the compounds **3** and **9**, $CDCl_3$.

	**3**		**9**	
Position	δ_C, type	δ_H (J in Hz)	δ_C, type	δ_H (J in Hz)
1	44.2, CH	2.87, ddd (8.9, 8.1, 7.9)	37.81, CH_2	1.41, m
2	36.2, CH_2	2.44, ddd (13.9, 7.9, 7.9)<br>1.76, m	16.5, CH_2	1.81, m<br>1.48, m
3	74.3, CH	5.55, brdddd (7.9, 6.7, 2.0. 2.0)	29.7, CH_2	1.82, m<br>1.49, m
4	148.0, C	-	37.75, CH	1.32, m
5	50.1, CH	2.75, brdddd (9.6, 8.9, 2.0, 2.0)	68.3, C	-
6	82.9, CH	4.36, dd (9.6, 9.6)	57.6, CH	2.51, brs
7	51.7, CH	1.96, ddd (10.5, 9.6, 5.0)	35.3, CH	3.28, ddd (10.8, 7.7, 1.2)
8	25.0, CH_2	1.78, m	76.2, CH	4.65, brdd (7.7, 3.0, 2.7)
9	34.5, CH_2	2.49, ddd (13.0, 5.2, 5.2)<br>1.99, ddd (13.0, 9.8, 5.3)	38.7, CH_2	1.82, dd (14.8, 3.0)<br>1.60, dd (14.8, 2.7)
10	148.2, C	-	32.1, C	-
11	76.2, C	-	38.0, CH	3.44, ddd (10.8, 9.9, 4.1)
12	175.8, C	-	177.5, C	-
13	44.3, CH_2	2.80, d (16.8)<br>2.63, d (16.8)	40.3, CH_2	3.13, dd (18.7, 4.1)<br>2.79, dd (18.7, 9.9)
14	113.8, CH_2	4.92, s<br>4.91, s	24.0, CH_3	1.20, s
15	114.2, CH_2	5.42, dd (2.0, 2.0)<br>5.29, dd (2.0, 2.0)	17.8, CH_3	1.12, d (7.8)
16	209.9, C	-	205.1, C	-
17	32.0, CH_3	2.31, s	29.9, CH_3	2.28, s
1’	172.9, C	-	-	-
2’	43.6, CH_2	2.23, brdd (7.2, 1.7)	-	-
3’	25.7, CH	2.12, brsepd (6.6, 0.8)	-	-
4’[a]	22.40, CH_3	0.97, d (6.6)	-	-
5’[a]	22.37, CH_3	0.97, d (6.6)	-	-

[a] Interchangeable signals.

Table S3. ^{1}H NMR and ^{13}C NMR Data for derivatives **7** and **8**, $CDCl_3$.

	**7**[a]		**8**[b]	
Position	δ_C, type	δ_H (J in Hz)	δ_C, type	δ_H (J in Hz)
1	42.0, CH_2	1.48, m<br>1.19, m	42.2, CH_2	1.60, brddd (12.8, 4.7, 3.3)<br>1.12, m
2	22.5, CH_2	1.52, m	16.8, CH_2	1.82, m<br>1.43, dddd (13.7, 6.8, 3.5, 3.3)
3	36.6, CH_2	2.28, m<br>1.94, ddd (18.6, 12.7, 5.8)	32.8, CH_2	1.54, m
4	149.2, C	-	38.5, CH	2.45, m
5	46.2, CH	1.74, brd (12.5)	151.6, C	-
6	21.3, CH_2	1.33, ddd (13.2, 6.2, 2.4)<br>1.03, ddd (13.2, 12.5, 12.3)	115.0, CH	4.89, d (3.1)
7	38.7, CH	2.56, dddd (12.3, 6.4, 6.2, 4.2)	37.3, CH	3.26, ddd (8.7, 5.6, 3.1)
8	78.3, CH	4.51, ddd (4.2, 4.2, 1.7)	77.6, CH	4.81, brddd (5.6, 3.2, 2.8)
9	41.3, CH_2	2.12, dd (15.5, 1.7)<br>1.44, dd (15.5, 4.2)	42.8, CH_2	2.11, dd (14.8, 3.2)<br>1.52, dd (14.8, 2.8)
10	34.7, C	-	33.1, C	-
11	42.6, CH	3.22, ddd (9.3, 6.4, 4.5)	41.3, CH	3.30, ddd (9.5, 8.7, 3.9)
12	178.0, C	-	177.9, C	-
13	38.4, CH_2	2.97, dd (18.7, 4.5)<br>2.63, dd (18.7, 9.3)	40.0, CH_2	3.00, dd (18.7, 3.9)<br>2.66, dd (18.7, 9.5)
14	17.6, CH_3	0.74, s	28.7, CH_3	1.22, s
15	106.2, CH_2	4.73, d (1.2)<br>4.39, d (1.2)	23.2, CH_3	1.13, d (7.6)
16	205.9, C	-	206.1, C	-
17	30.0, CH_3	2.20, s	30.0, CH_3	2.25, s

[a] 400/100 MHz. [b] 500/125 MHz.

Table S4. ^{1}H NMR and ^{13}C NMR (500/125 MHz) Data for derivatives **10** and **11**.

	**10**[a]		**11**[b]	
Position	δ_C, type	δ_H (*J* in Hz)	δ_C, type	δ_H (*J* in Hz)
1	42.2, CH_2	1.54, m<br>1.22, m	43.4, CH_2	1.50, m<br>1.07, m
2	22.7, CH_2	1.58, m	17.7, CH_2	1.80, ddd (13.3, 3.8, 3.8)<br>1.33, m
3	36.8, CH_2	2.33, brd (12.5)<br>2.00, brddd (12.5, 12.2, 6.6)	33.8, CH_2	1.52, m
4	149.4, C	-	39.1, CH	2.43, m
5	46.6, CH	1.80, brd (12.3)	148.5, C	-
6	21.8, CH_2	1.67, ddd (13.2, 5.9, 2.2)<br>1.07, ddd (13.2, 12.3, 12.3)	122.0, CH	4.95, brs
7	40.0, CH	2.48, dddd (12.3, 10.5, 5.9, 4.6)	35.3, CH	2.92, m
8	78.0, CH	4.47, m	64.5, CH	4.27, m
9	41.6, CH_2	2.17, brd (15.3)<br>1.46, dd (15.3, 4.1)	45.9, CH_2	1.75, dd (14.2, 3.7)<br>1.45, dd (14.2, 2.6)
10	34.9, C	-	34.2, C	-
11	43.7, CH	2.76, m	41.4, CH	2.28, brddd (5.3, 5.0, 3.6)
12	178.7, C	-	174.4, C	-
13	30.7, CH_2	2.25, brd (14.9)<br>1.85, dd (14.9, 8.8)	34.3, CH_2	2.21, ddd (13.4, 5.0, 0.9)<br>1.70, dd (13.4, 5.3)
14	17.8, CH_3	0.79, s	28.3, CH_3	1.42, s
15	106.5, CH_2	4.78, brs<br>4.47, brs	22.8, CH_3	1.19, d (7.6)
16	101.1, C	-	97.8, C	-
17	21.3, CH_3	1.32, s	23.2, CH_3	1.11, s
18[c]	48.5, CH_3	3.20, s	51.2, CH_3	3.48, s
19[c]	48.3, CH_3	3.20, s	47.6, CH_3	3.04, s

[a] $CDCl_3$. [b] C_6D_6. [c] Interchangeable signals.

Table S5. ^{1}H NMR and ^{13}C NMR (500/125 MHz) Data for derivatives **15** and **16**, $CDCl_3$.

	**15**		**16**	
Position	δ_C, type	δ_H (J in Hz)	δ_C, type	δ_H (J in Hz)
1	44.2, CH	3.09, brddd (9.2, 7.6, 6.1)	44.1, CH	3.08, m
2	39.6, CH_2	2.15, ddd (13.6, 6.5, 6.1)<br>1.88, ddd (13.6, 7.6, 5.5)	39.5, CH_2	2.13, ddd (13.6, 6.3, 6.3)<br>1.88, ddd (13.6, 7.7, 5.2)
3	74.4, CH	4.67, brddd (6.5, 5.5, 1.4)	74.3, CH	4.66, brddd (6.3, 5.2, 1.3)
4	154.4, C	-	154.1, C	-
5	49.9, CH	2.97, brddd (9.6, 9.2, 1.6)	49.5, CH	3.00, brddd (11.0, 9.6, 1.7)
6	83.7, CH	4.12, dd (9.6, 9.6)	83.9, CH	4.23, dd (9.6, 9.6)
7	49.8, CH	2.26, ddd (11.7, 9.6, 3.6)	51.7, CH	1.97, m
8	25.6, CH_2	1.92, dddd (13.2, 4.8, 4.6, 3.6)<br>1.63, dddd (13.2, 12.0, 11.7, 4.8)	25.5, CH_2	1.74, m
9	36.5, CH_2	2.51, ddd (12.6, 4.8, 4.8)<br>1.99, ddd (12.6, 12.0, 4.6)	36.2, CH_2	2.54, ddd (12.9, 4.6, 4.6)<br>1.96, m
10	149.1, C	-	148.7, C	-
11	75.4, C	-	76.3, C	-
12	176.6, C	-	175.8, C	-
13	42.5, CH_2	2.30, d (14.8)<br>2.19, d (14.8)	44.4, CH_2	2.81, d (16.7)<br>2.64, d (16.7)
14	112.5, CH_2	4.91, s<br>4.77, s	112.8, CH_2	4.92, s<br>4.79, s
15	113.2, CH_2	5.46, brdd (2.0, 1.4)<br>5.36, brdd (2.0, 1.4)	113.4, CH_2	5.46, brdd (2.0, 1.3)<br>5.37, brdd (2.0, 1.3)
16	108.6, C	-	209.9, C	-
17	25.7, CH_3	1.42, s	32.1, CH_3	2.31, s
18[a]	64.3, CH_2	3.98, m	-	-
19[a]	64.0, CH_2	3.98, m	-	-

[a] Interchangeable signals.

Table S6. ^{1}H NMR and ^{13}C NMR comparison for synthetized and isolated **1**.

	Synthetized **1**		Isolated **1**	
Position	δ_C, type	δ_H (J in Hz)	δ_C, type	δ_H (J in Hz)
1	42.1, CH_2	1.53, m<br>1.24, m	42.2, CH_2	1.53, m<br>1.23, m
2	22.6, CH_2	1.58, m	22.8, CH_2	1.60, m<br>1.26, m
3	36.7, CH_2	2.32, ddd (12.8, 4.2, 2.2)<br>2.00, ddd (12.8, 12.6, 5.8)	36.9, CH_2	2.34, m<br>2.00, m
4	149.2, C	-	149.4, C	-
5	46.5, CH	1.80, brdd (12.3, 1.4)	46.7, CH	1.81, d (12.4)
6	21.1, CH_2	1.42, ddd (13.1, 6.1, 2.5)<br>1.05, ddd (13.1, 12.8, 12.3)	21.2, CH_2	1.42, m<br>1.06, q (12.4)
7	46.8, CH	2.39, ddd (12.8, 6.1, 4.1)	47.0, CH	2.38, m
8	77.5, CH	5.04, ddd (4.2, 4.1, 2.0)	77.7, CH	5.04, m
9	41.2, CH_2	2.20, dd (15.6, 2.0)<br>1.46, dd (15.6, 4.2)	41.4, CH_2	2.20, dd (15.6, 2.6)<br>1.46, dd (15.6, 4.5)
10	34.5, C	-	34.7, C	-
11	79.5, C	-	79.7, C	-
12	175.4, C	-	175.5, C	-
13	42.0, CH_2	3.00, d (17.4)<br>2.64, d (17.4)	42.2, CH_2	3.02, d (17.5)<br>2.64, d (17.5)
14	17.8, CH_3	0.78, s	18.0, CH_3	0.78, s
15	106.3, CH_2	4.79, brdd (1.5, 1.4)<br>4.43, brdd (1.5, 1.4)	106.5, CH_2	4.79, s<br>4.43, s
16	210.3, C	-	210.5, C	-
17	31.9, CH_3	2.34, s	32.0, CH_3	2.34, s

Table S7. ^{1}H NMR and ^{13}C NMR comparison for synthetized and isolated **2**.

	Synthetized **2**		Isolated **2**	
Position	δ_C, type	δ_H (J in Hz)	δ_C, type	δ_H (J in Hz)
1	42.1, CH_2	1.59, m<br>1.12, m	42.3, CH_2	1.62, m<br>1.15, m
2	16.7, CH_2	1.82, ddddd (13.7, 12.9, 12.6, 4.9, 3.4)<br>1.43, dddd (13.7, 7.2, 3.5, 3.4)	16.9, CH_2	1.80, m<br>1.40, m
3	32.8, CH_2	1.56, m	32.9, CH_2	1.56, m
4	38.6, CH	2.46, m	38.7, CH	2.46, m
5	152.6, C	-	152.8, C	-
6	113.5, CH	4.94, d (3.5)	113.7, CH	4.94, d (3.5)
7	46.9, CH	3.01, dd (5.5, 3.5)	47.1, CH	3.01, dd (5.4, 3.5)
8	77.1, CH	5.13, brddd (5.5, 3.3, 2.7)	77.2, CH	5.13, dt (5.4, 3.0)
9	42.5, CH_2	2.14, dd (14.9, 3.3)<br>1.51, dd (14.9, 2.7)	42.7, CH_2	2.14, dd (14.9, 3.0)<br>1.51, dd (14.9, 3.0)
10	33.0, C	-	33.1, C	-
11	79.0, C	-	79.2, C	-
12	175.3, C	-	175.5, C	-
13	43.4, CH_2	2.95, d (17.5)<br>2.65, d (17.5)	43.6, CH_2	2.95, d (17.5)<br>2.65, d (17.5)
14	28.6, CH_3	1.21, s	28.8, CH_3	1.22, s
15	23.0, CH_3	1.13, d (7.5)	23.2, CH_3	1.13, d (7.7)
16	210.3, C	-	210.4, C	-
17	31.8, CH_3	2.33, s	32.0, CH_3	2.33, s

Table S8. ^{1}H NMR and ^{13}C NMR comparison for synthetized and isolated **3**.

	Synthetized **3**		Isolated **3**	
Position	δ_C, type	δ_H (J in Hz)	δ_C, type	δ_H (J in Hz)
1	44.2, CH	2.87, ddd (8.9, 8.1, 7.9)	44.3, CH	2.88, q (8.4)
2	36.2, CH_2	2.44, ddd (13.9, 7.9, 7.9)<br>1.76, m	36.4, CH_2	2.44, m<br>1.76, m
3	74.3, CH	5.55, brdddd (7.9, 6.7, 2.0. 2.0)	75.5, CH	5.56, m
4	148.0, C	-	148.4, C	-
5	50.1, CH	2.75, brdddd (9.6, 8.9, 2.0, 2.0)	50.3, CH	2.75, t (9.2)
6	82.9, CH	4.36, dd (9.6, 9.6)	83.0, CH	4.37, t (9.2)
7	51.7, CH	1.96, ddd (10.5, 9.6, 5.0)	52.0, CH	1.94, m
8	25.0, CH_2	1.78, m	25.2, CH_2	1.81, m<br>1.64, m
9	34.5, CH_2	2.49, ddd (13.0, 5.2, 5.2)<br>1.99, ddd (13.0, 9.8, 5.3)	34.6, CH_2	2.50, m<br>2.03, m
10	148.2, C	-	148.2, C	-
11	76.2, C	-	76.5, C	-
12	175.8, C	-	176.0, C	-
13	44.3, CH_2	2.80, d (16.8)<br>2.63, d (16.8)	44.4, CH_2	2.80, d (16.7)<br>2.62, d (16.7)
14	113.8, CH_2	4.92, s<br>4.91, s	114.0, CH_2	4.93, s<br>4.91, s
15	114.2, CH_2	5.42, dd (2.0, 2.0)<br>5.29, dd (2.0, 2.0)	114.5, CH_2	5.43, brs<br>5.30, brs
16	209.9, C	-	210.4, C	-
17	32.0, CH_3	2.31, s	32.3, CH_3	2.32, s
1’	172.9, C	-	173.1, C	-
2’	43.6, CH_2	2.23, brdd (7.2, 1.7)	43.8, CH_2	2.23, dd (7.1, 1.7)
3’	25.7, CH	2.12, brsepd (6.6, 0.8)	25.9, CH	2.12, m
4’[a]	22.40, CH_3	0.97, d (6.6)	22.5, CH_3	0.97, d (6.5)
5’[a]	22.37, CH_3	0.97, d (6.6)	22.6, CH_3	0.97, d (6.5)

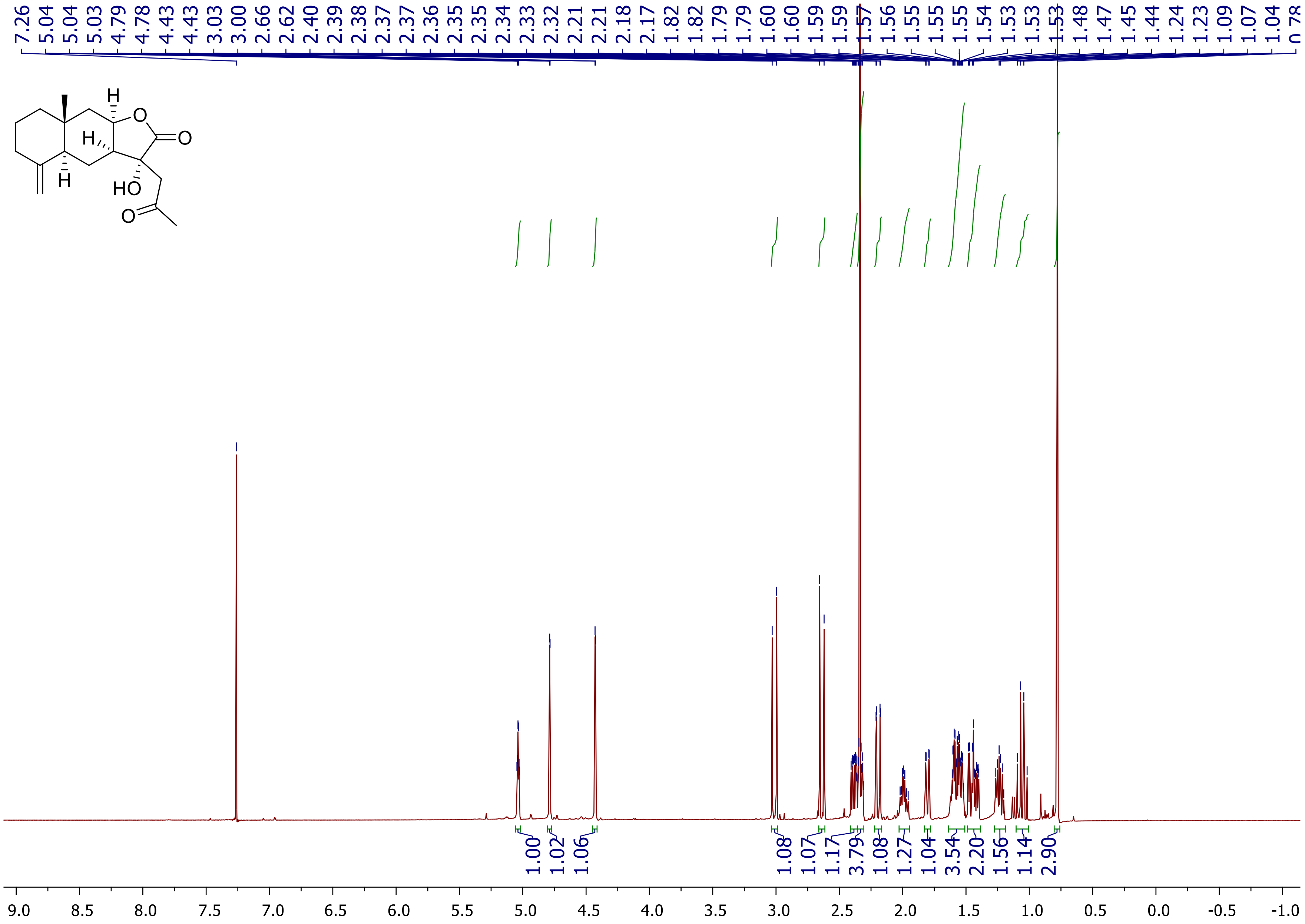

Figure S1. ^{1}H-NMR (500 MHz) spectrum of **1** in $CDCl_3$.

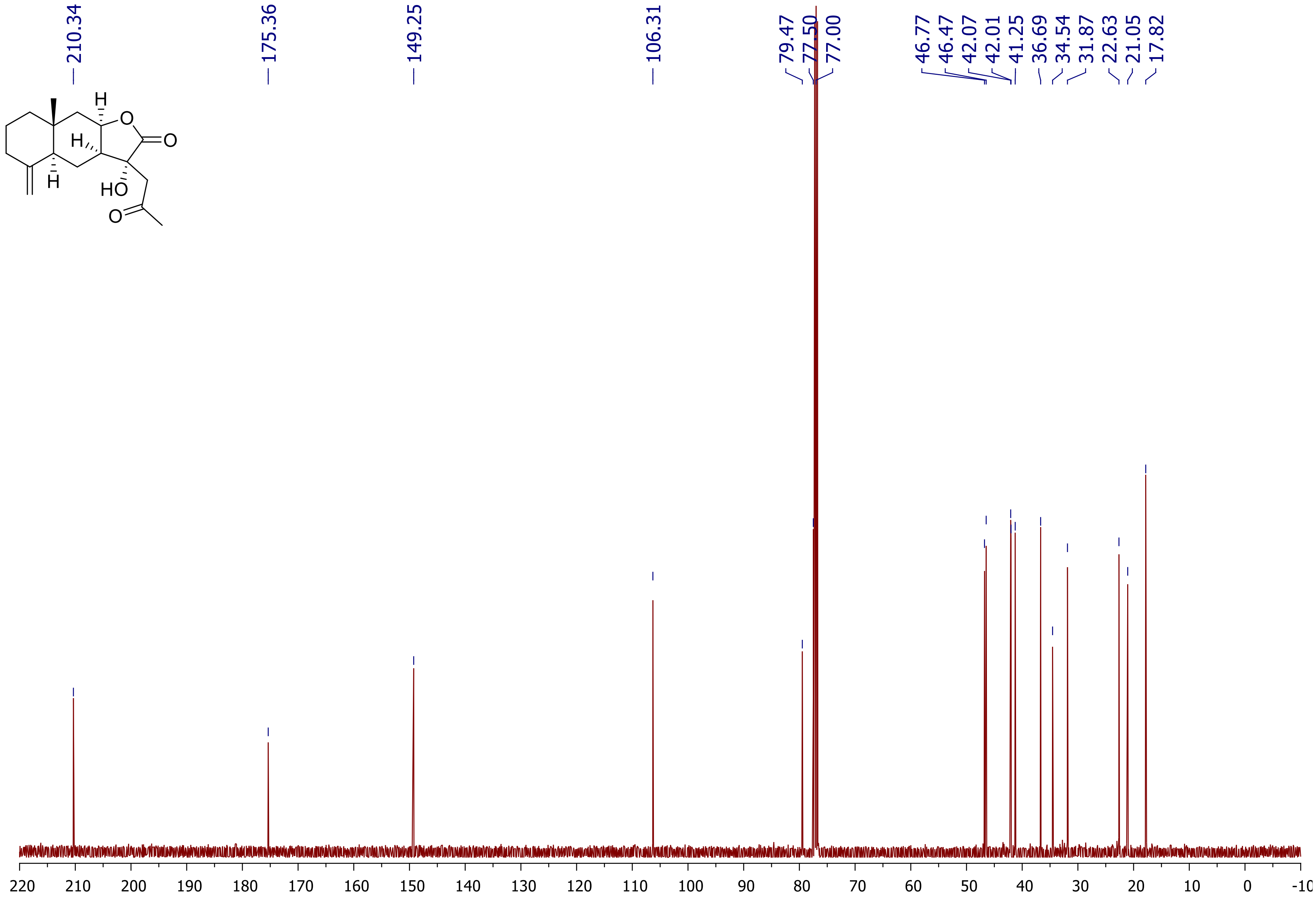

Figure S2. ^{13}C NMR (125 MHz) spectrum of **1** in $CDCl_3$.

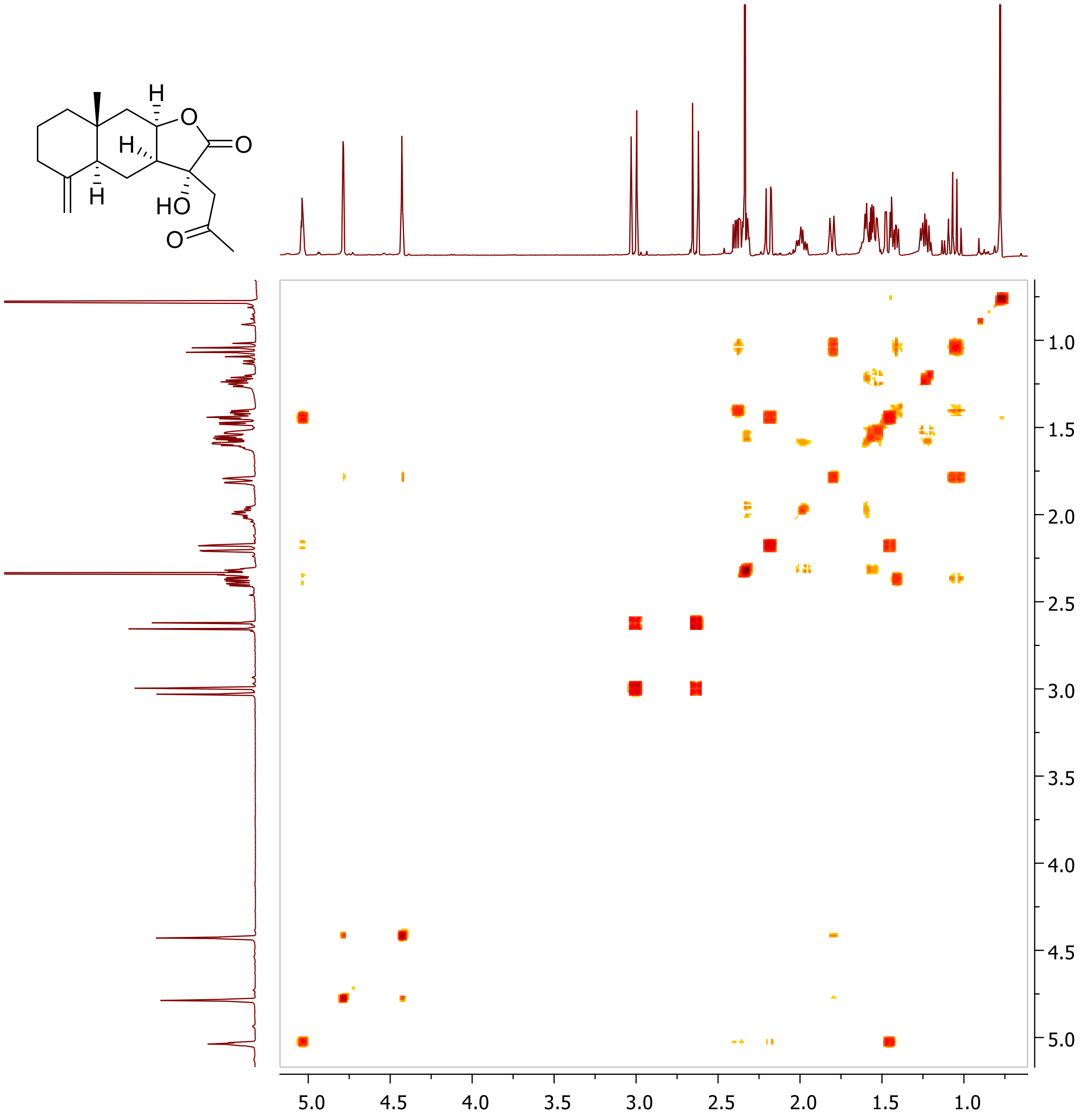

Figure S3. ^{1}H-^{1}H-COSY spectrum of **1** in $CDCl_3$.

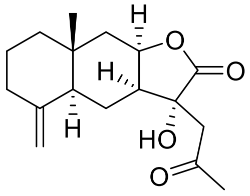

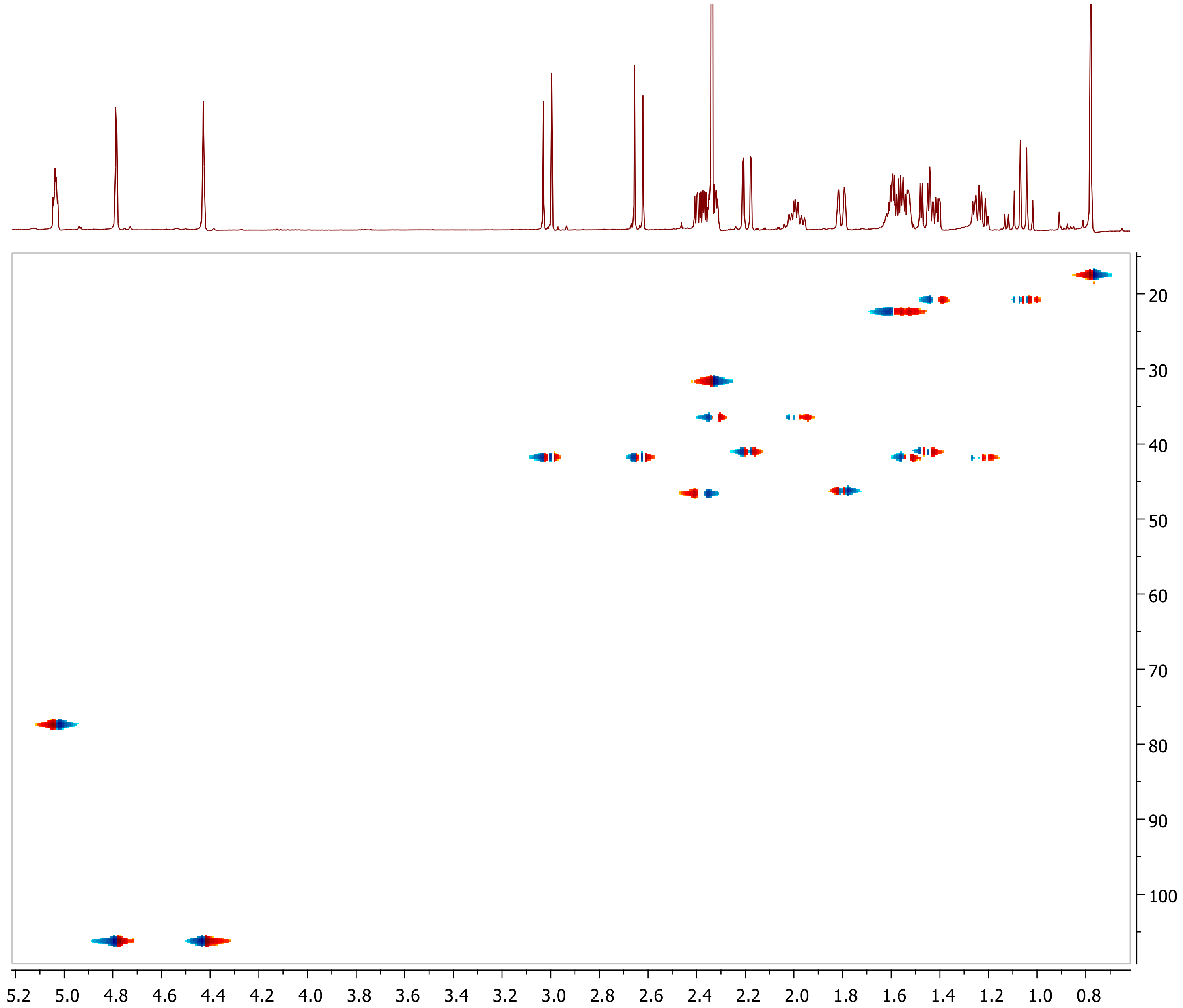

Figure S4. HSQC spectrum of **1** in $CDCl_3$.

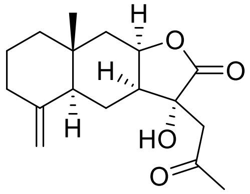

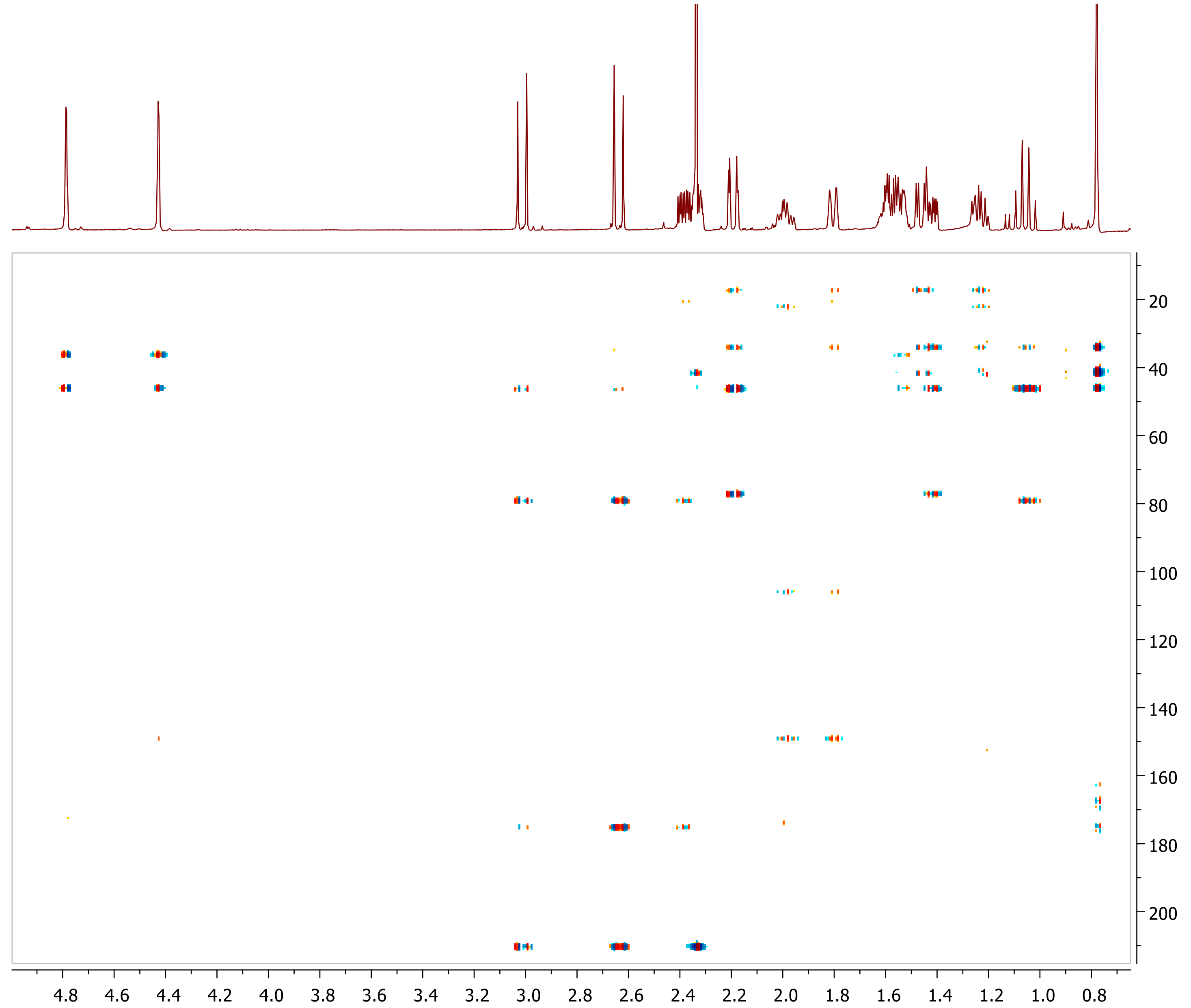

Figure S5. HMBC spectrum of **1** in $CDCl_3$.

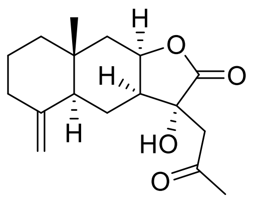

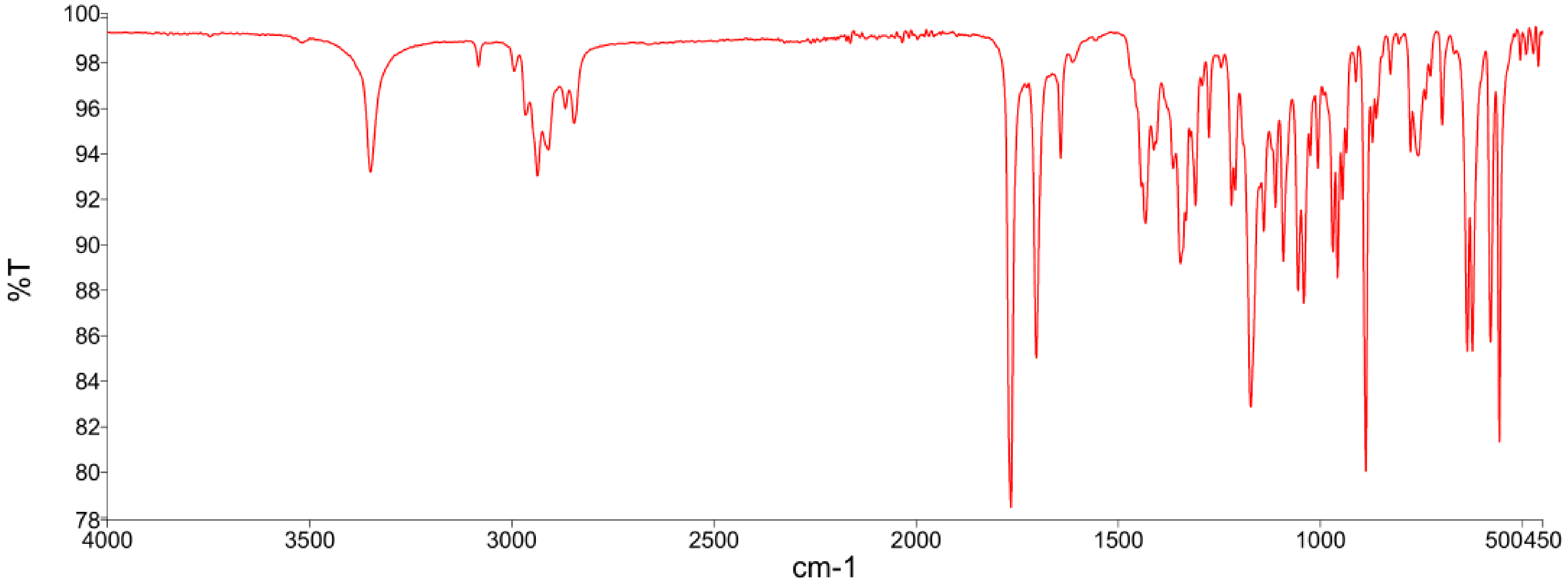

Figure S6. IR spectrum of **1**.

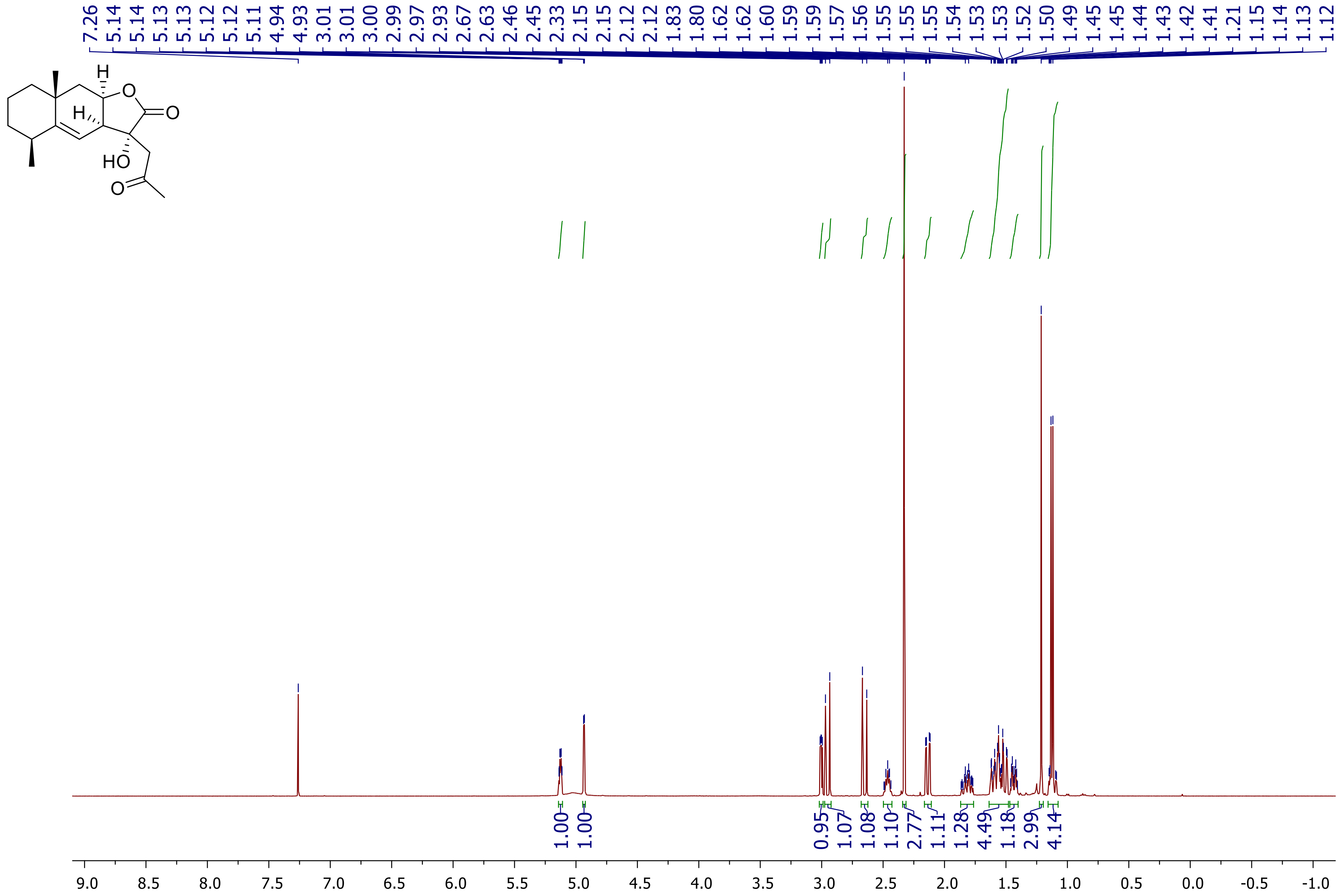

Figure S7. ^{1}H-NMR (500 MHz) spectrum of **2** in $CDCl_3$.

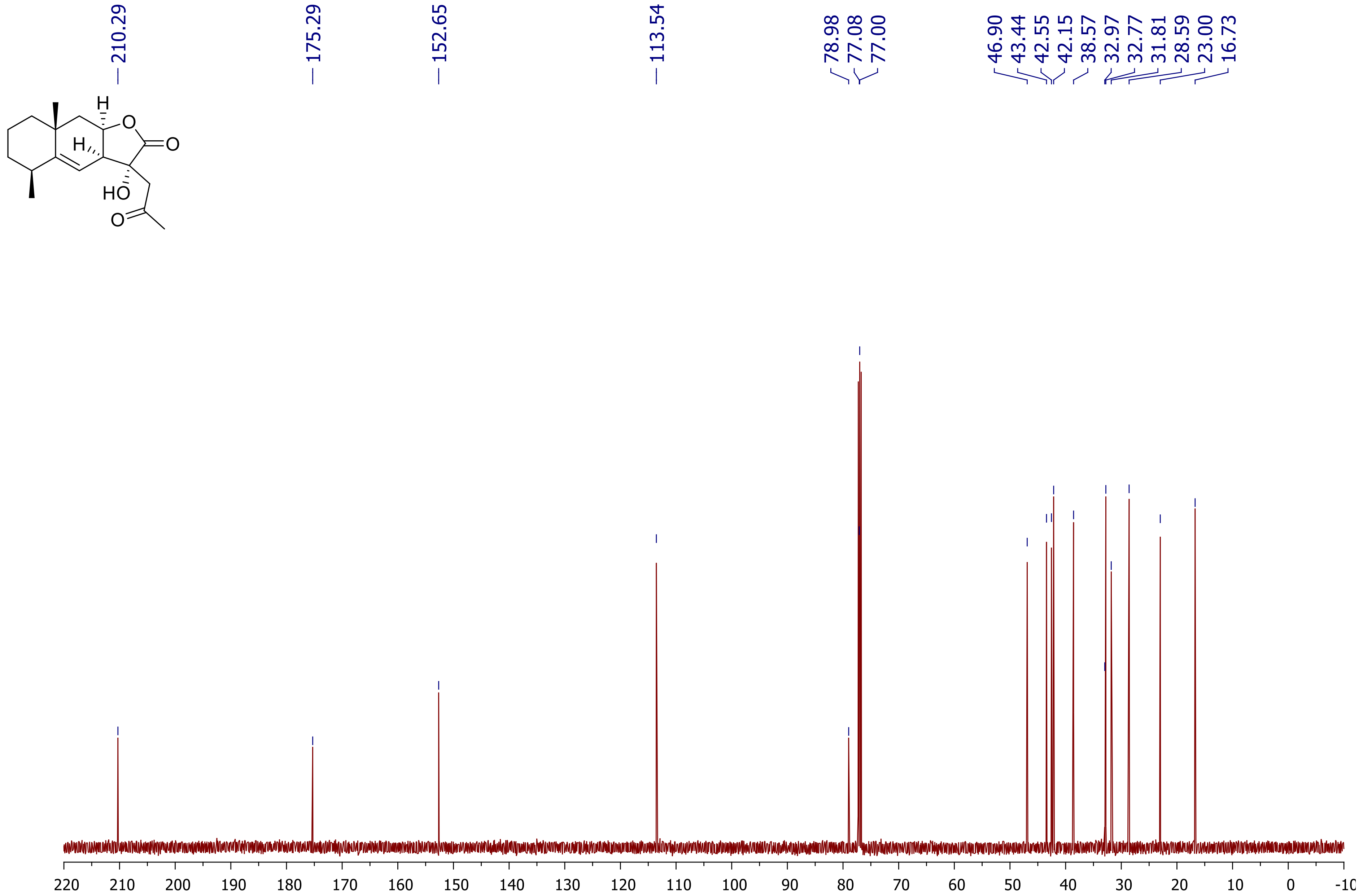

Figure S8. ^{13}C NMR (125 MHz) spectrum of **2** in $CDCl_3$.

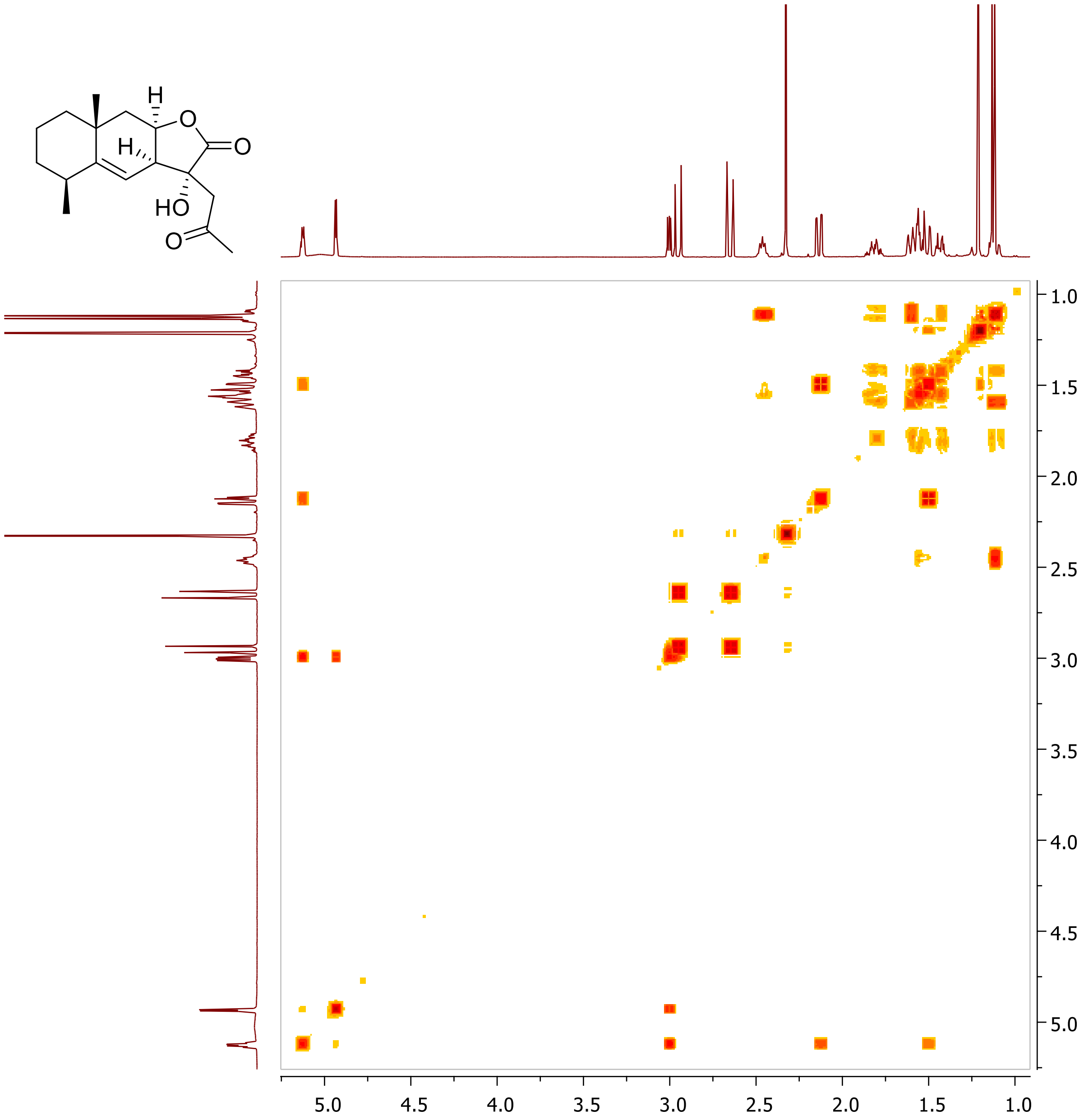

Figure S9. ^{1}H-^{1}H-COSY spectrum of **2** in $CDCl_3$.

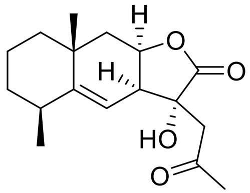

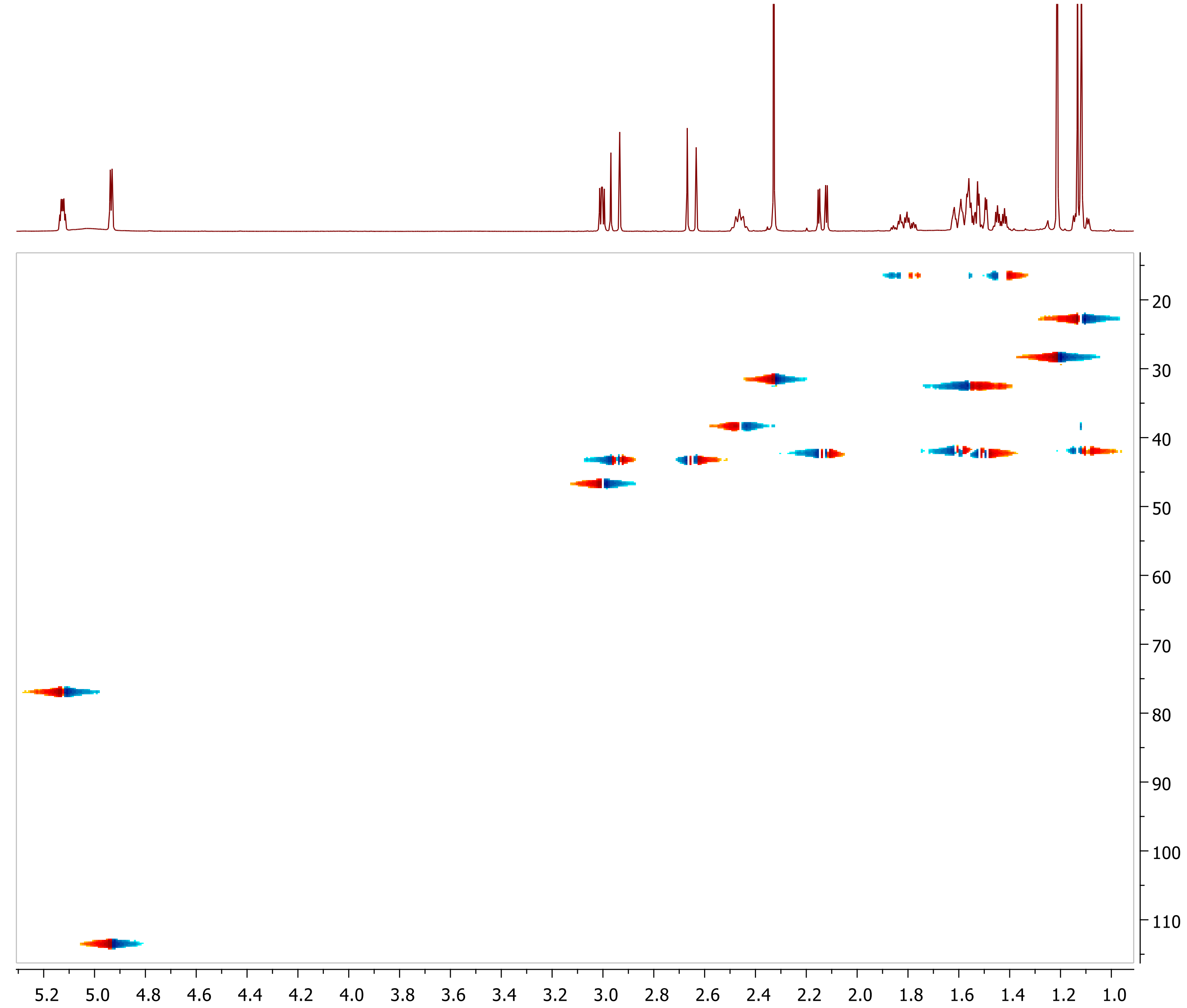

Figure S10. HSQC spectrum of **2** in $CDCl_3$.

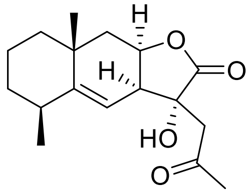

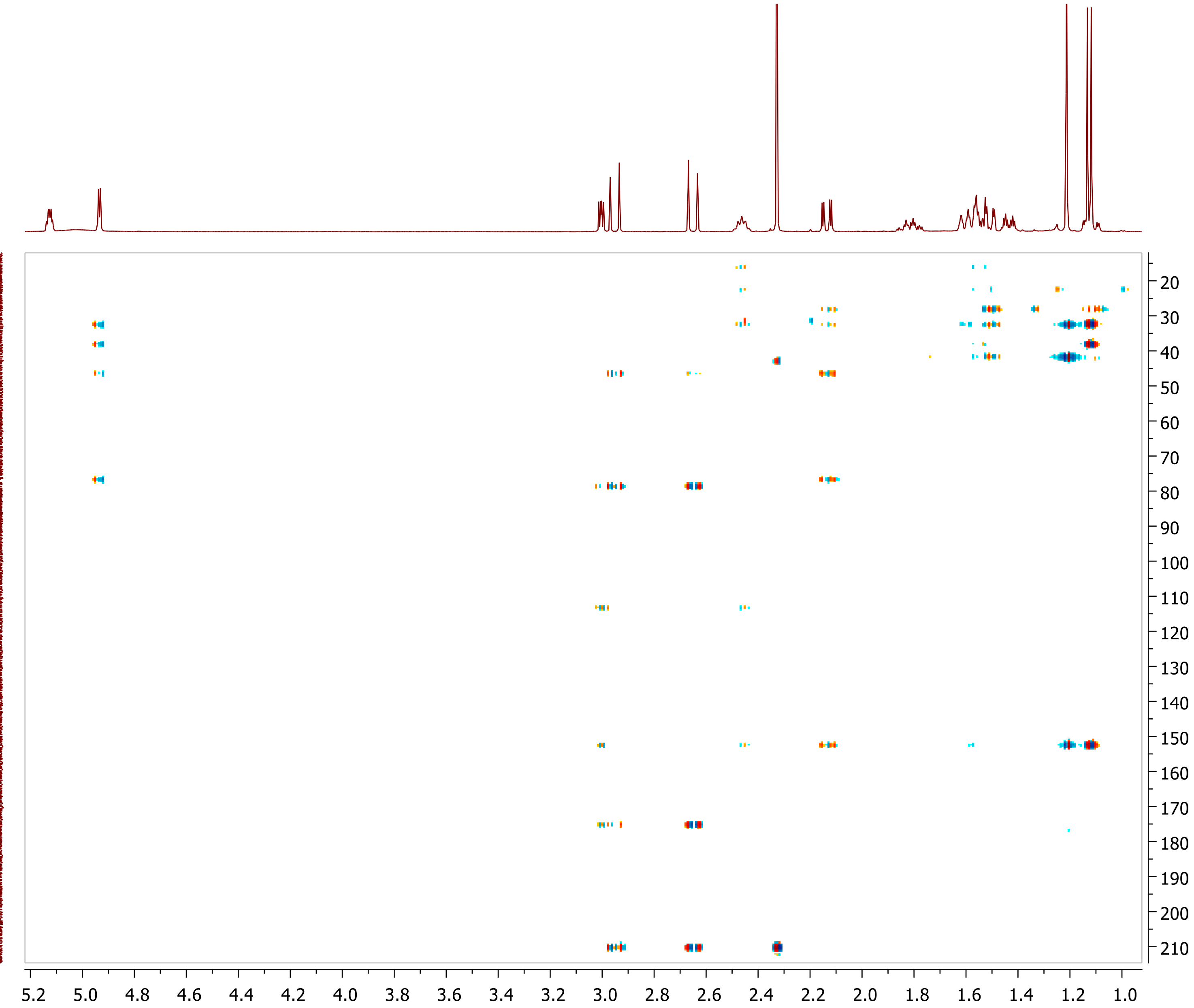

Figure S11. HMBC spectrum of **2** in $CDCl_3$.

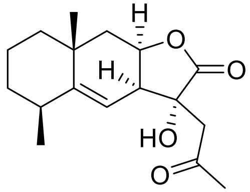

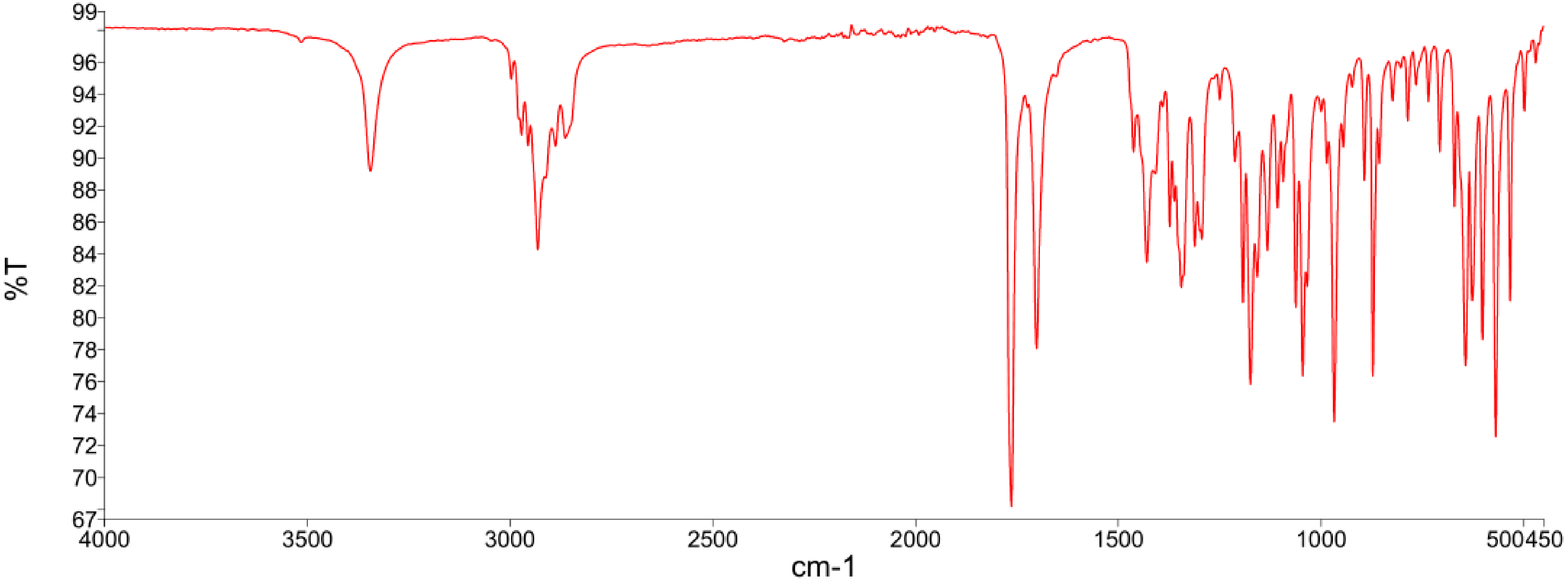

Figure S12. IR spectrum of **2**.

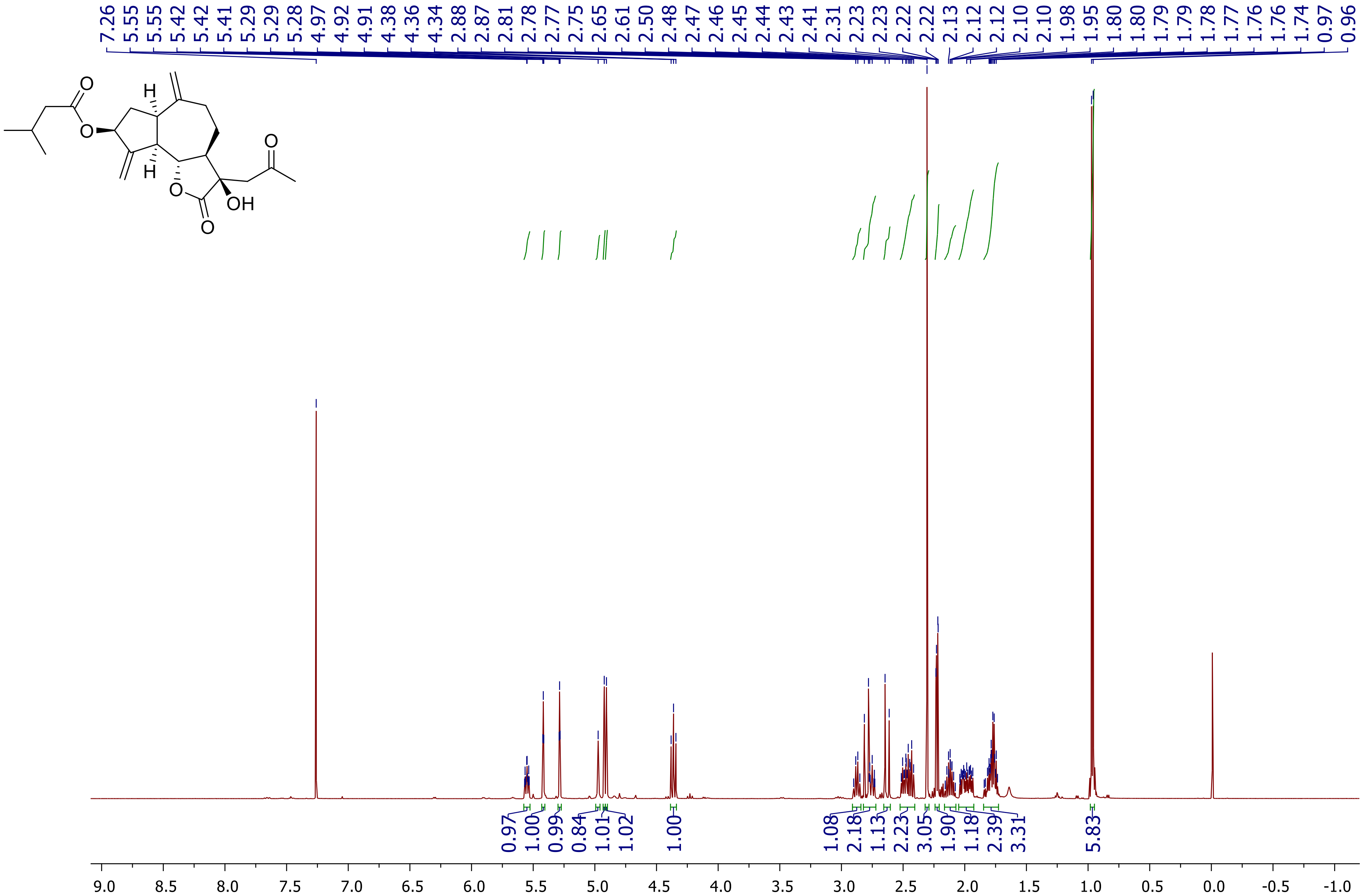

Figure S13. ^{1}H-NMR (500 MHz) spectrum of **3** in $CDCl_3$.

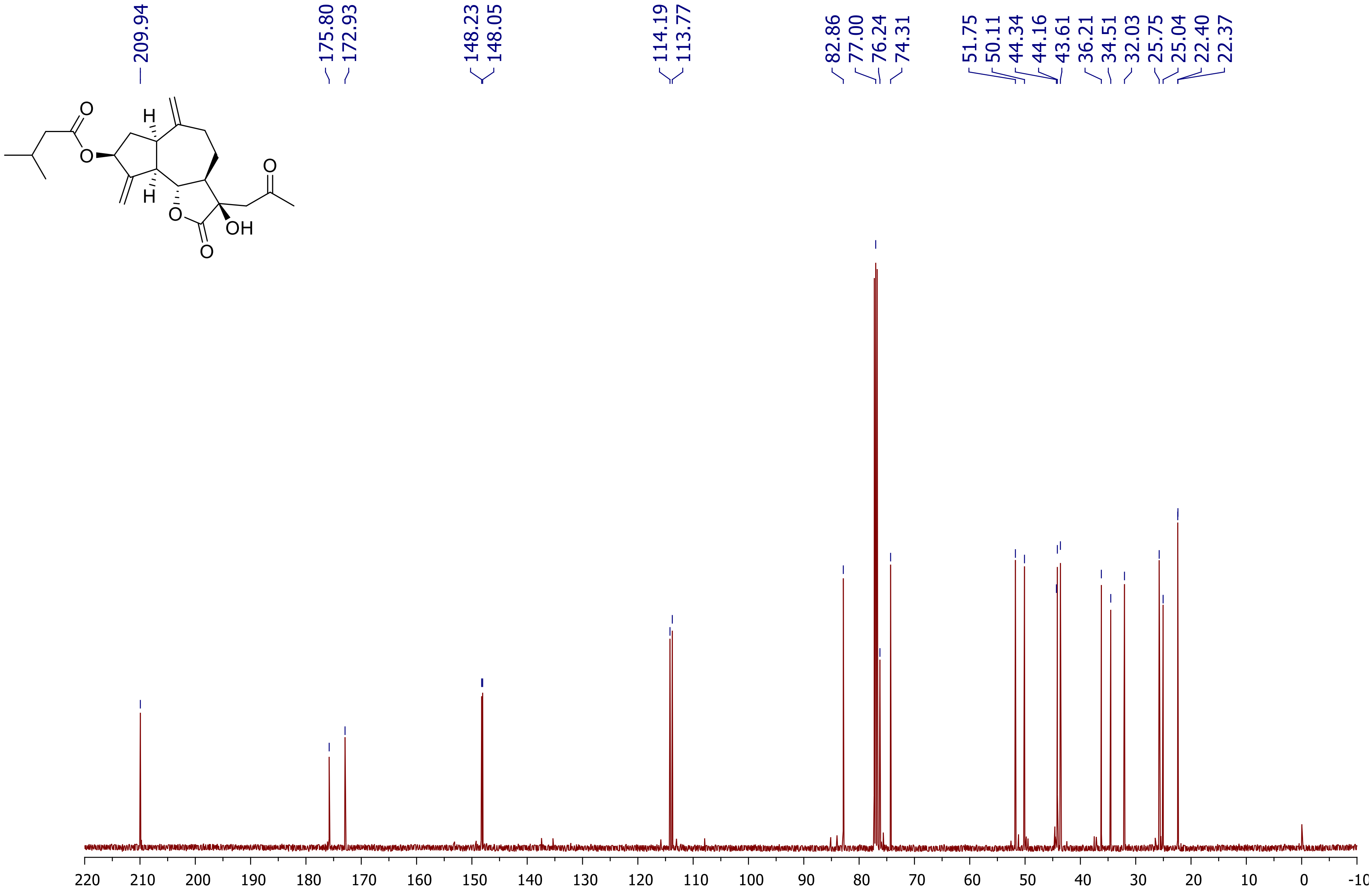

Figure S14. ^{13}C NMR (125 MHz) spectrum of **3** in $CDCl_3$.

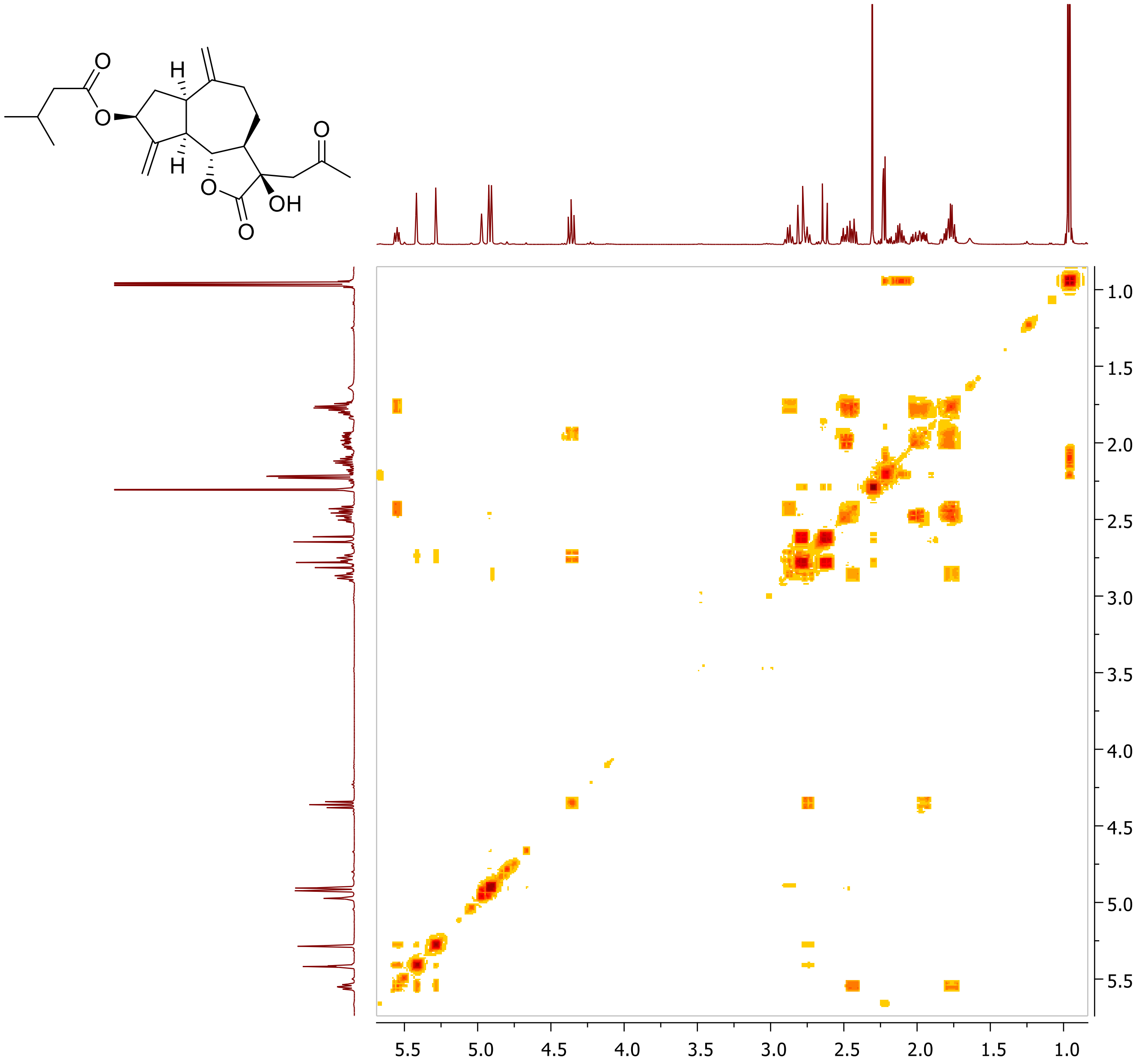

Figure S15. ^{1}H-^{1}H-COSY spectrum of **3** in $CDCl_3$.

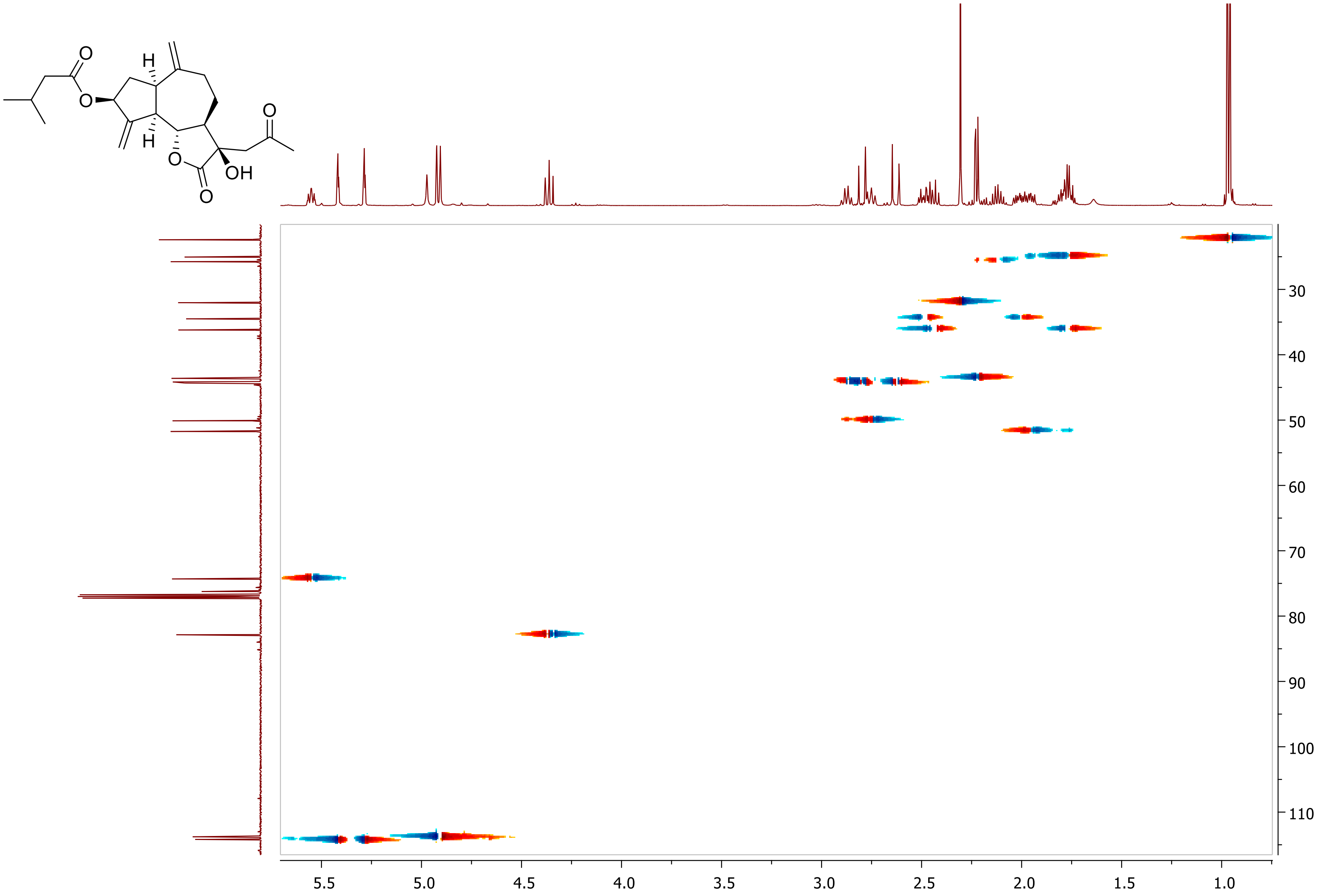

Figure S16. HSQC spectrum of **3** in $CDCl_3$.

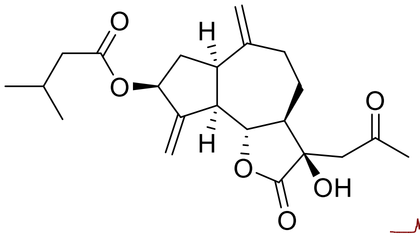

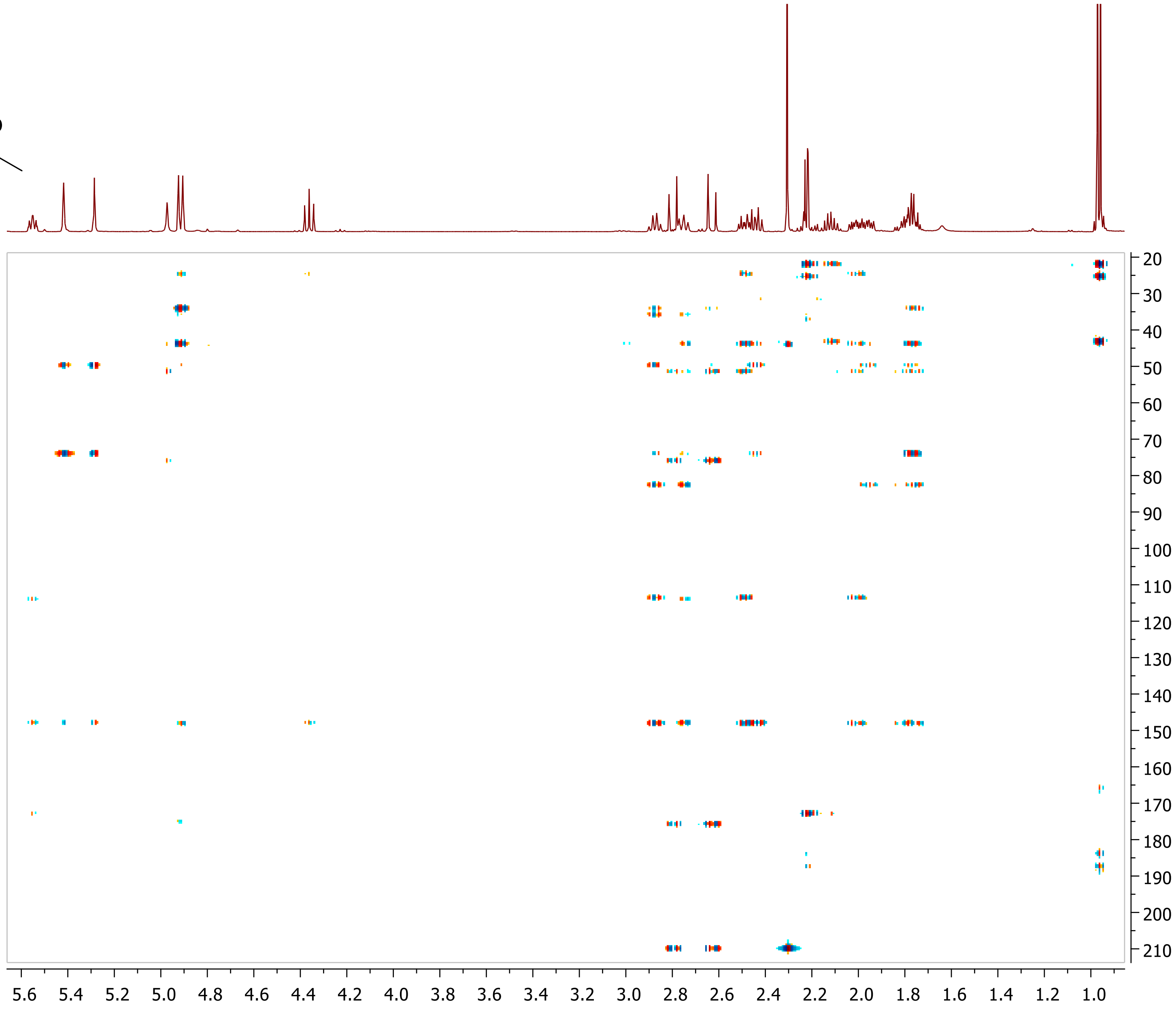

Figure S17. HMBC spectrum of **3** in $CDCl_3$.

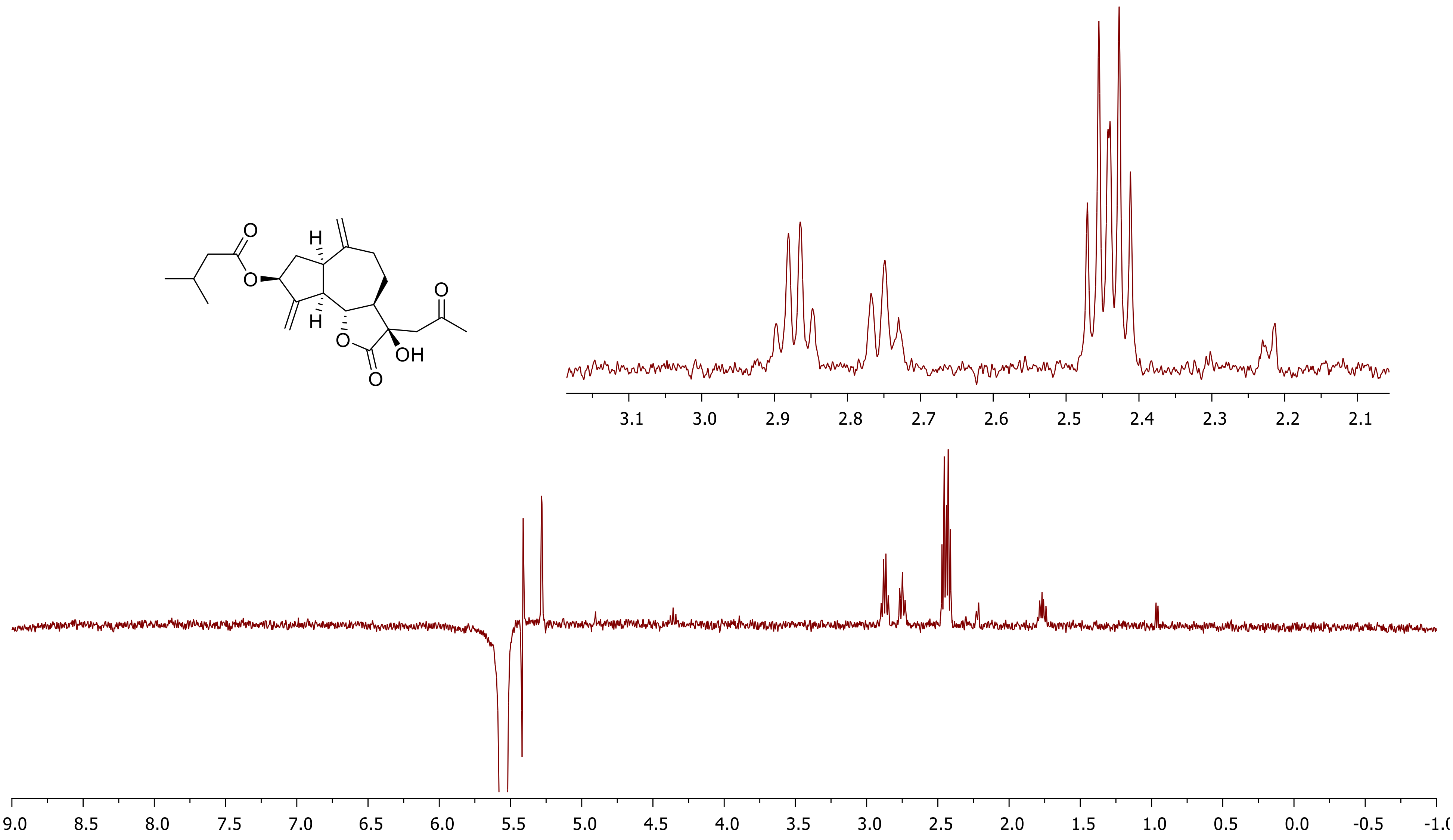

Figure S18. 1D-NOESY spectrum of **3** in $CDCl_3$.

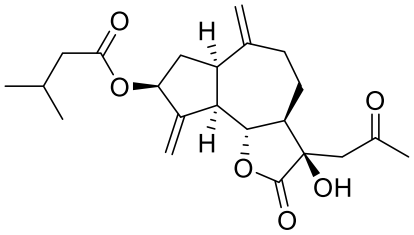

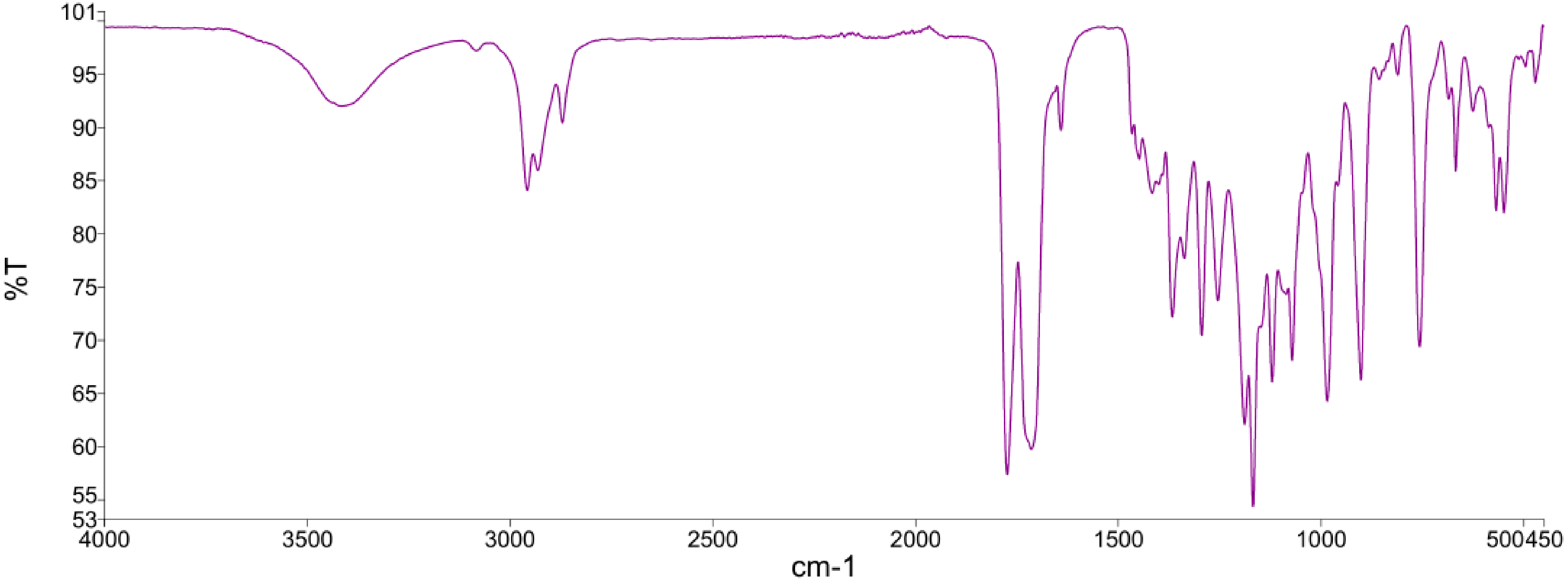

Figure S19. IR spectrum of **3**.

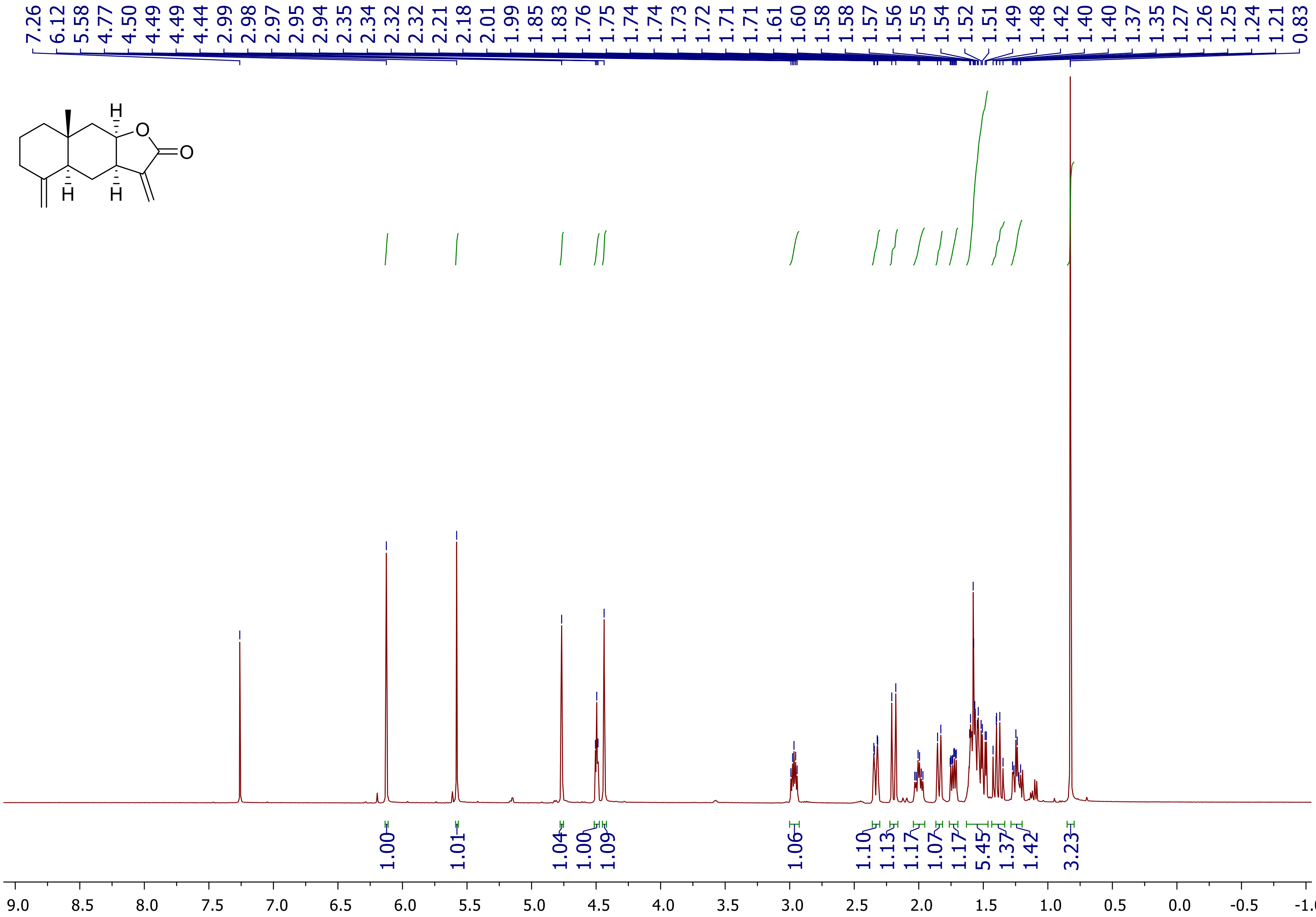

Figure S20. ^{1}H-NMR (500 MHz) spectrum of **4** in $CDCl_3$.

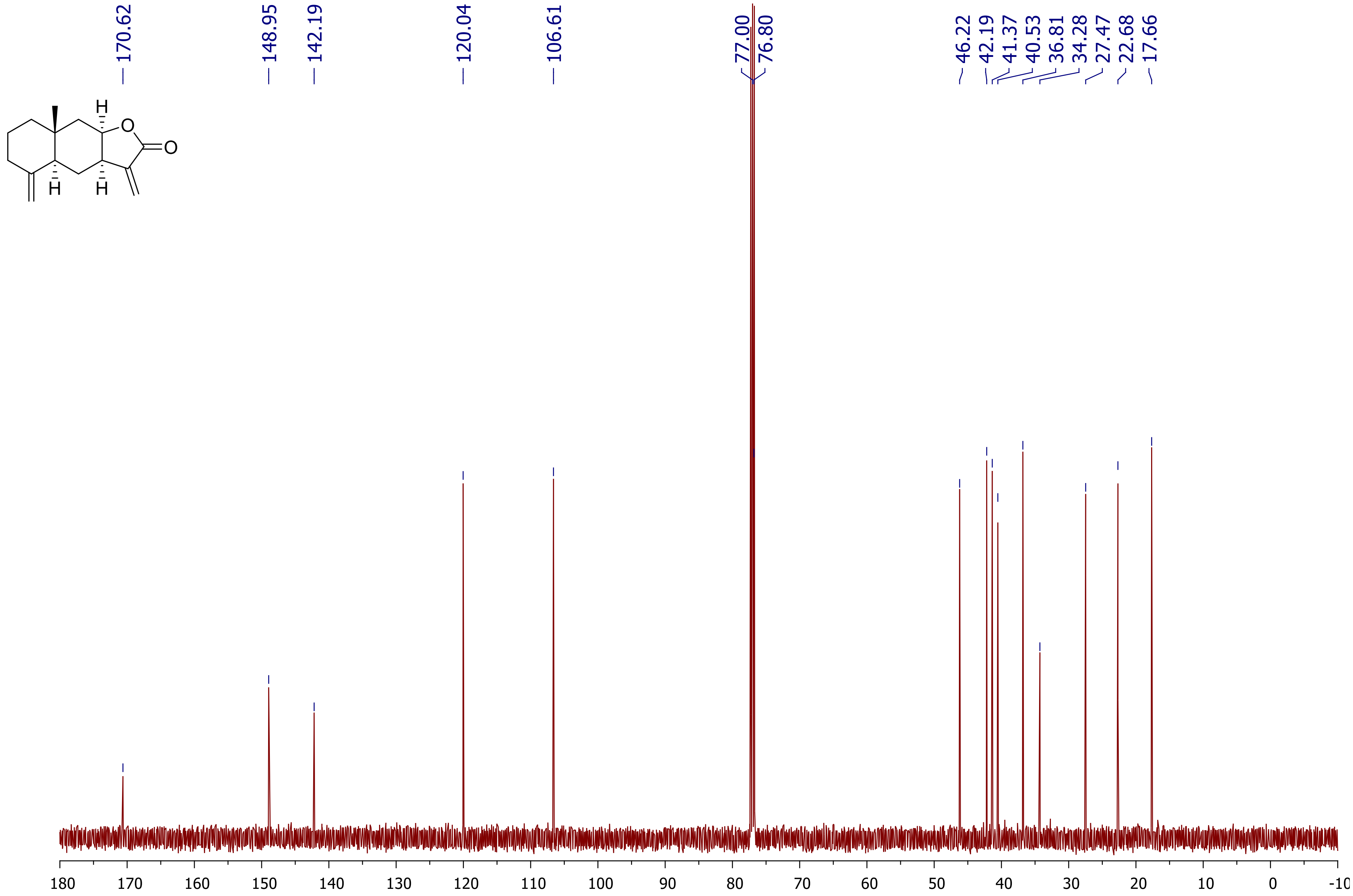

Figure S21. ^{13}C NMR (125 MHz) spectrum of **4** in $CDCl_3$.

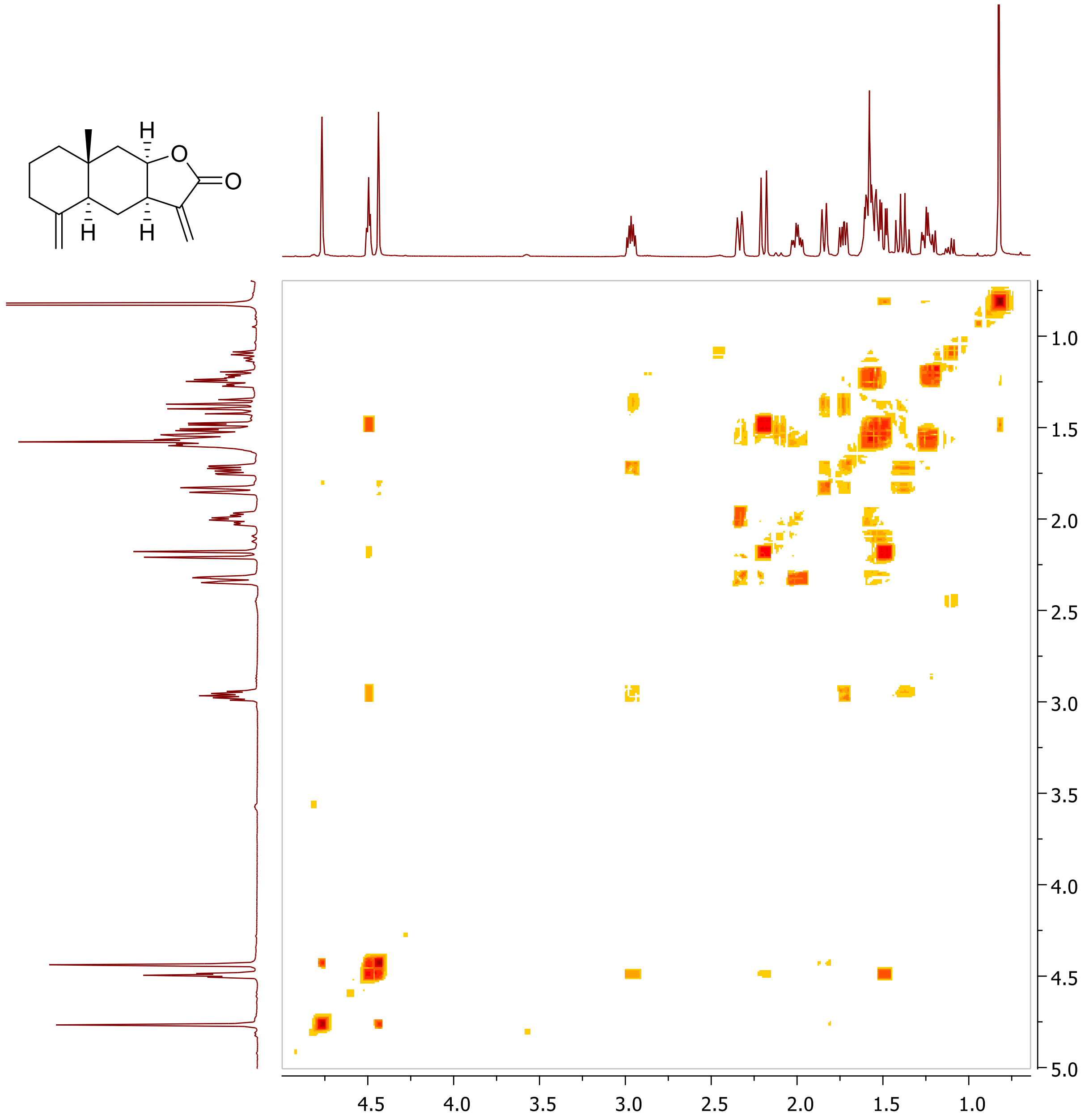

Figure S22. ^{1}H-^{1}H-COSY spectrum of **4** in $CDCl_3$.

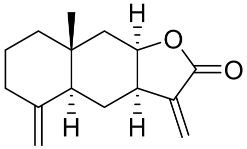

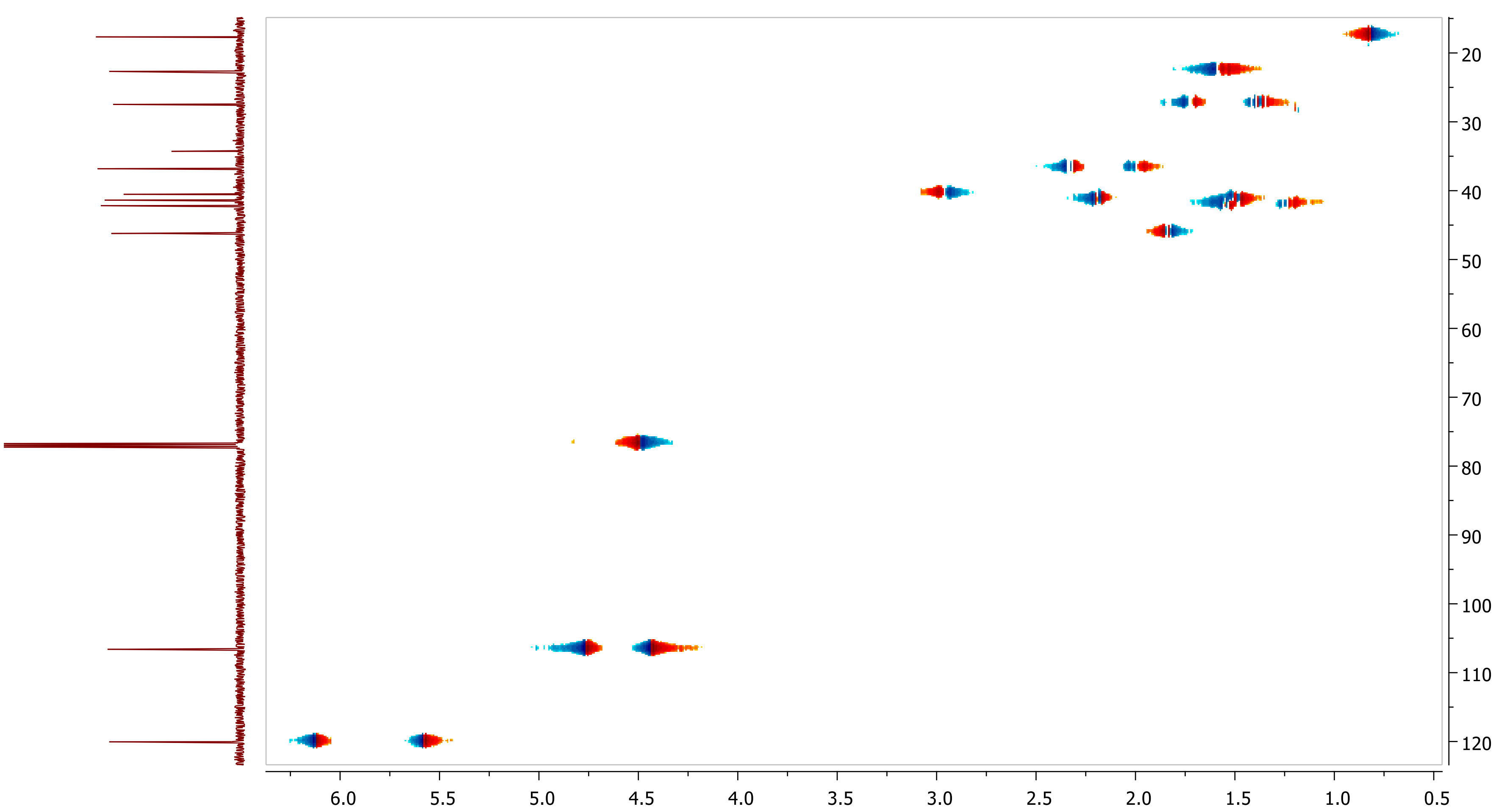

Figure S23. HSQC spectrum of **4** in $CDCl_3$.

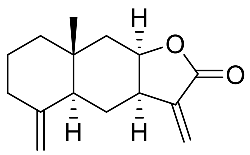

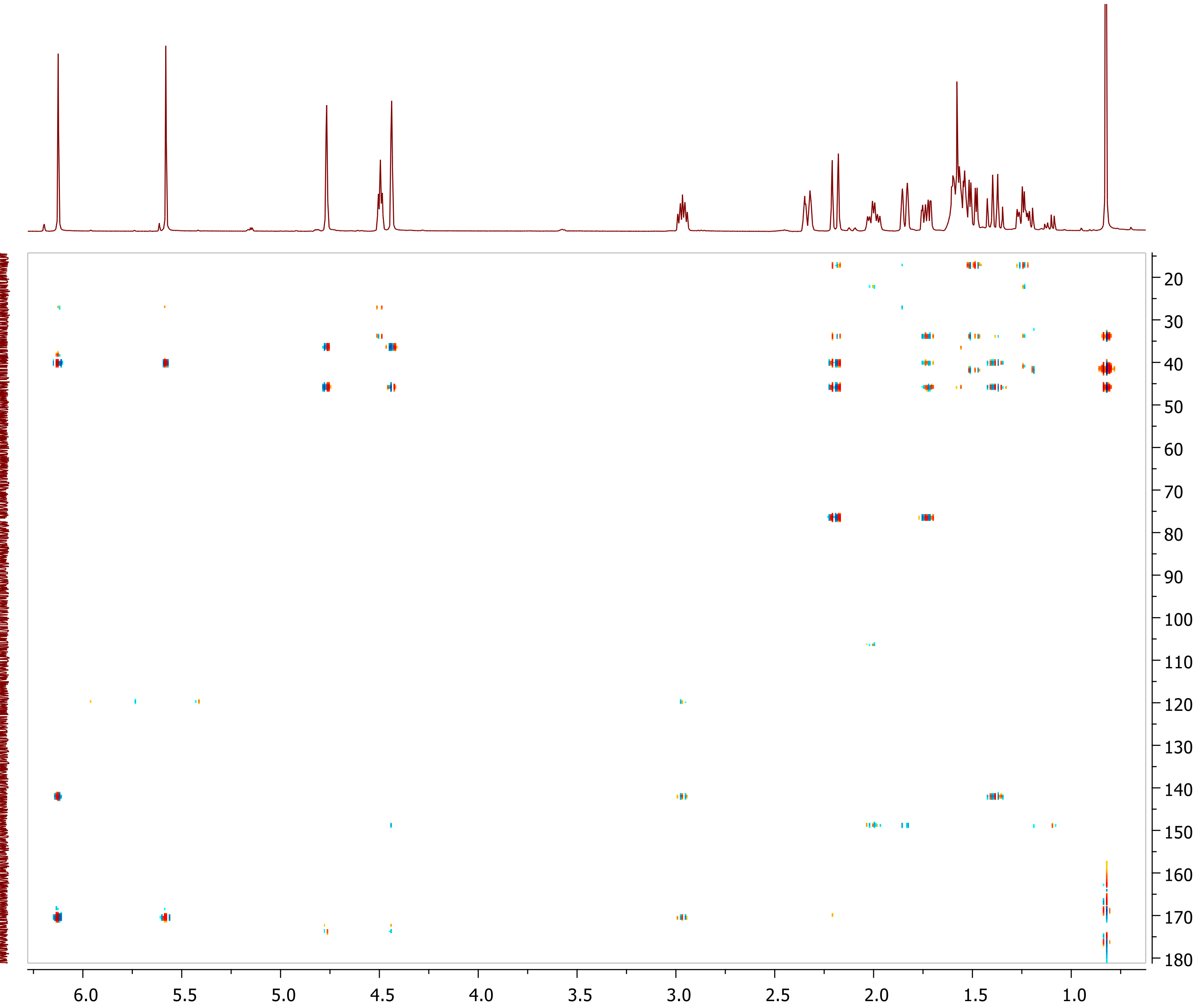

Figure S24. HMBC spectrum of **4** in $CDCl_3$.

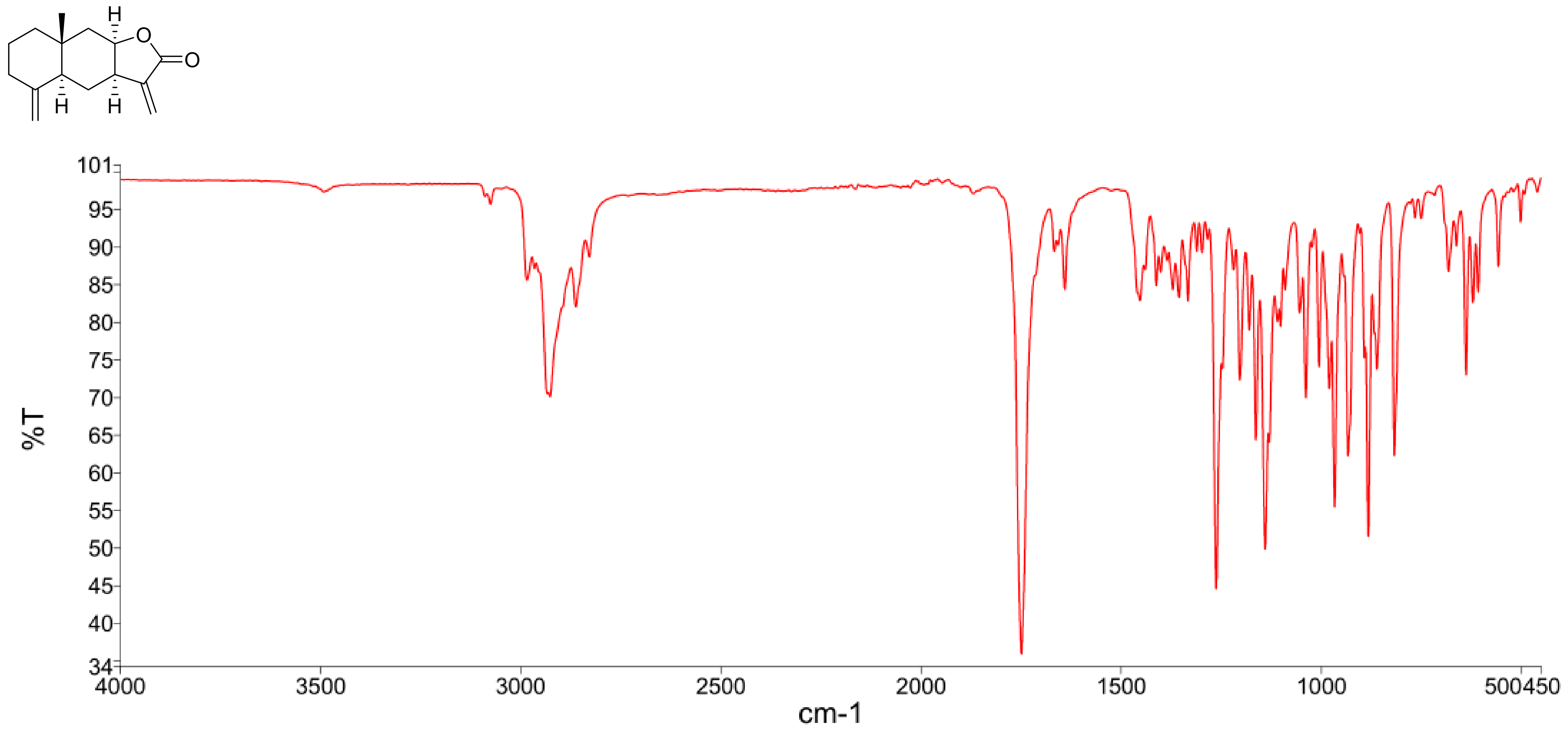

Figure S25. IR spectrum of **4**.

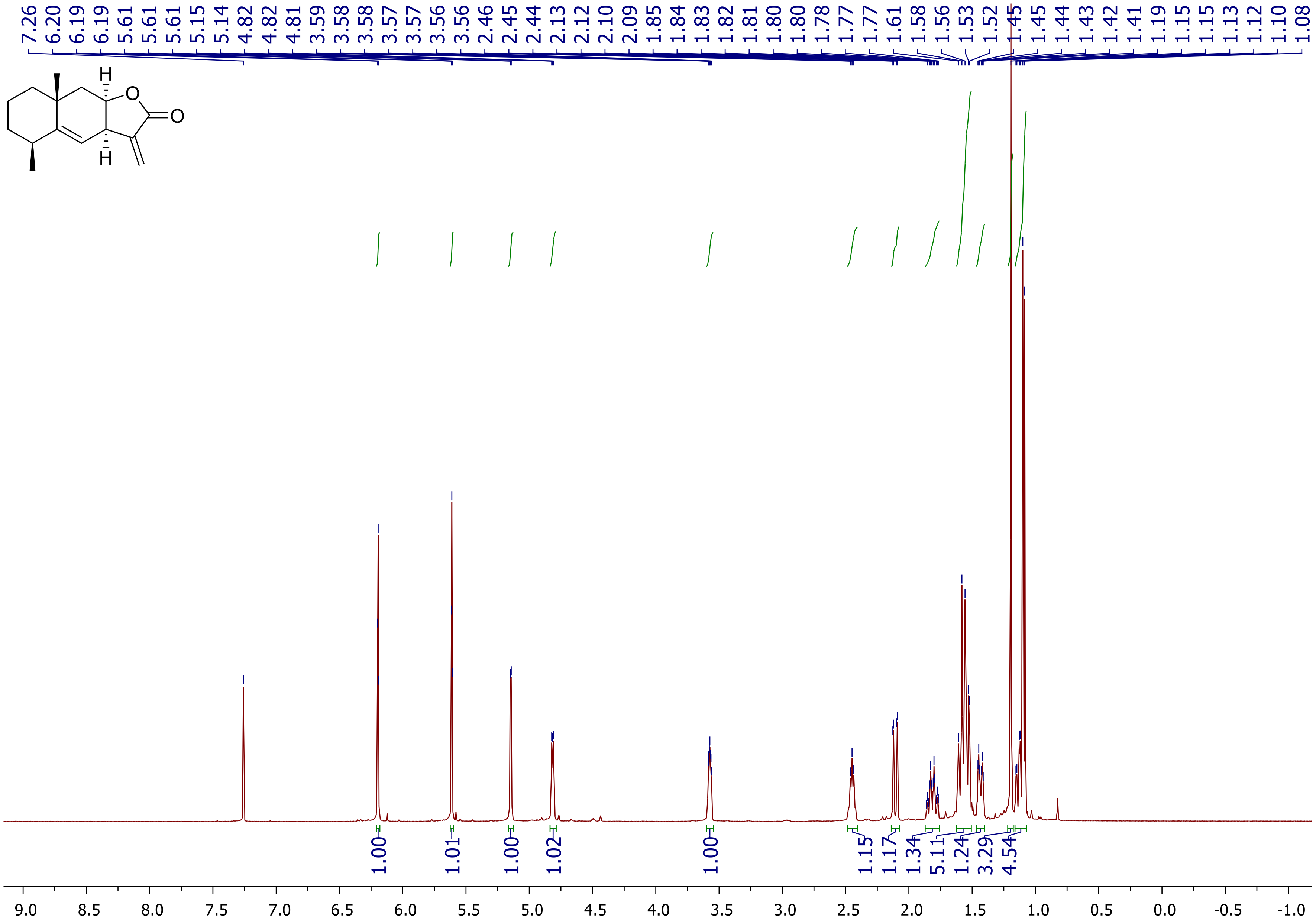

Figure S26. ^{1}H-NMR (500 MHz) spectrum of **5** in $CDCl_3$.

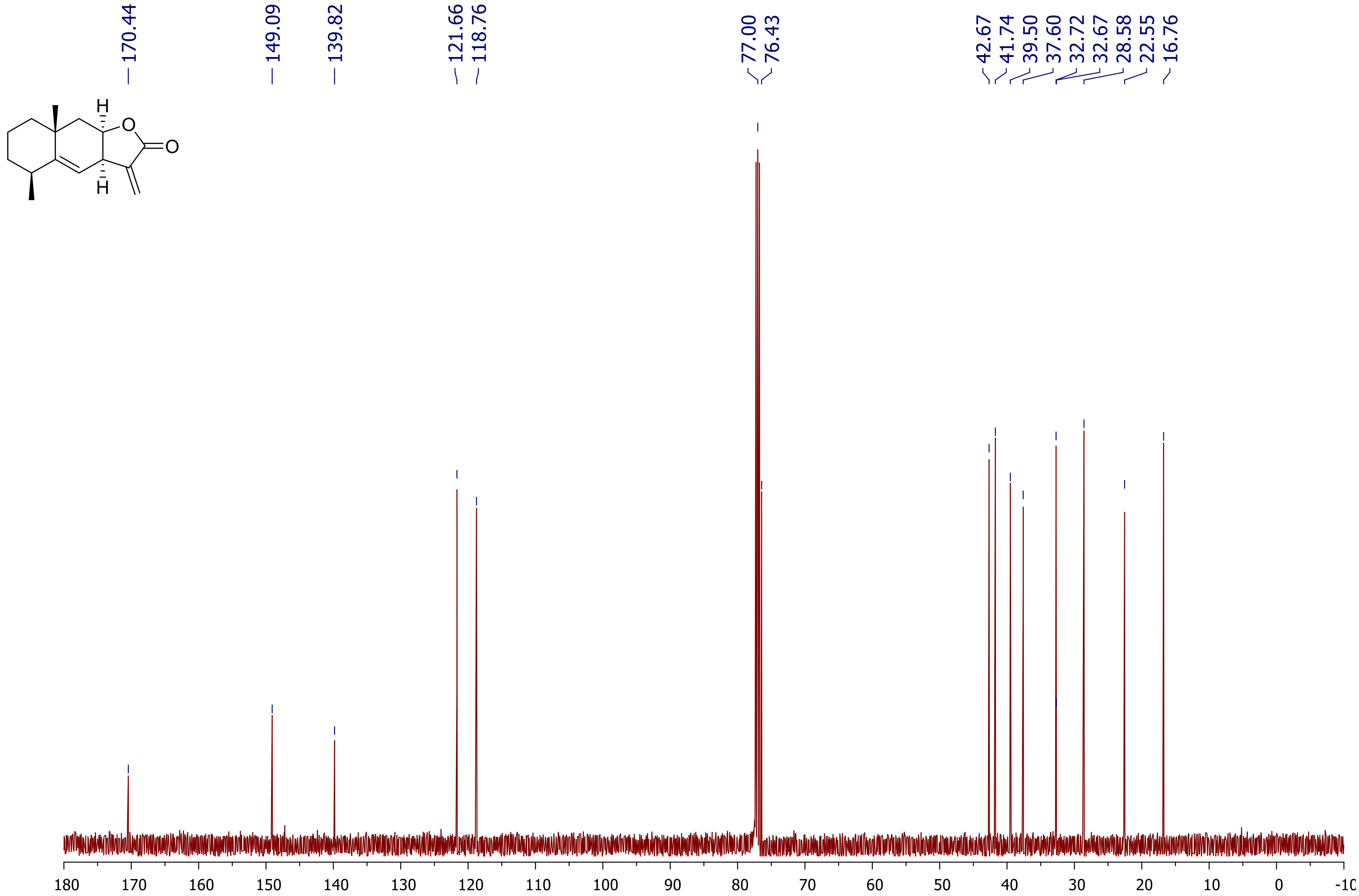

Figure S27. ^{13}C NMR (125 MHz) spectrum of **5** in $CDCl_3$.

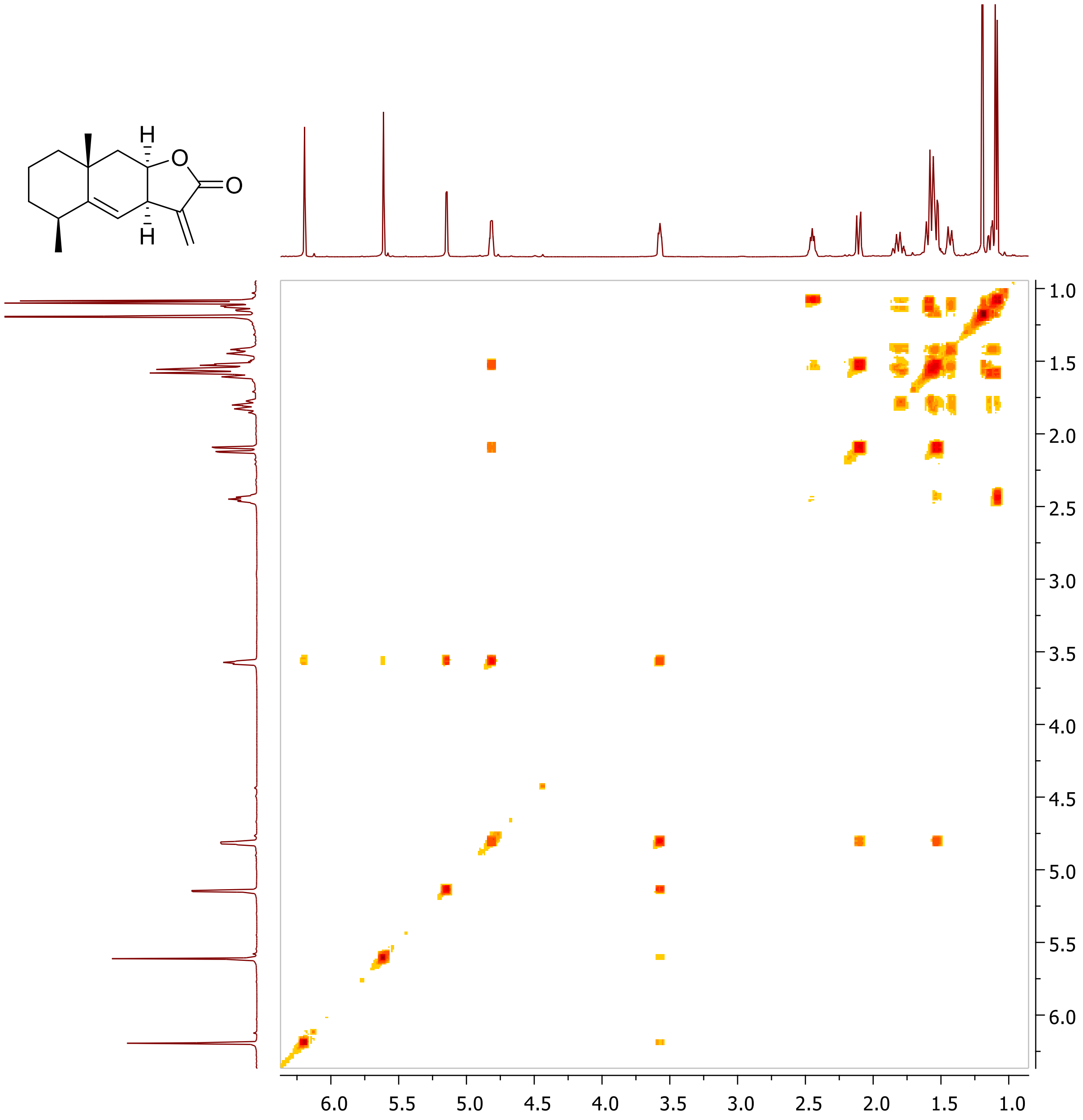

Figure S28. ^{1}H-^{1}H-COSY spectrum of **5** in $CDCl_3$.

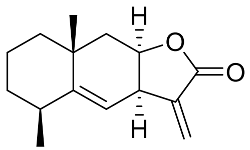

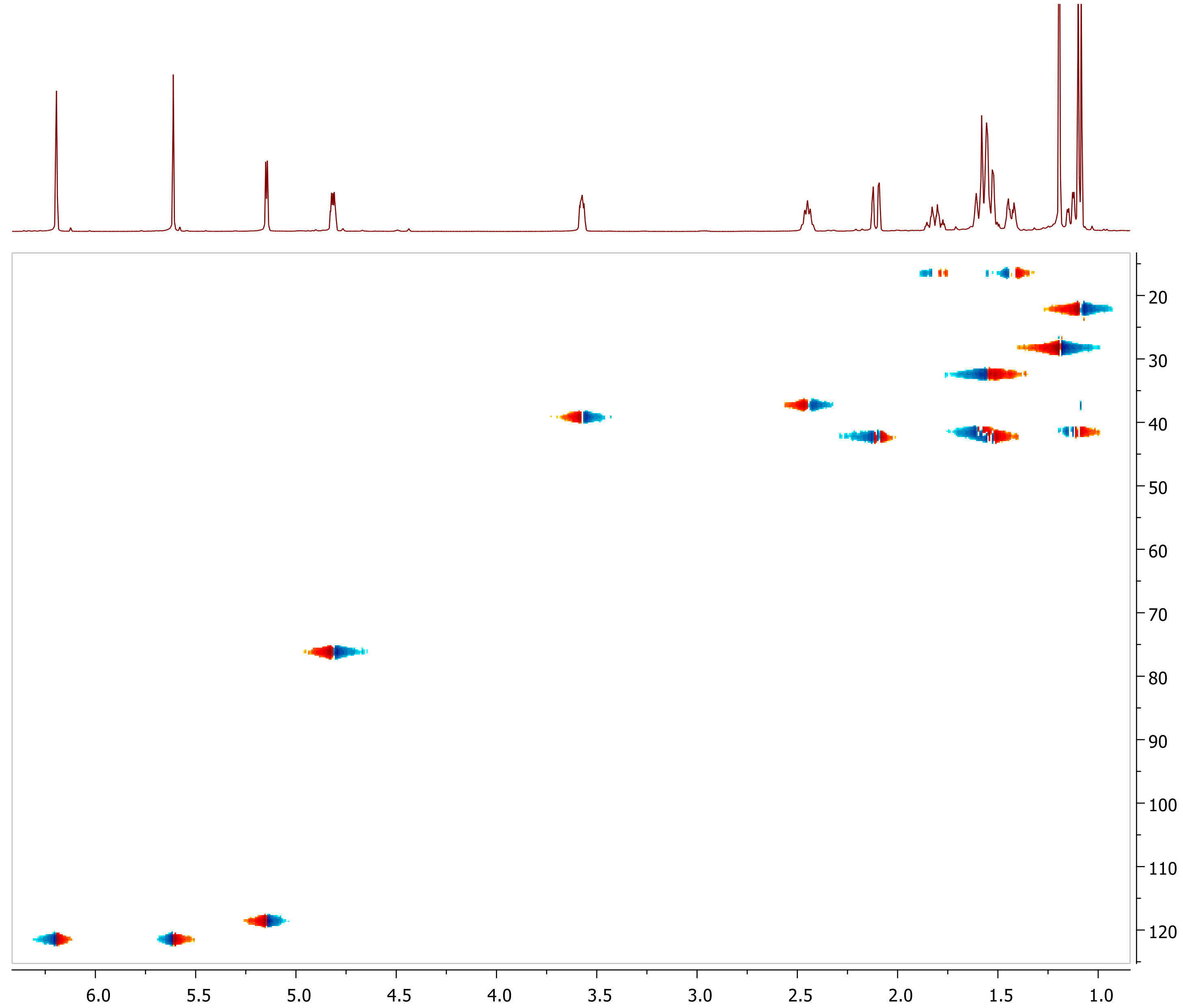

Figure S29. HSQC spectrum of **5** in $CDCl_3$.

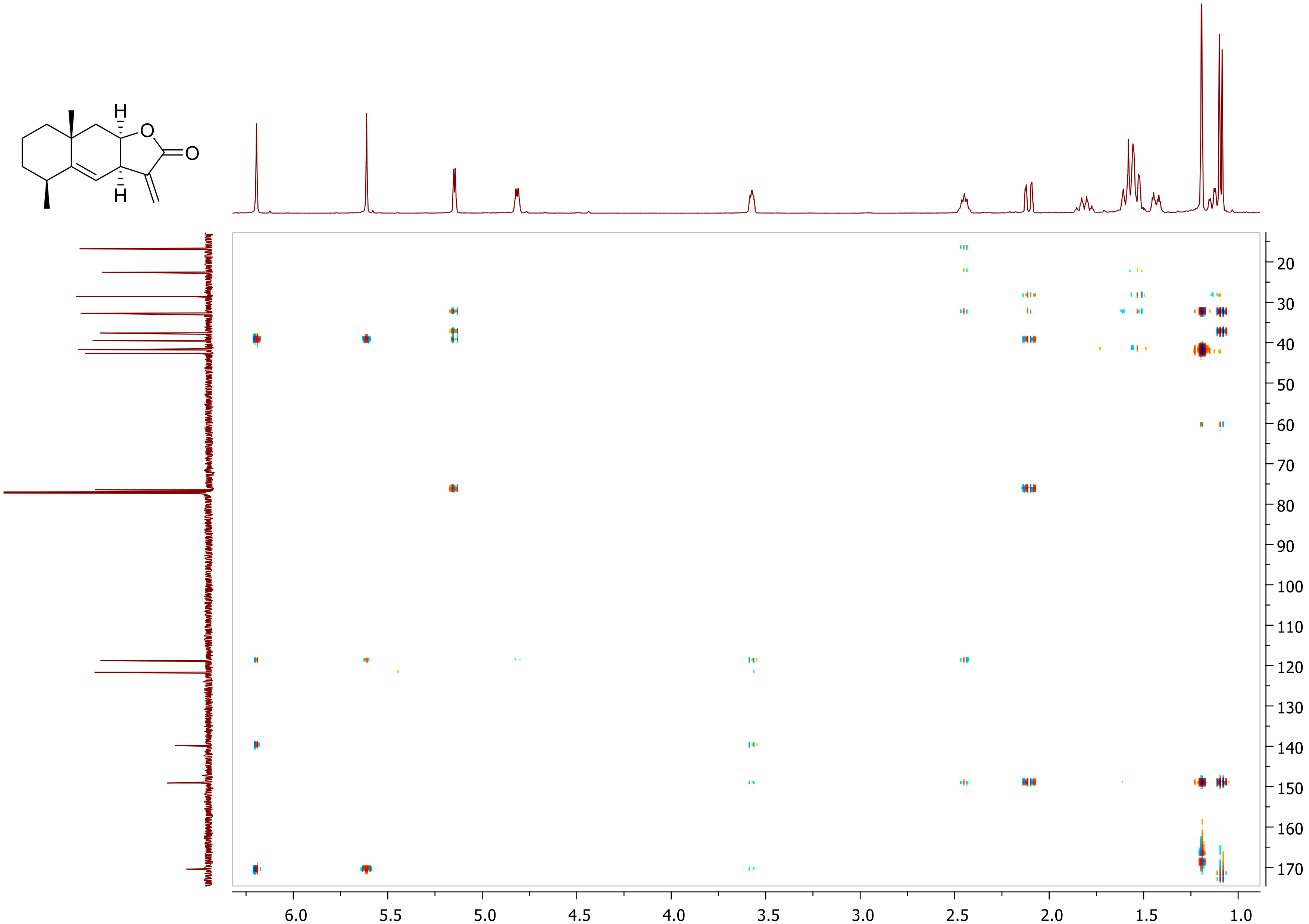

Figure S30. HMBC spectrum of **5** in $CDCl_3$.

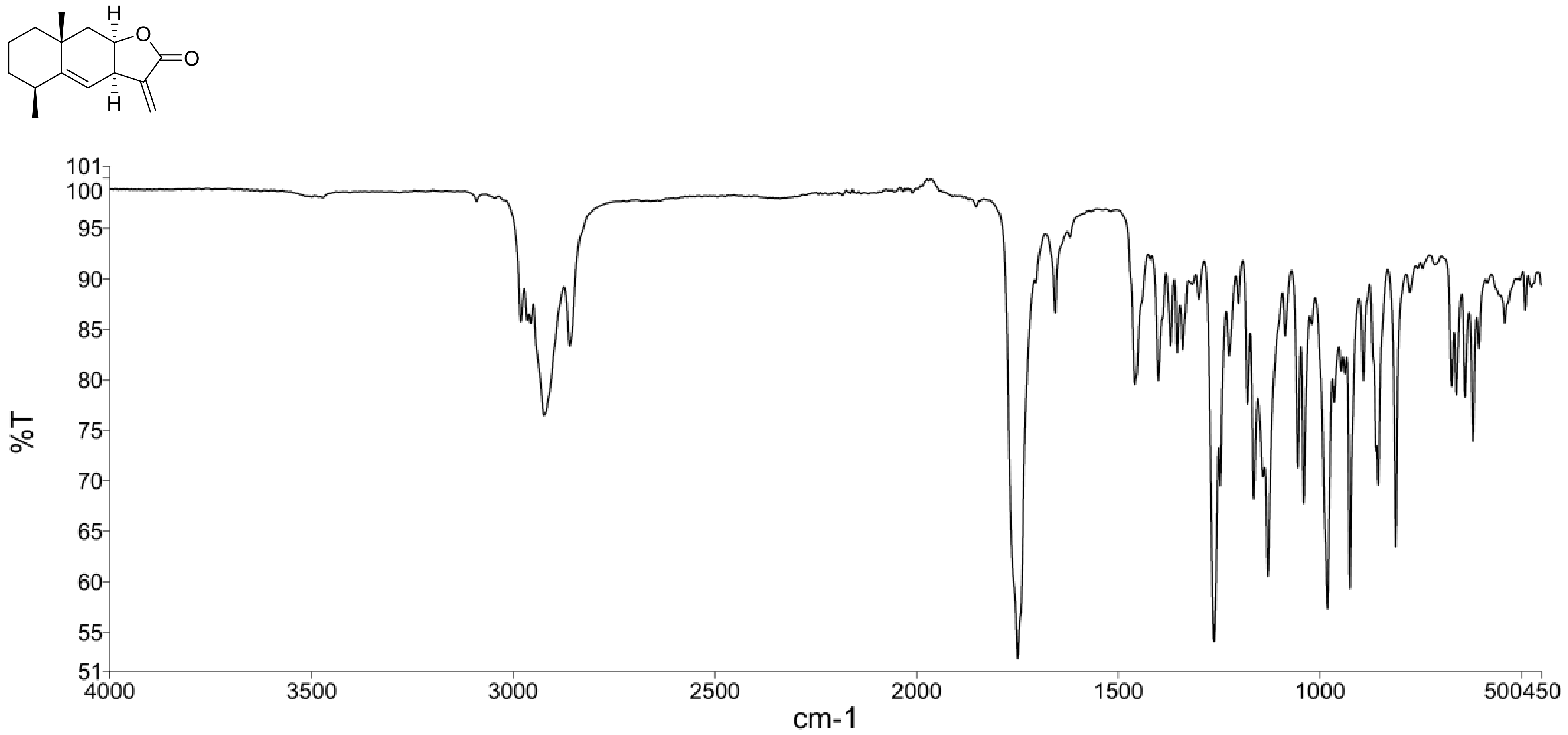

Figure S31. IR spectrum of **5**.

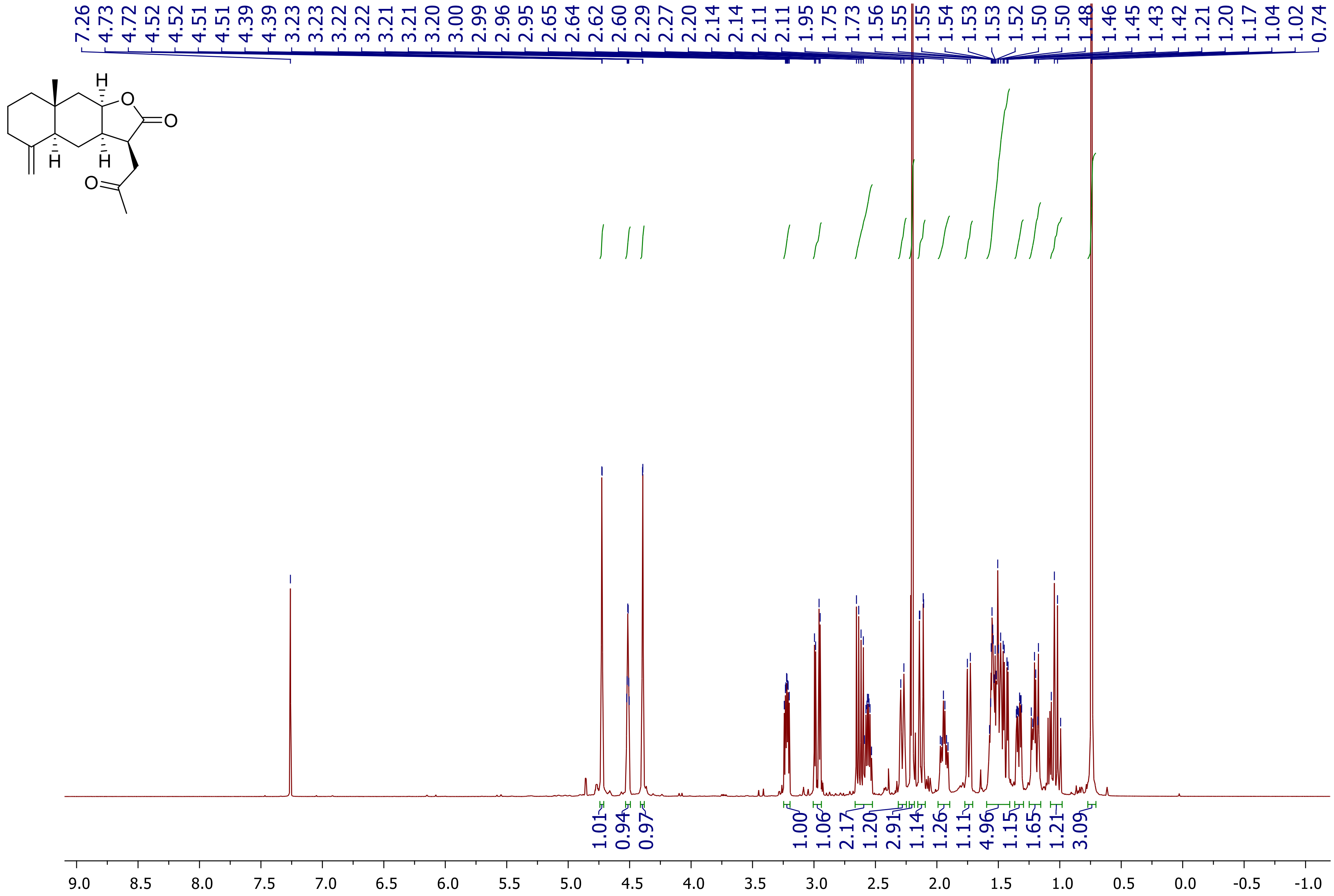

Figure S32. ^{1}H-NMR (400 MHz) spectrum of **7** in $CDCl_3$.

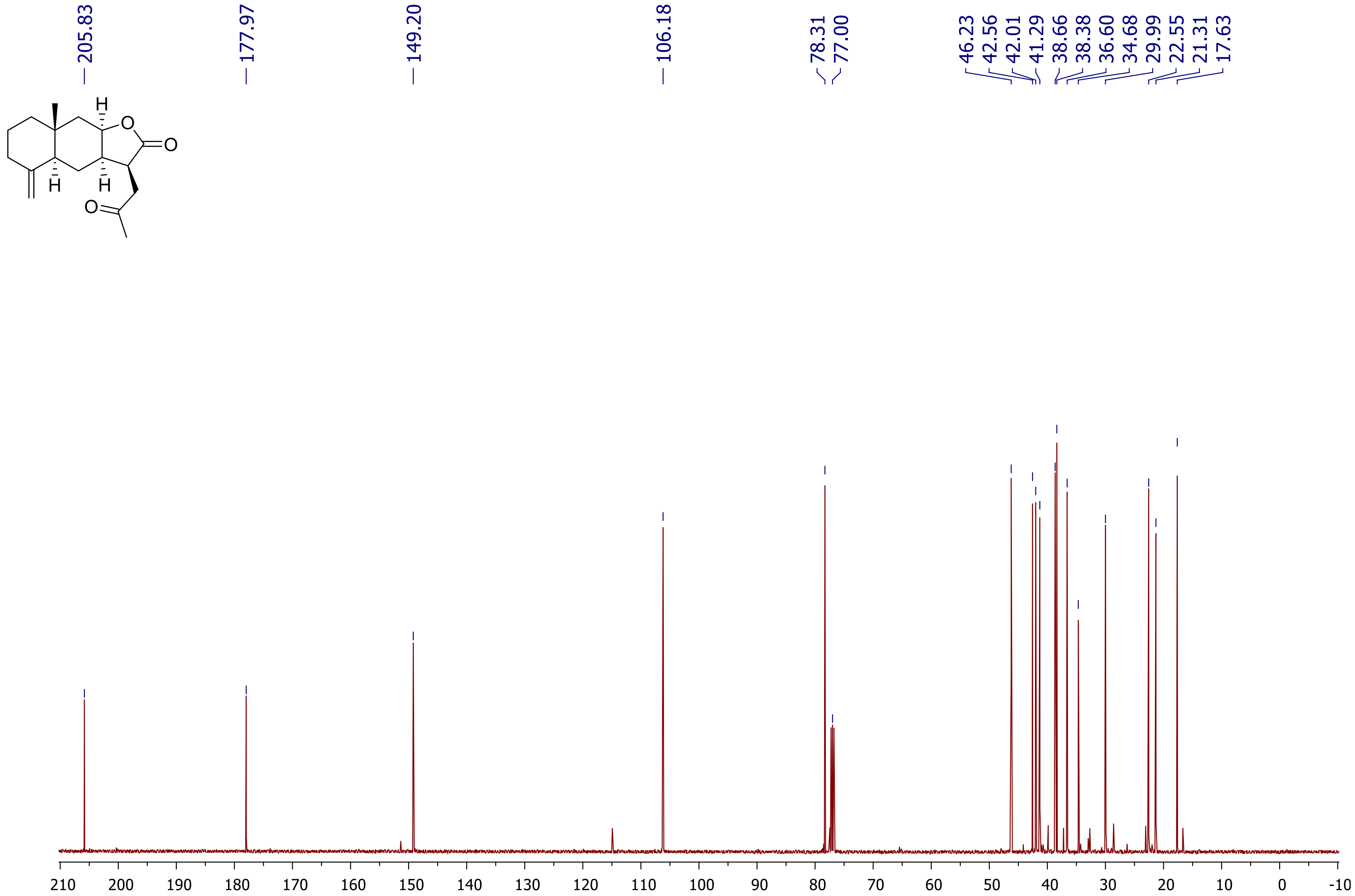

Figure S33. ^{13}C NMR (100 MHz) spectrum of **7** in $CDCl_3$.

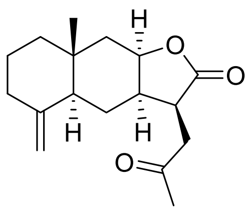

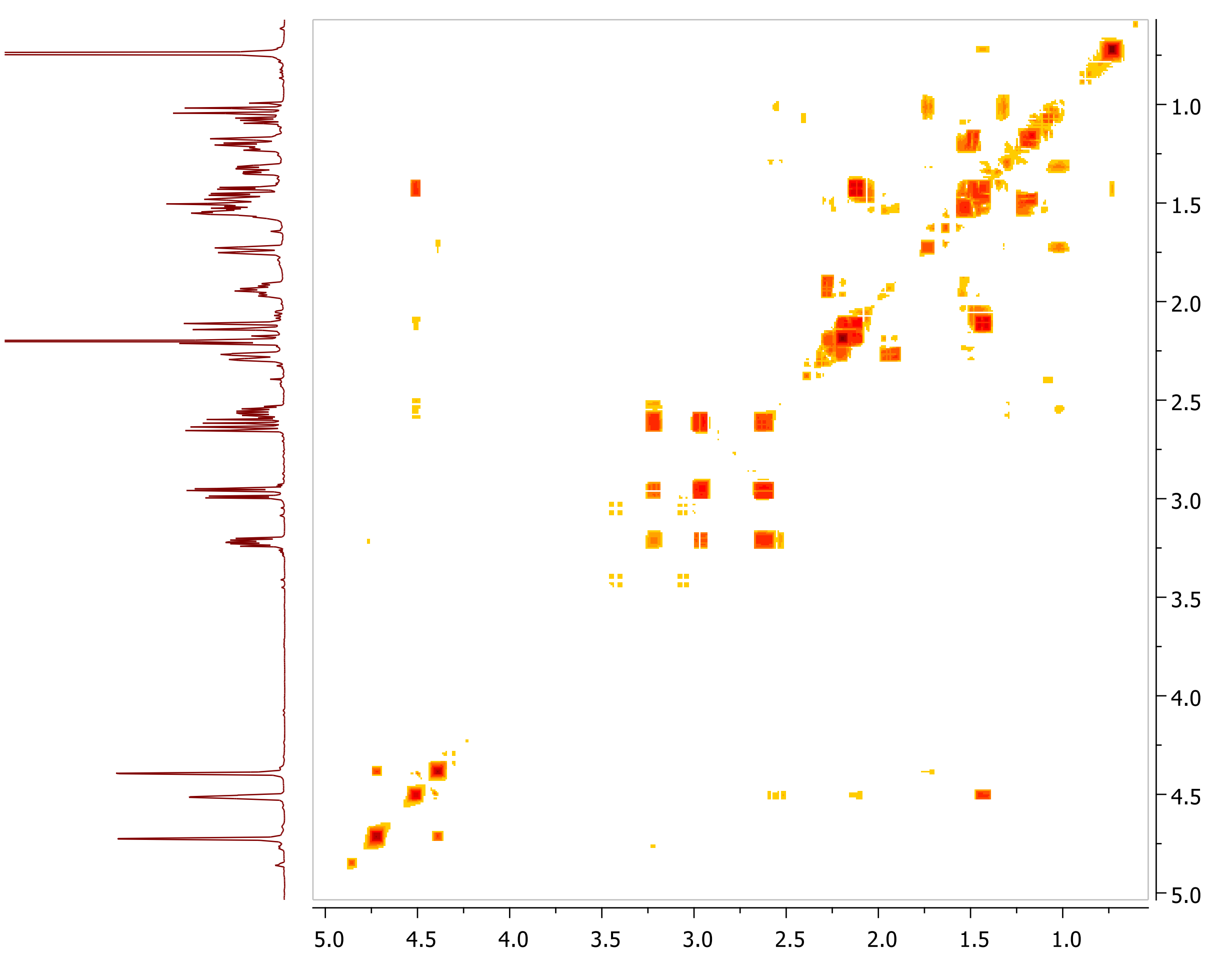

Figure S34. ^{1}H-^{1}H-COSY spectrum of **7** in $CDCl_3$.

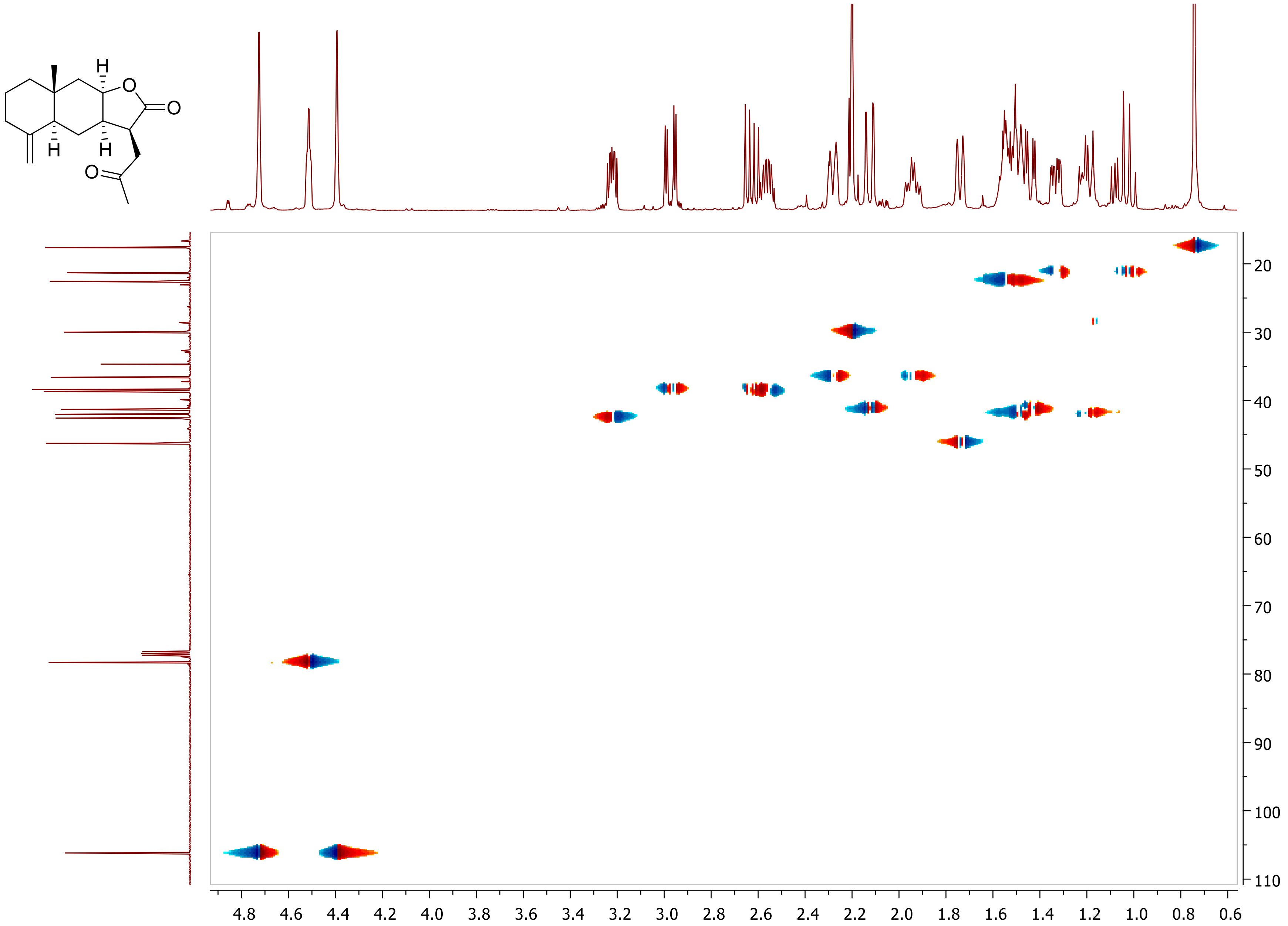

Figure S35. HSQC spectrum of **7** in $CDCl_3$.

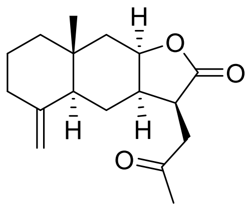

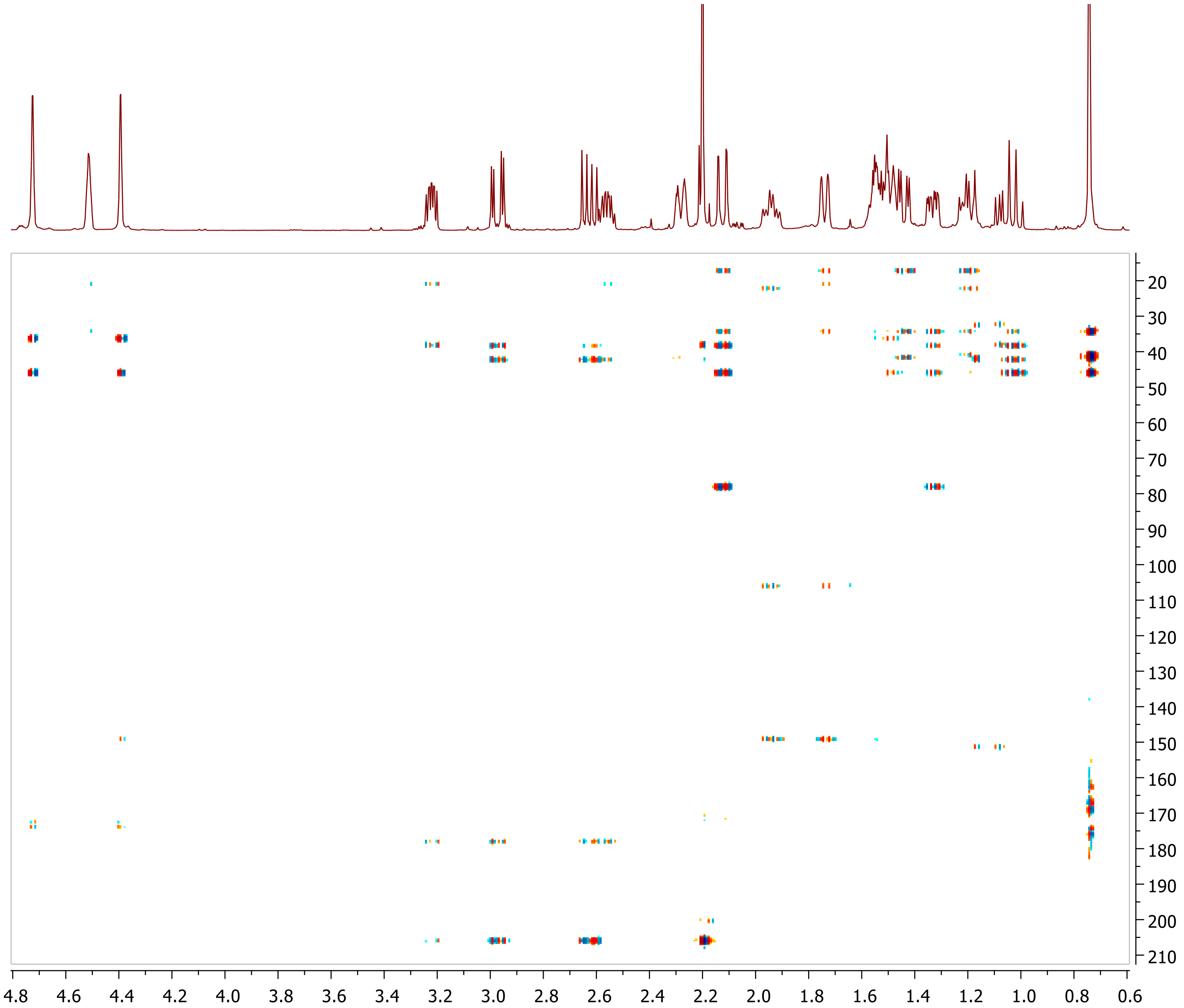

Figure S36. HMBC spectrum of **7** in $CDCl_3$.

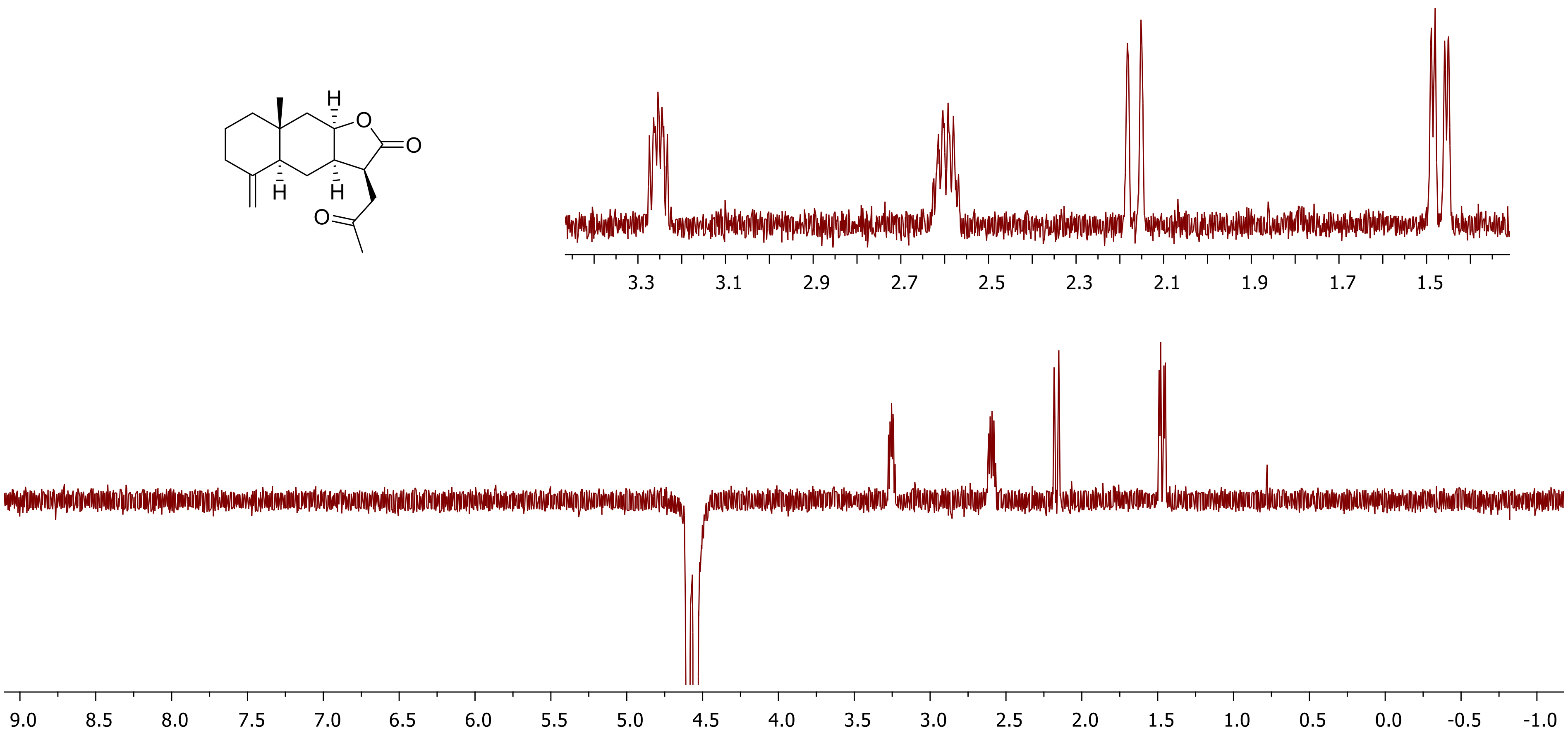

Figure S37. 1D-NOESY spectrum of **7** in $CDCl_3$.

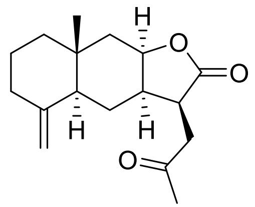

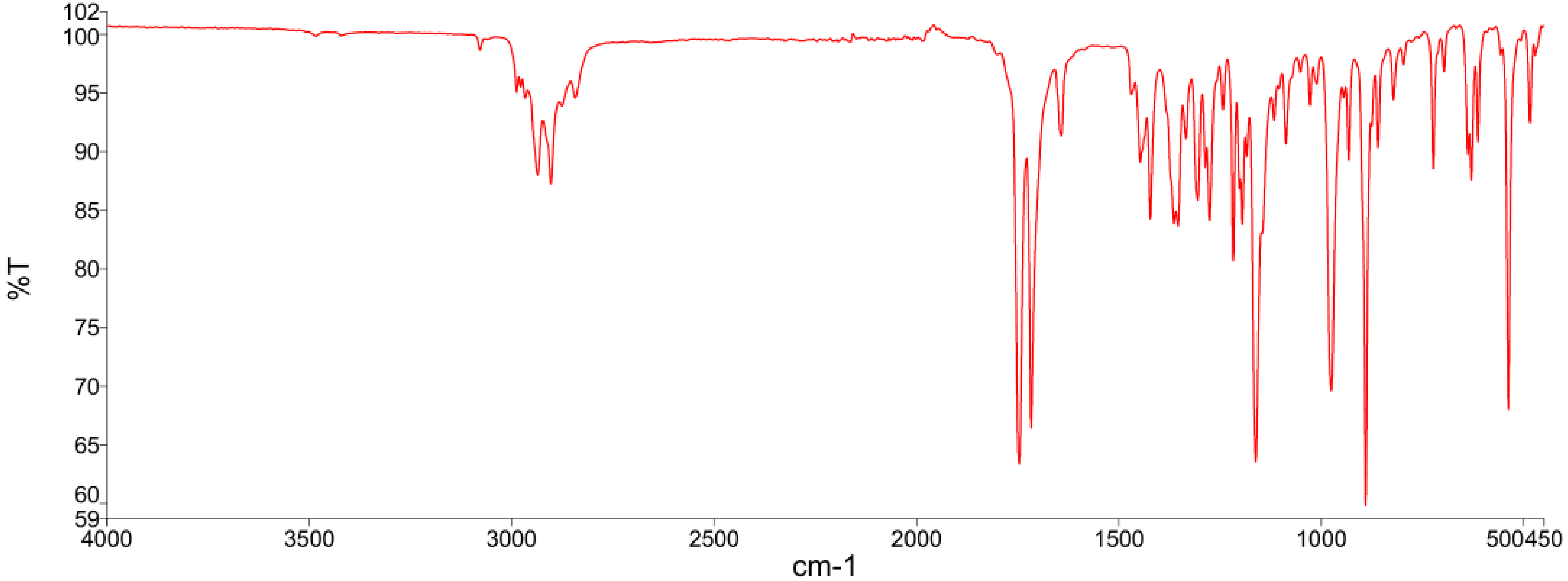

Figure S38. IR spectrum of **7**.

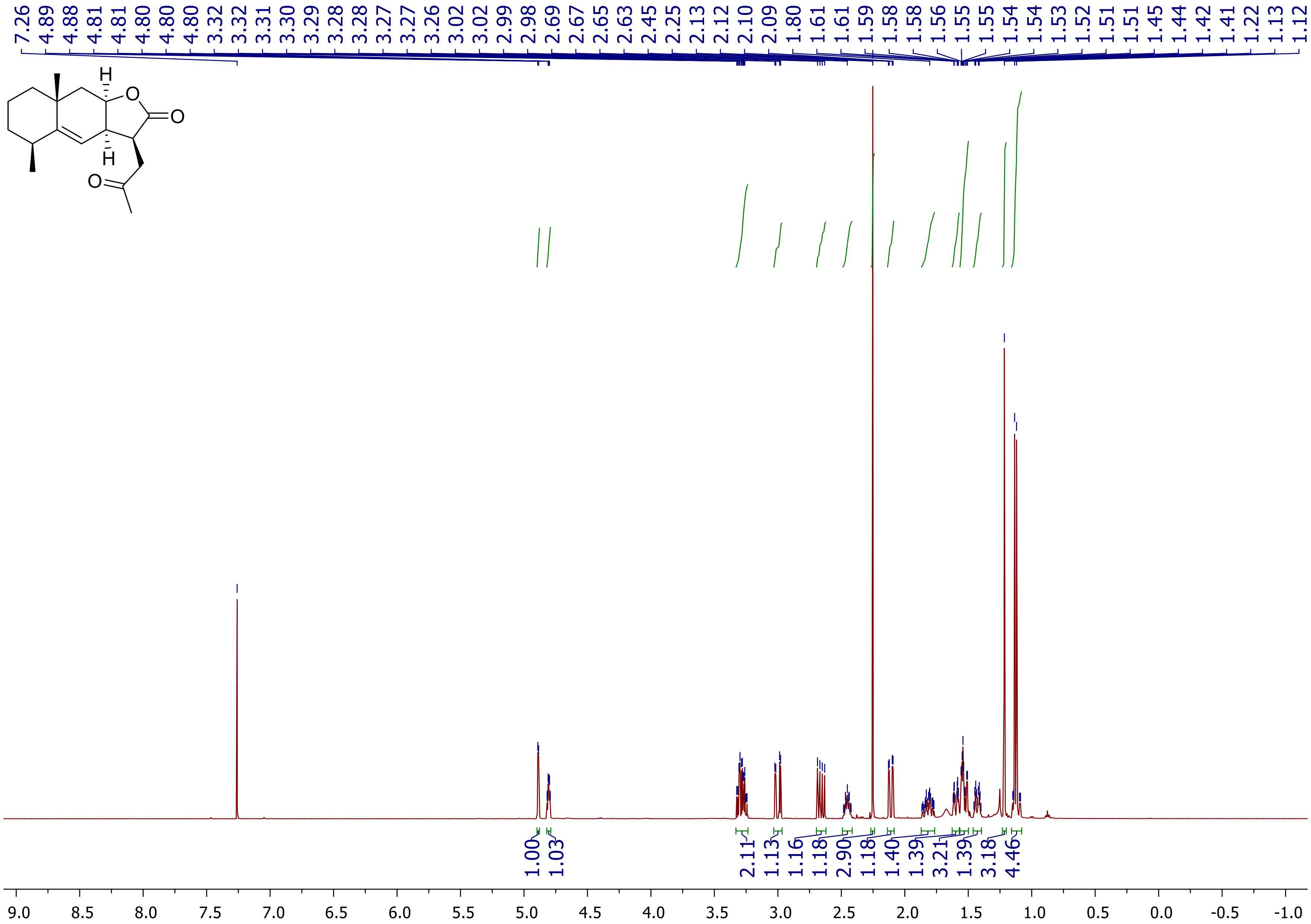

Figure S39. ^{1}H-NMR (500 MHz) spectrum of **8** in $CDCl_3$.

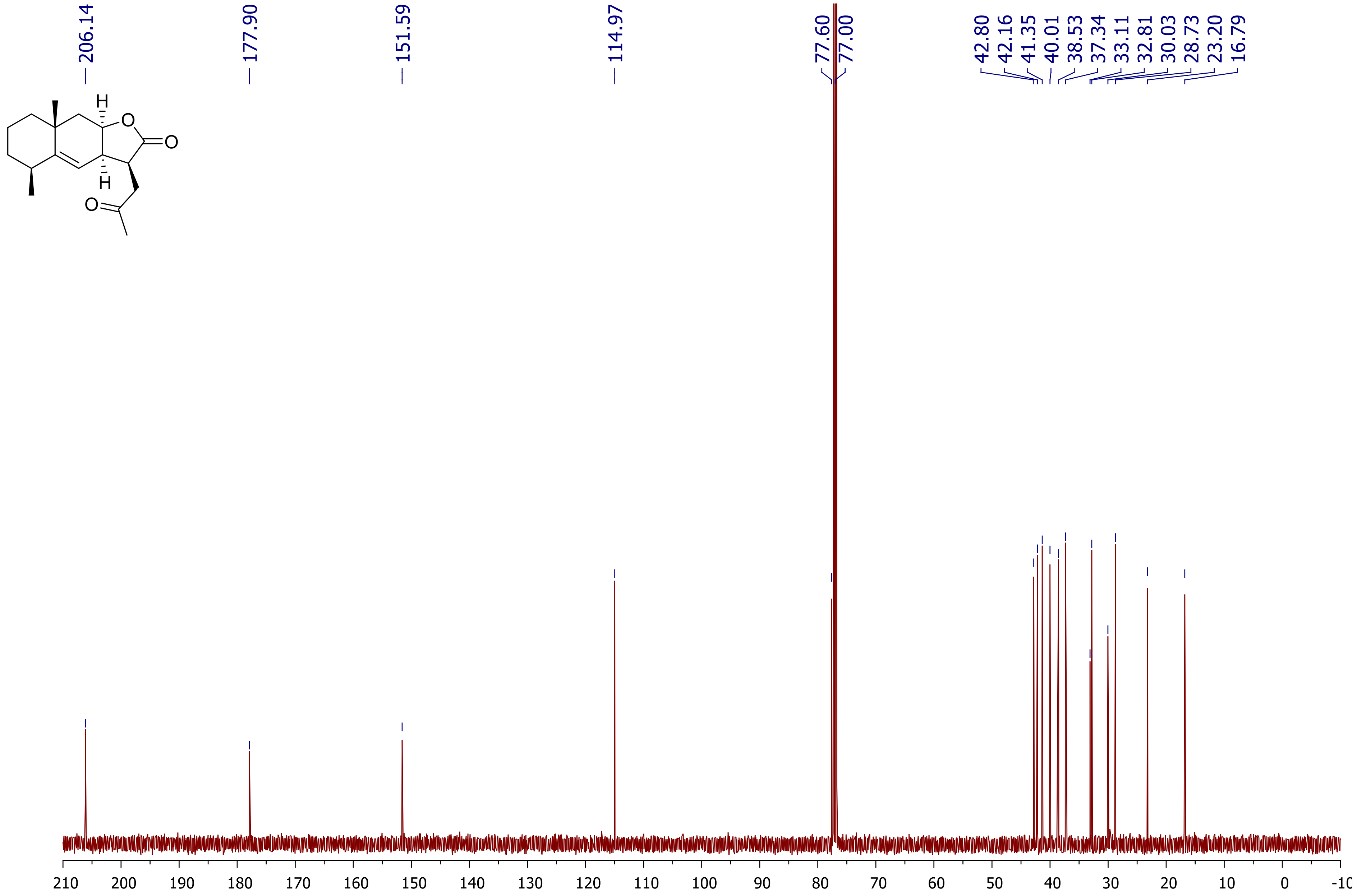

Figure S40. ^{13}C NMR (125 MHz) spectrum of **8** in $CDCl_3$.

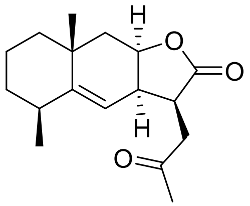

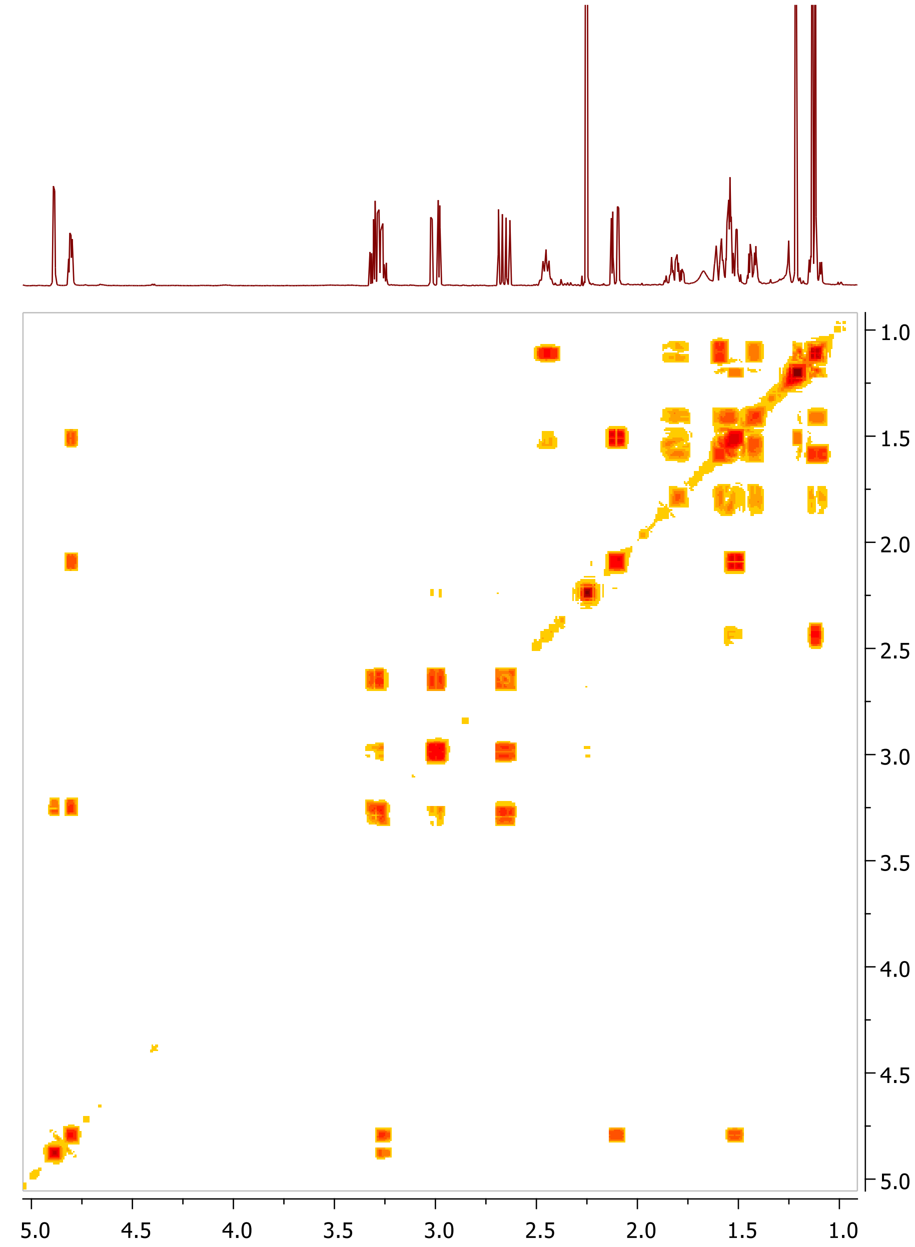

Figure S41. ^{1}H-^{1}H-COSY spectrum of **8** in $CDCl_3$.

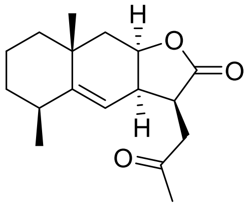

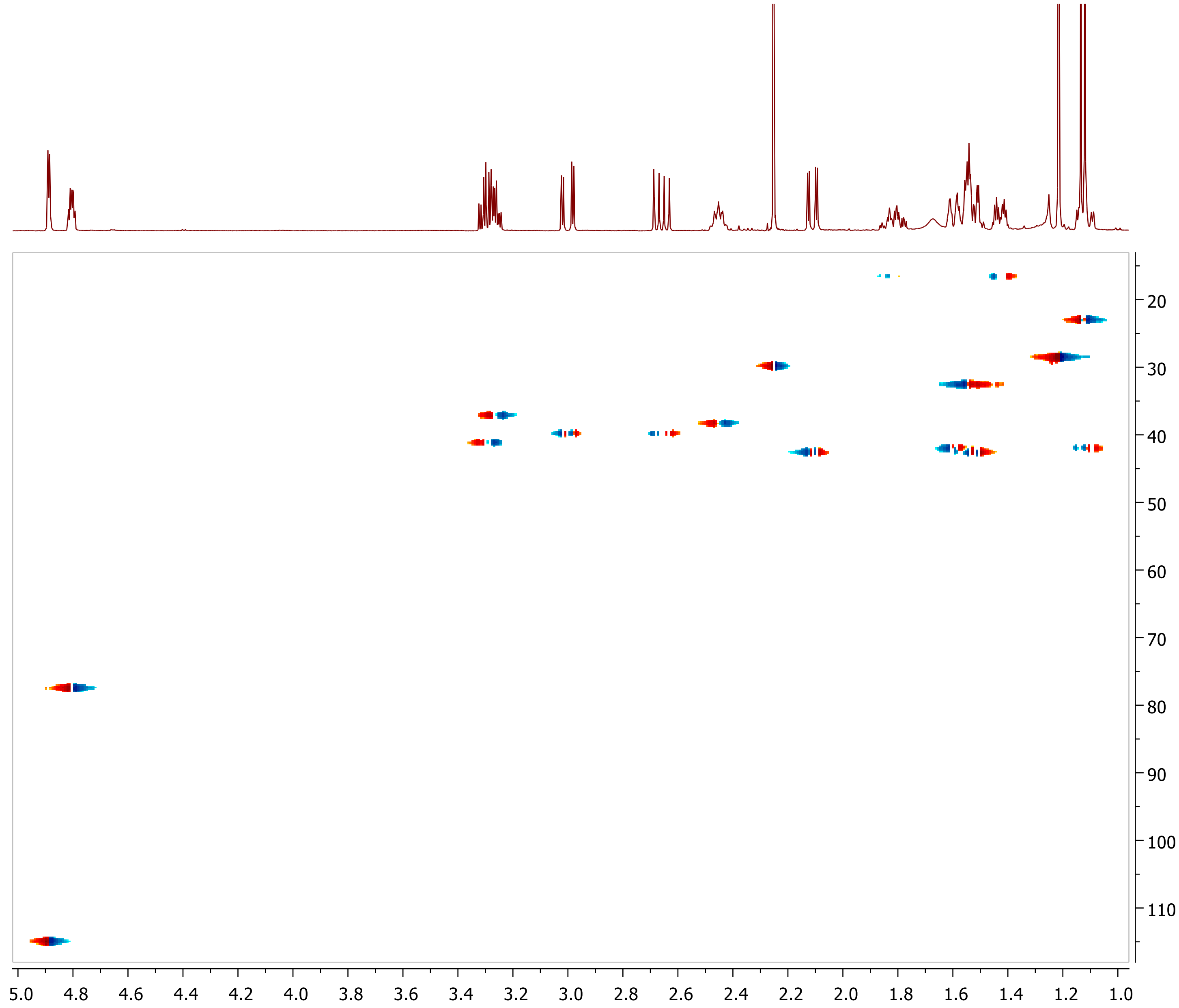

Figure S42. HSQC spectrum of **8** in $CDCl_3$.

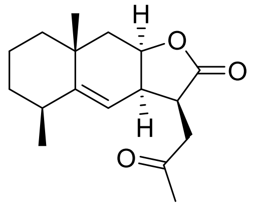

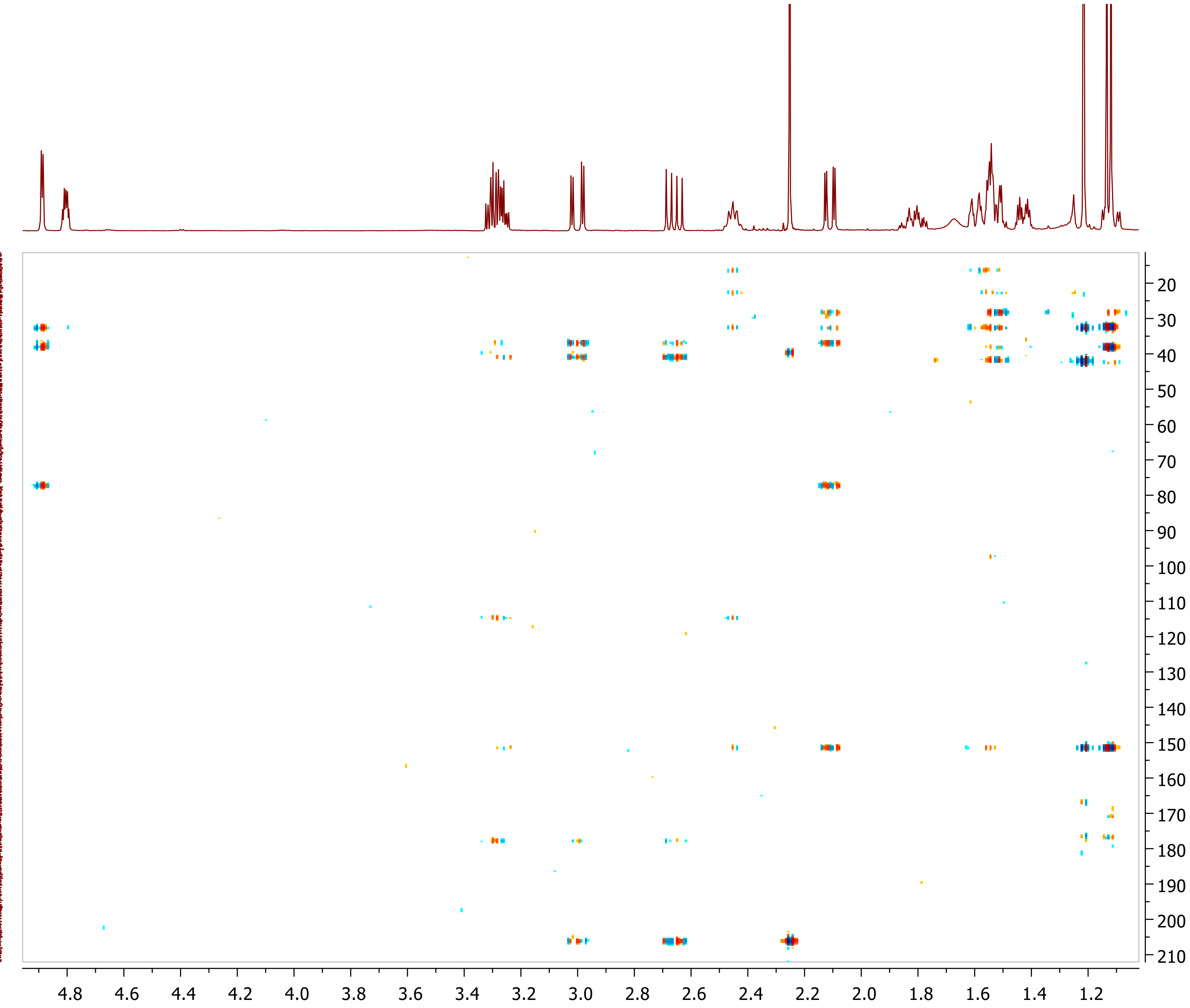

Figure S43. HMBC spectrum of **8** in $CDCl_3$.

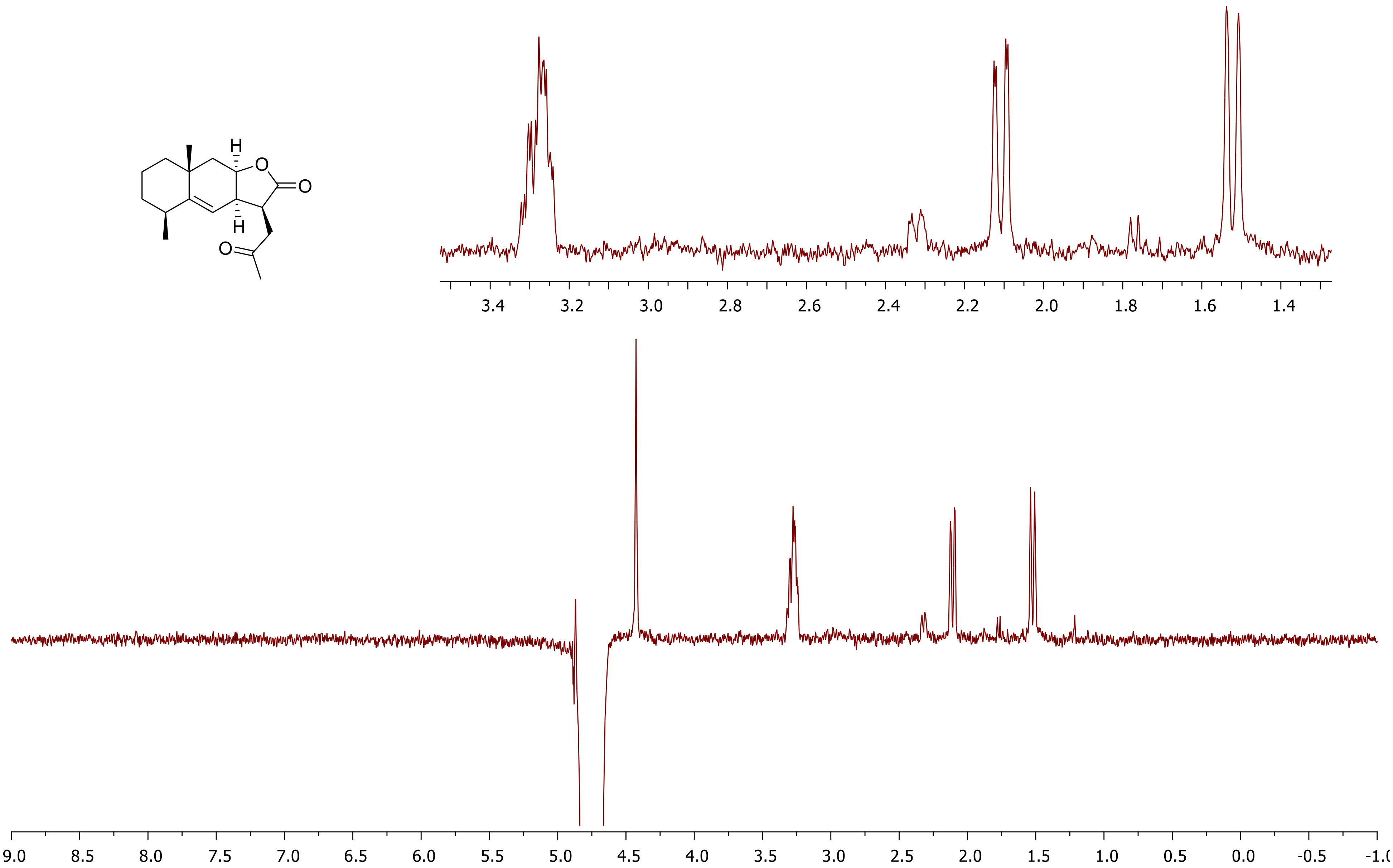

Figure S44. 1D-NOESY spectrum of **8** in $CDCl_3$.

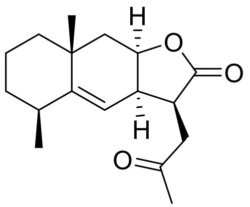

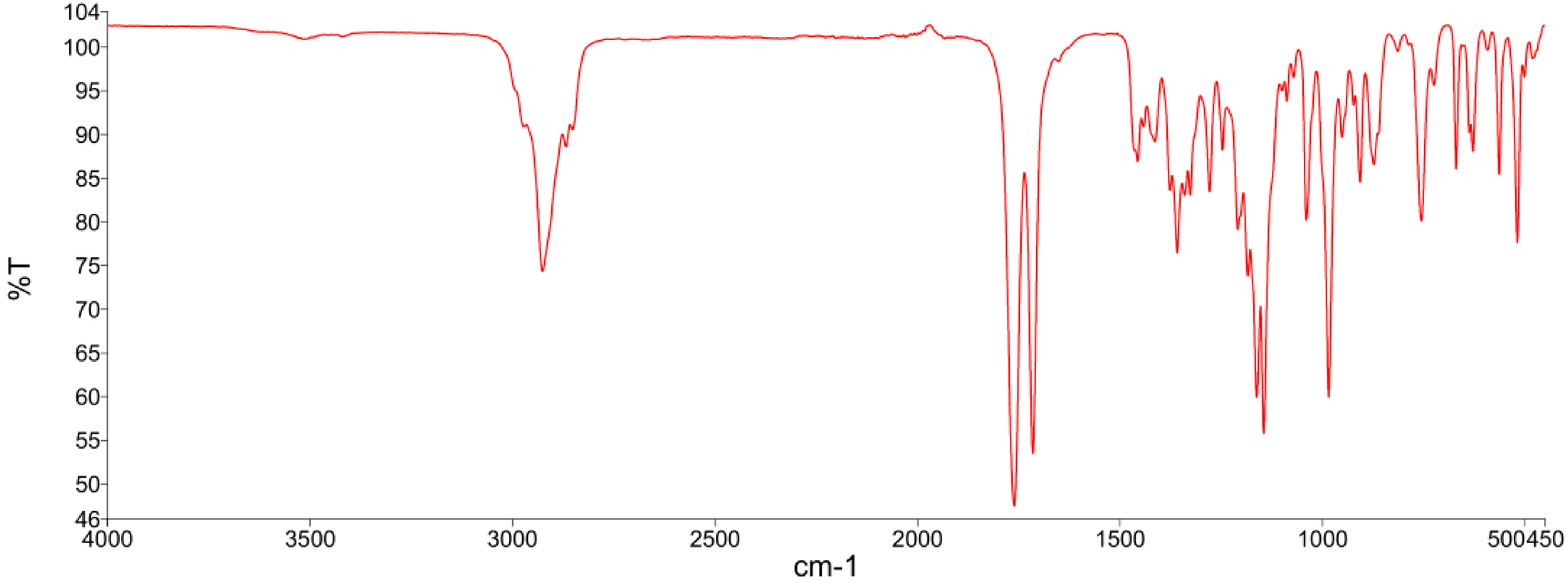

Figure S45. IR spectrum of **8**.

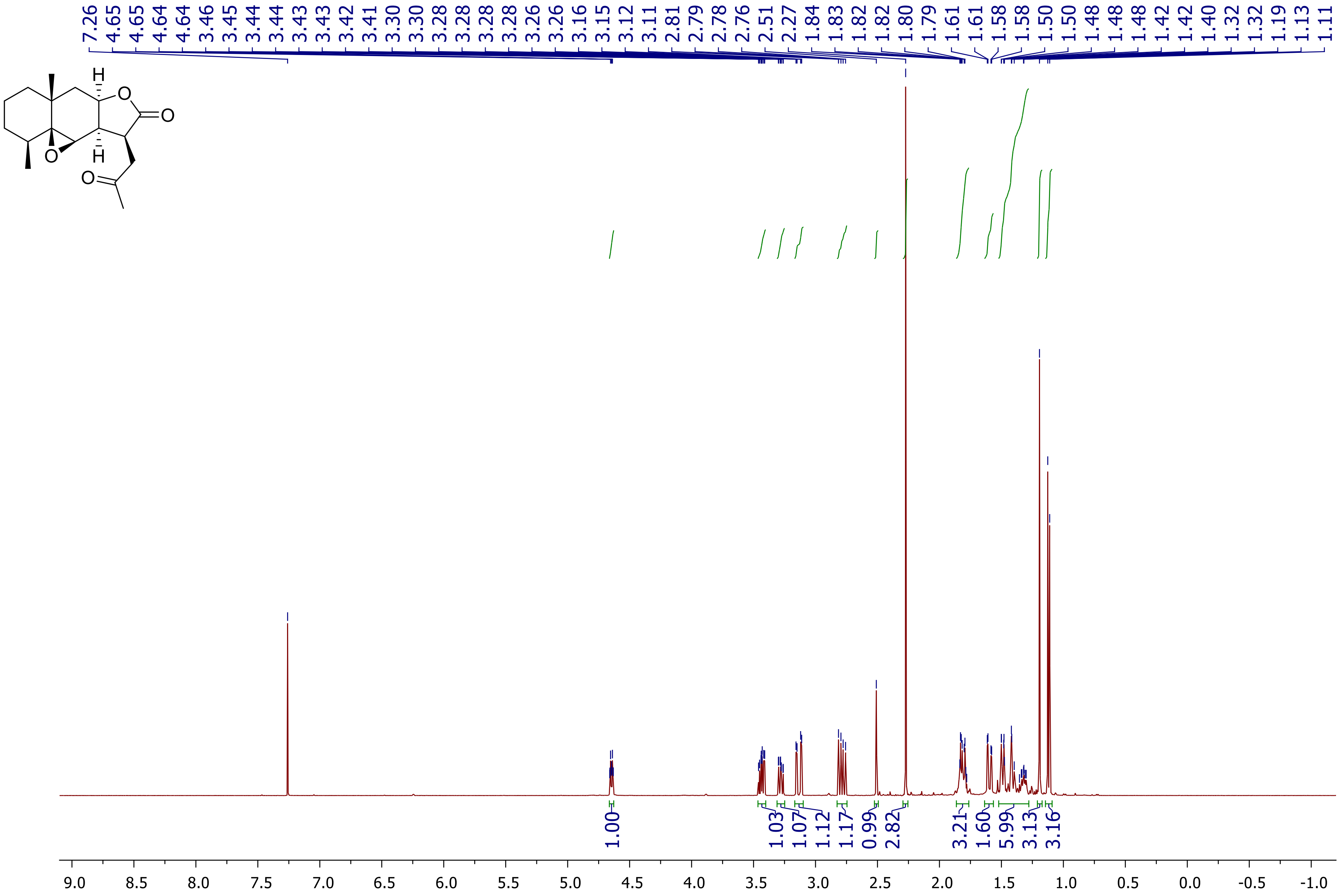

Figure S46. ^{1}H-NMR (500 MHz) spectrum of **9** in $CDCl_3$.

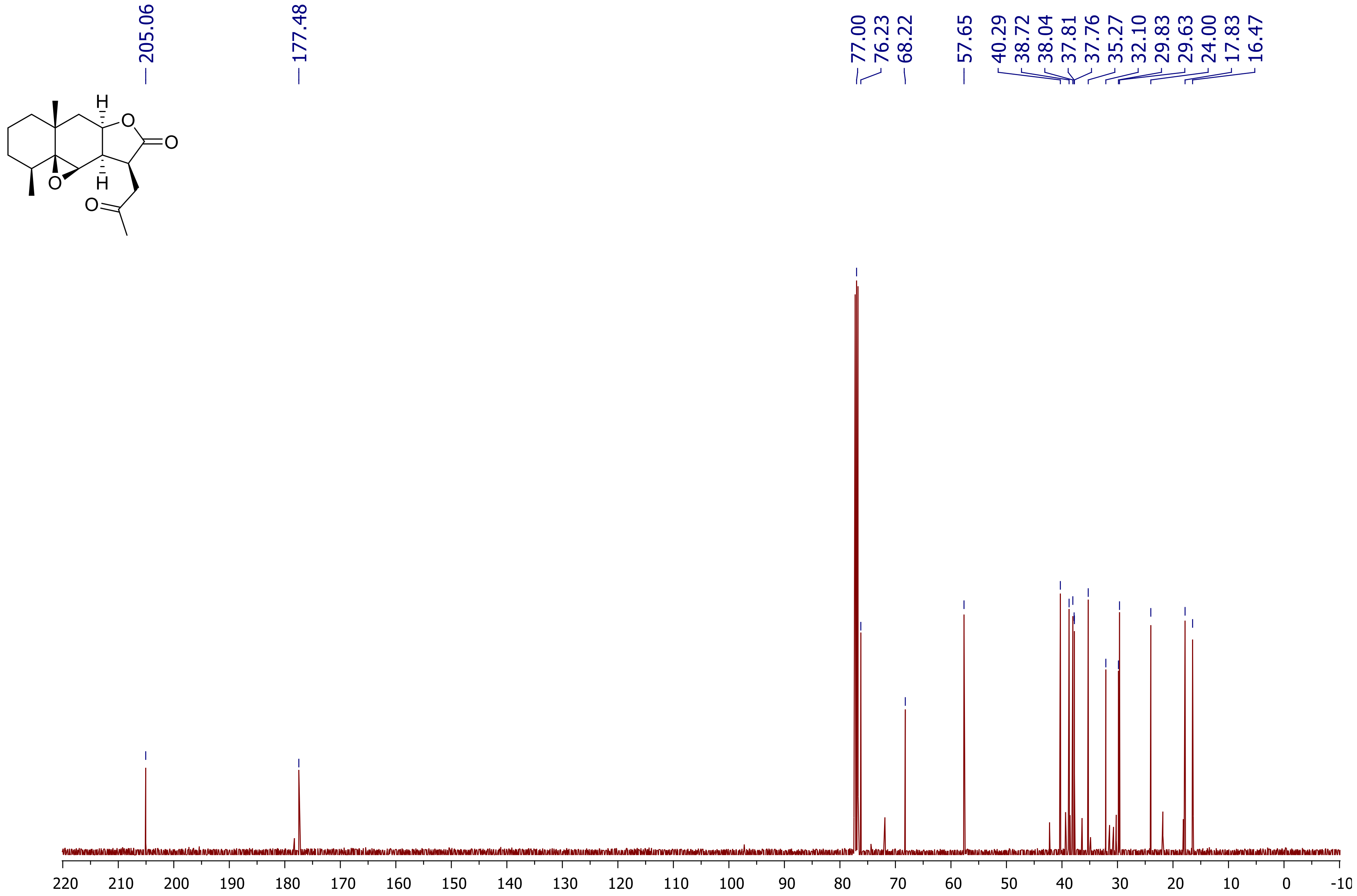

Figure S47. ^{13}C NMR (125 MHz) spectrum of **9** in $CDCl_3$.

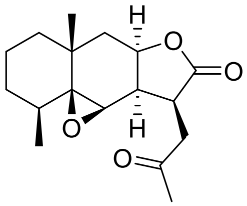

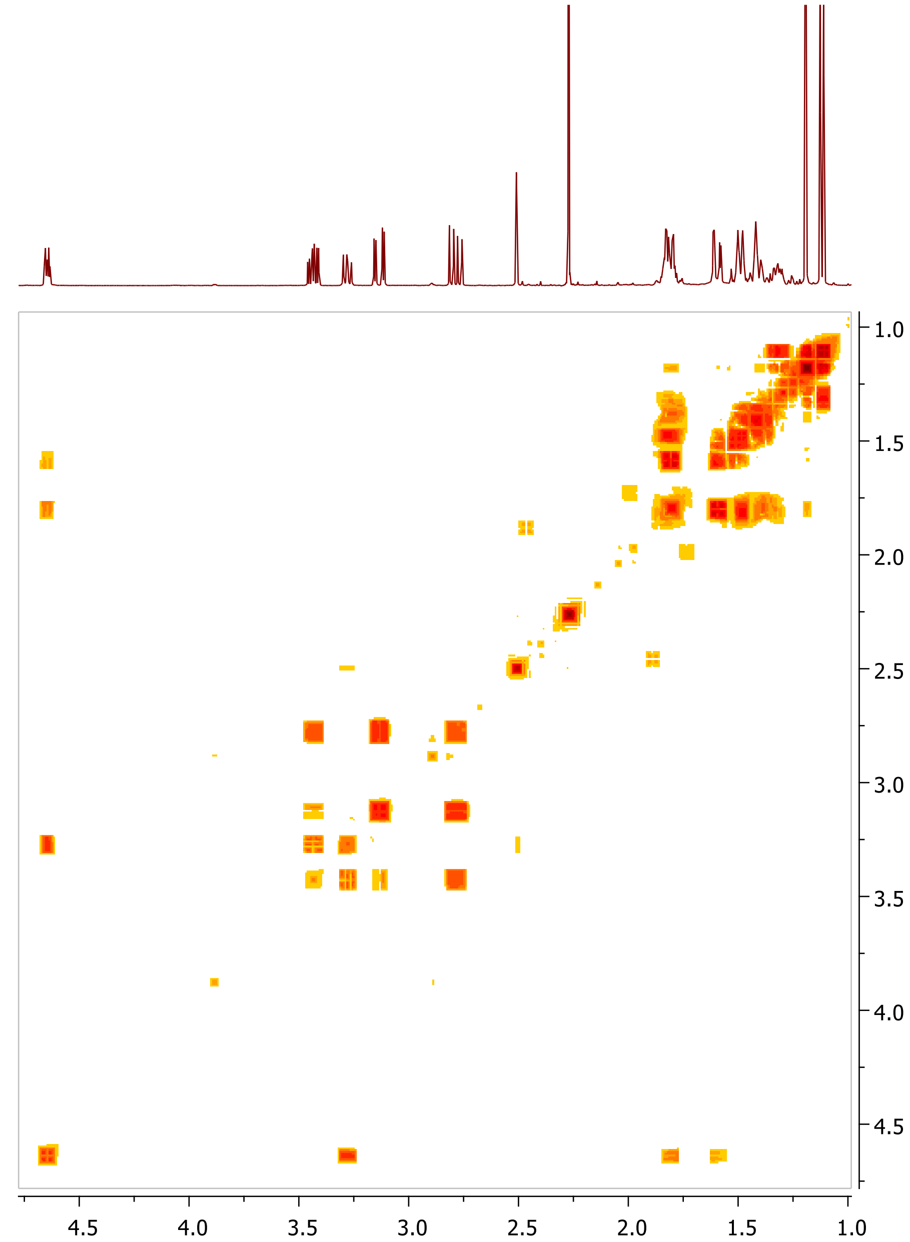

Figure S48. ^{1}H-^{1}H-COSY spectrum of **9** in $CDCl_3$.

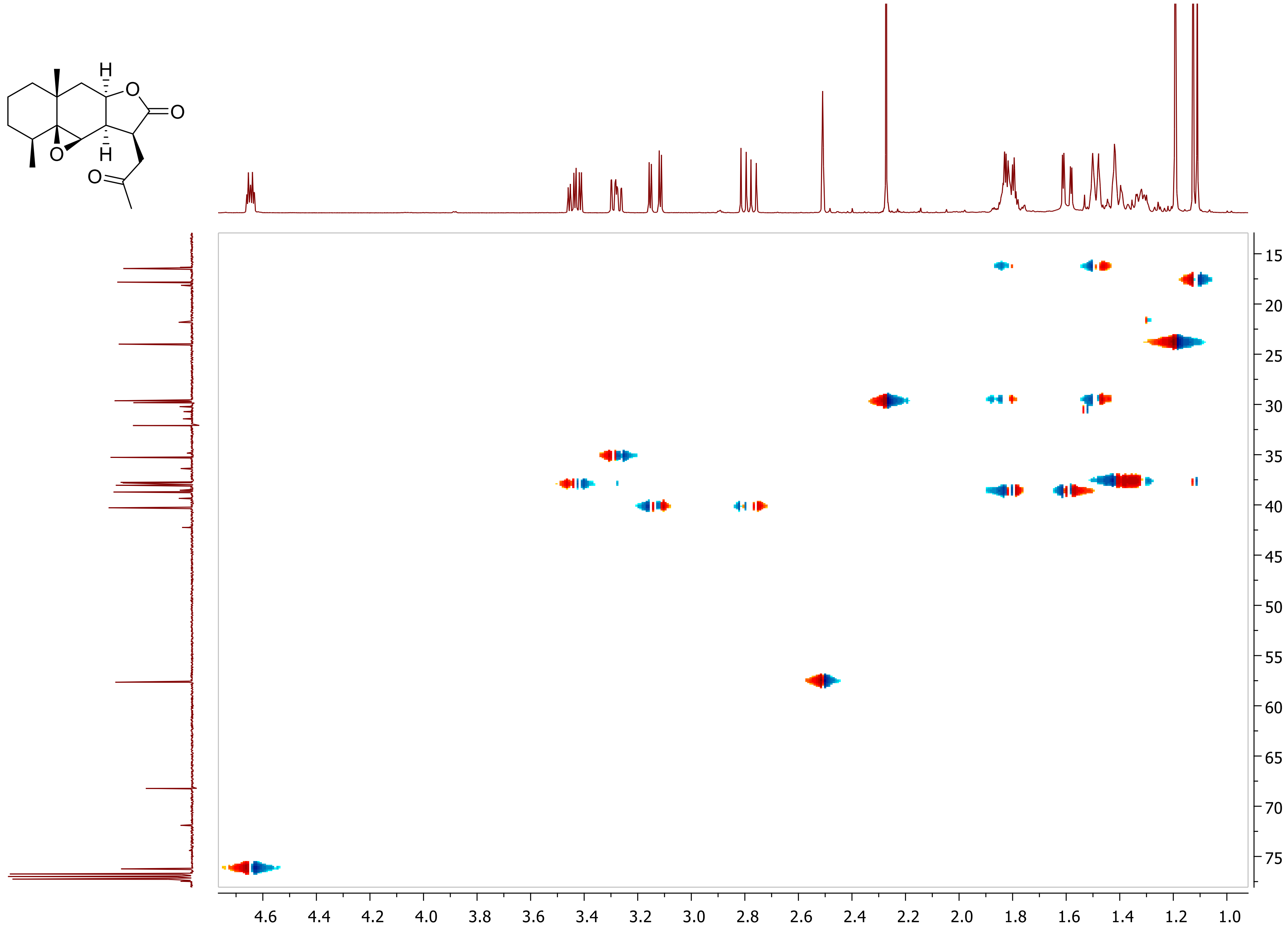

Figure S49. HSQC spectrum of **9** in $CDCl_3$.

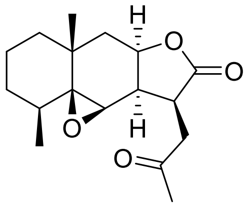

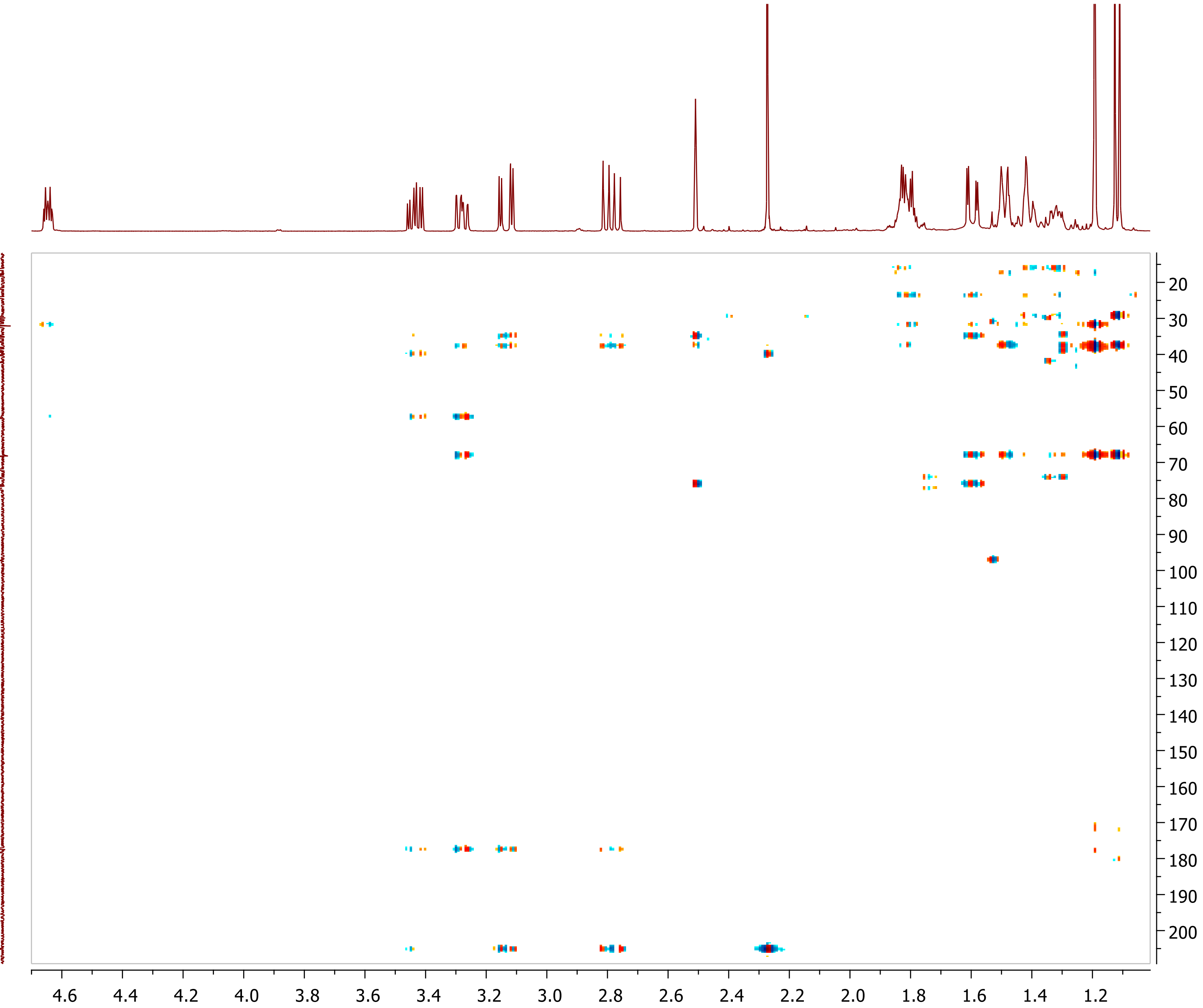

Figure S50. HMBC spectrum of **9** in $CDCl_3$.

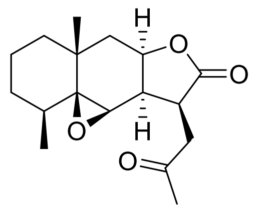

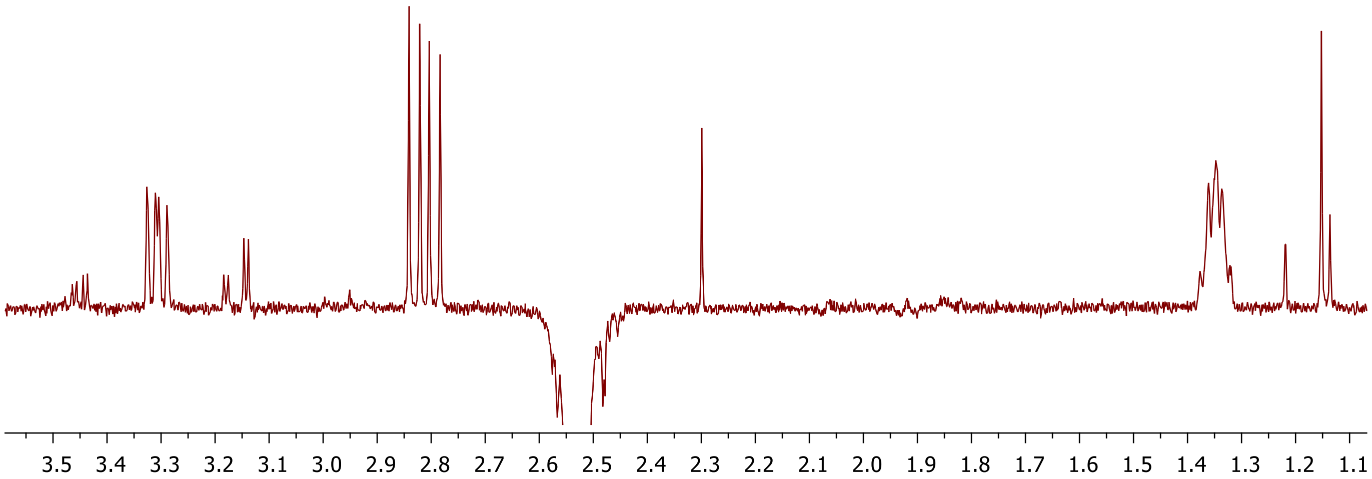

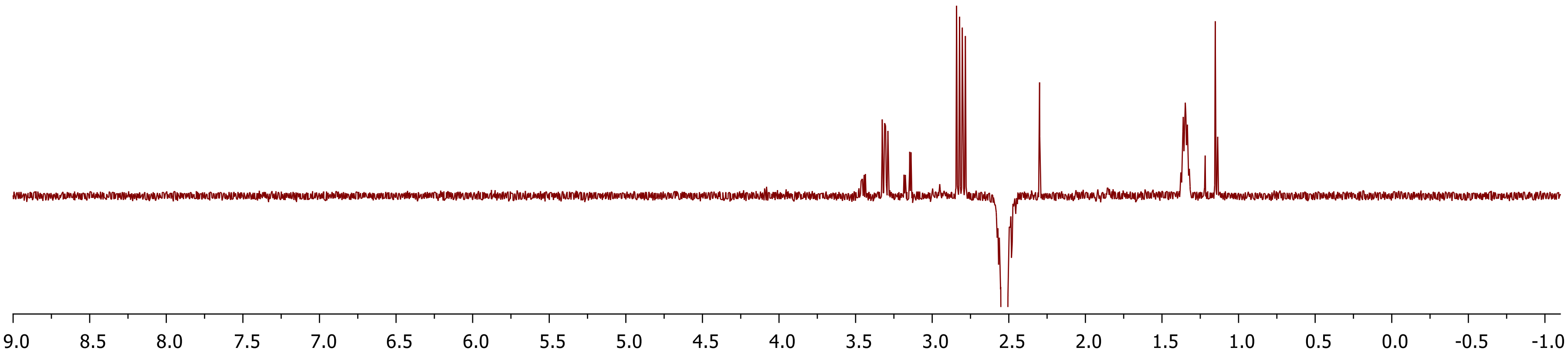

Figure S51. 1D-NOESY spectrum of **9** in $CDCl_3$.

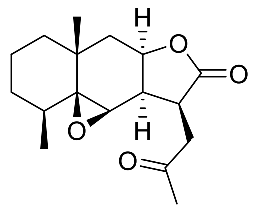

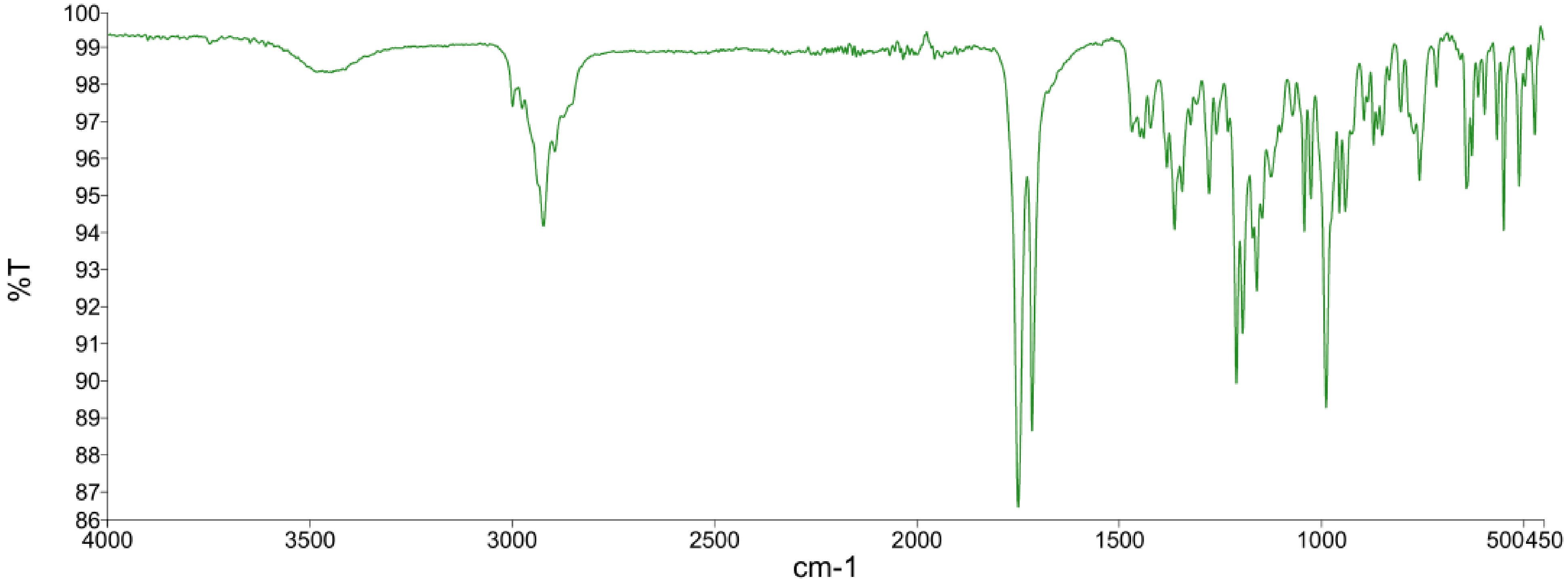

Figure S52. IR spectrum of **9**.

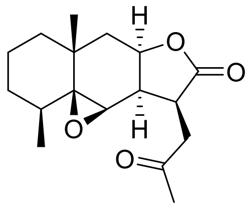

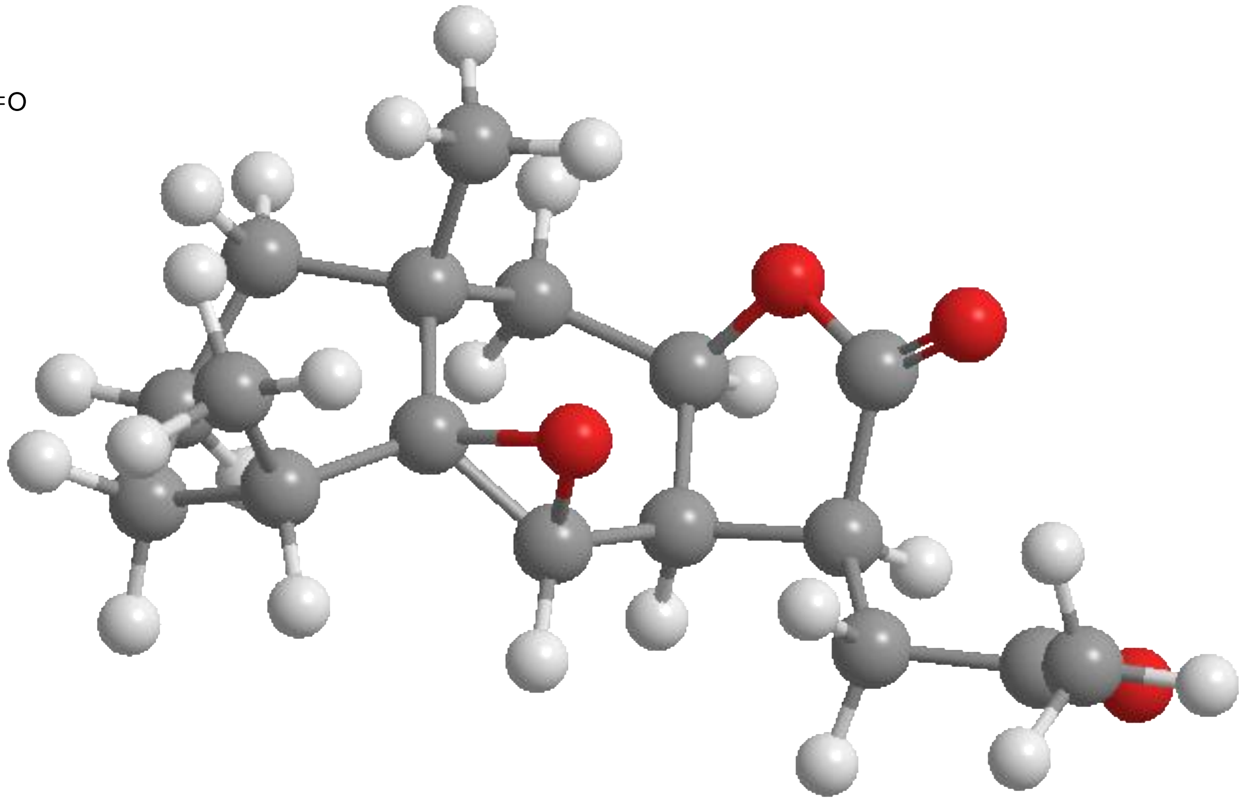

Figure S53. 3D model of **9**.

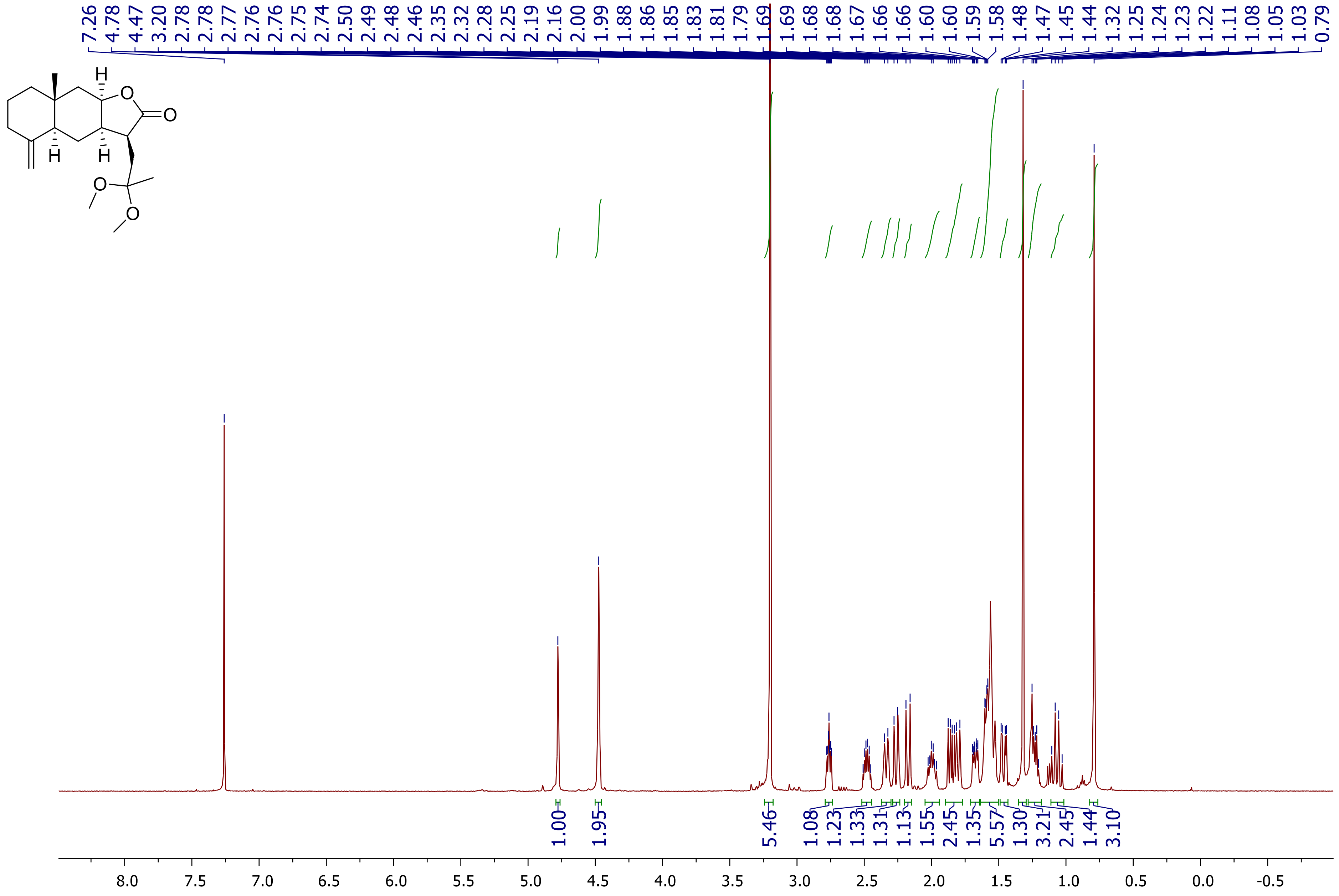

Figure S54. ^{1}H-NMR (500 MHz) spectrum of **10** in $CDCl_3$.

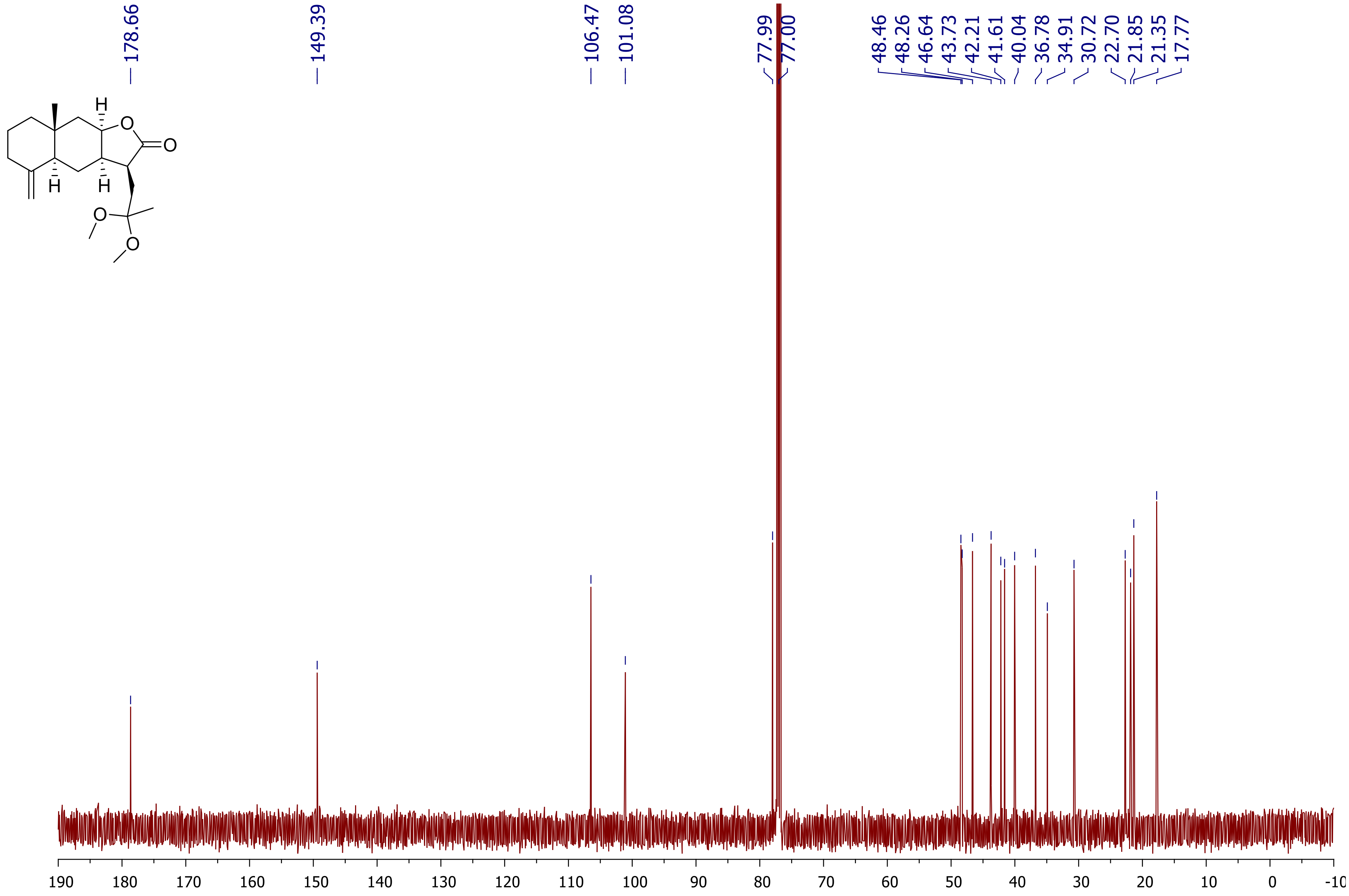

Figure S55. ^{13}C NMR (125 MHz) spectrum of **10** in $CDCl_3$.

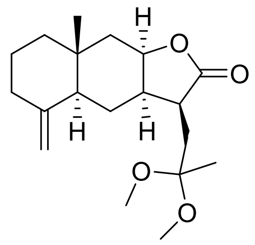

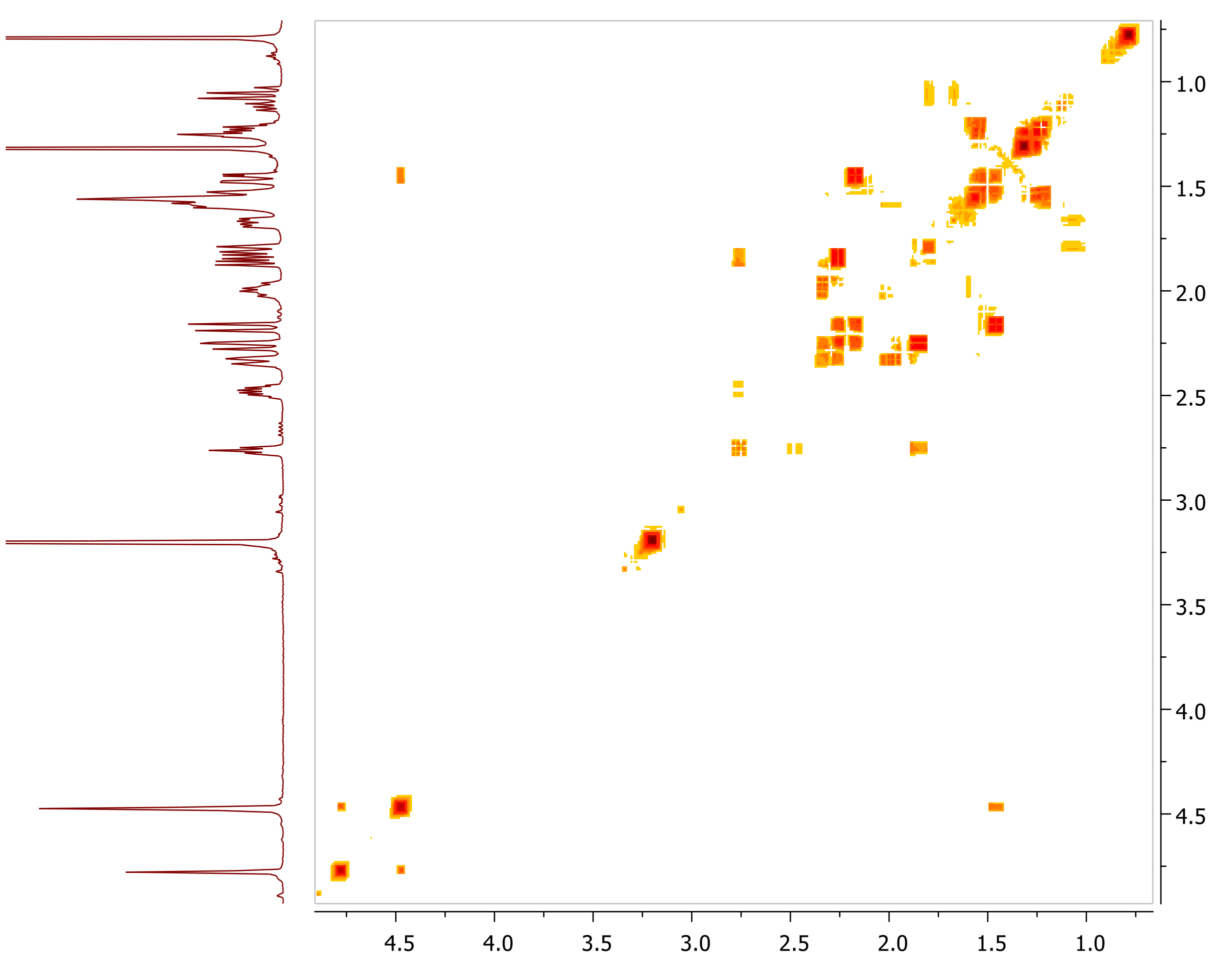

Figure S56. ^{1}H-^{1}H-COSY spectrum of **10** in $CDCl_3$.

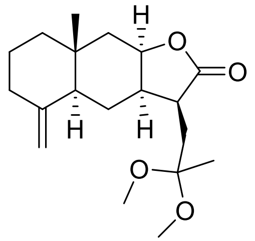

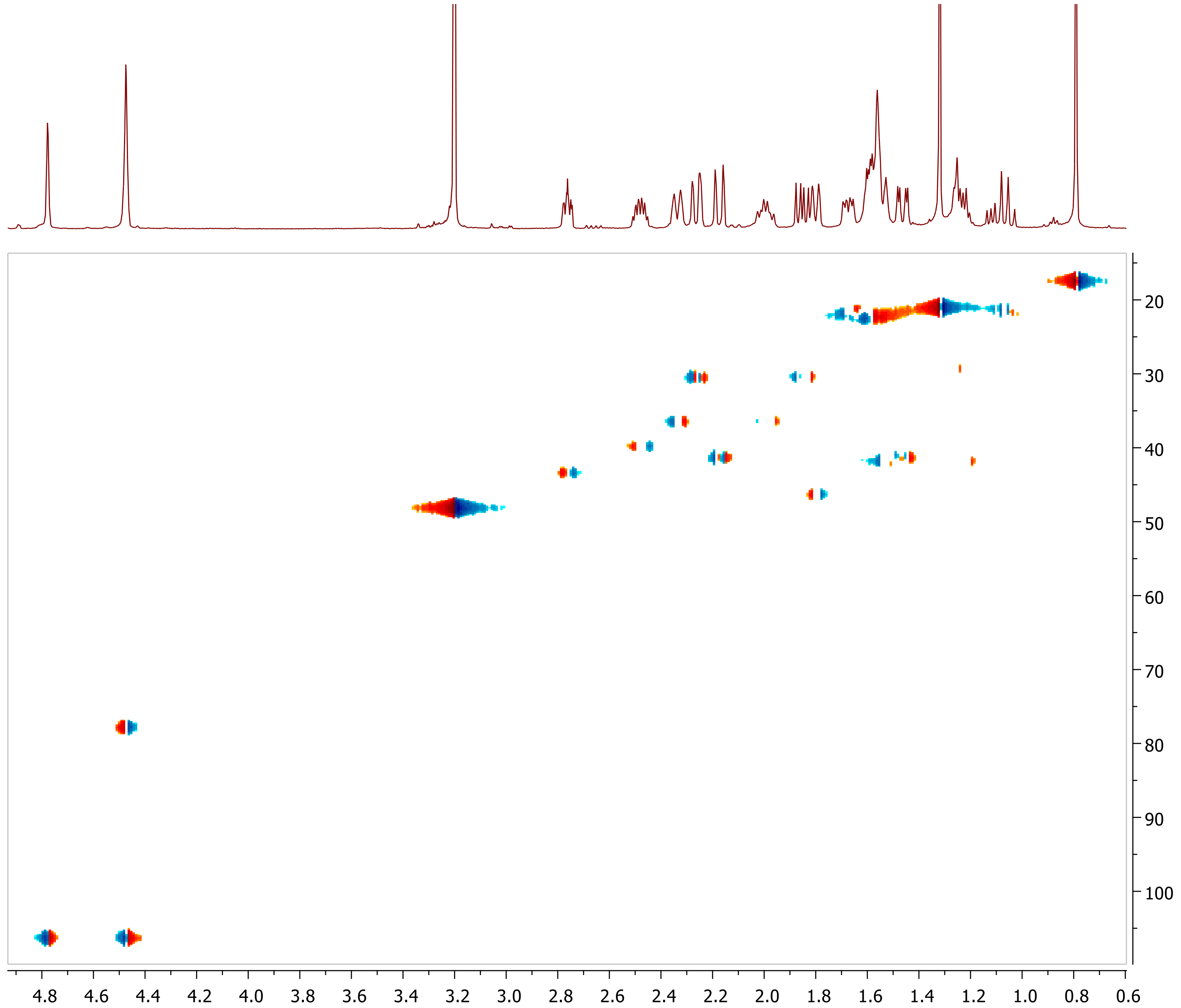

Figure S57. HSQC spectrum of **10** in $CDCl_3$.

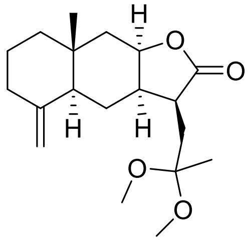

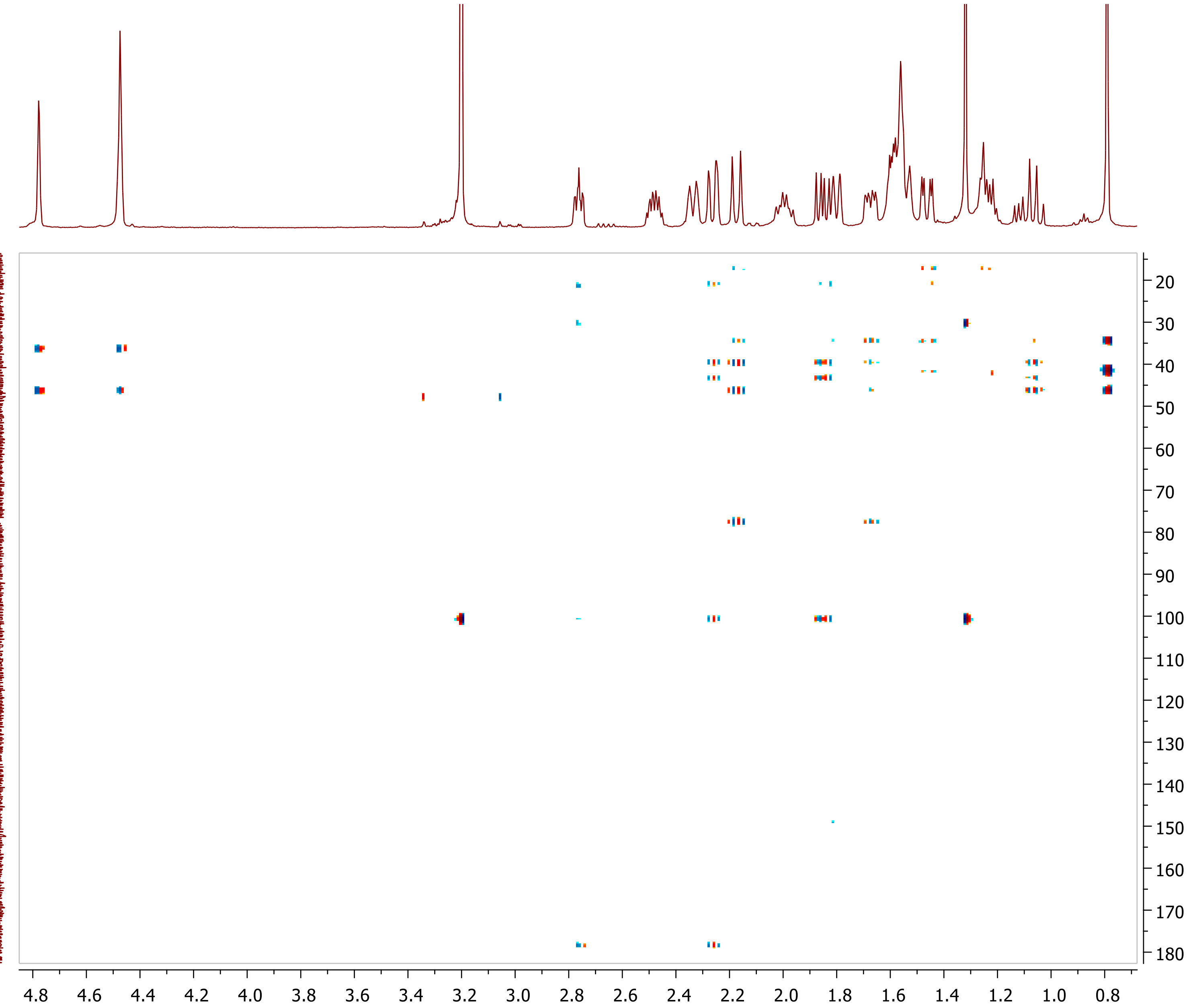

Figure S58. HMBC spectrum of **10** in $CDCl_3$.

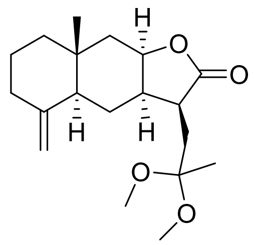

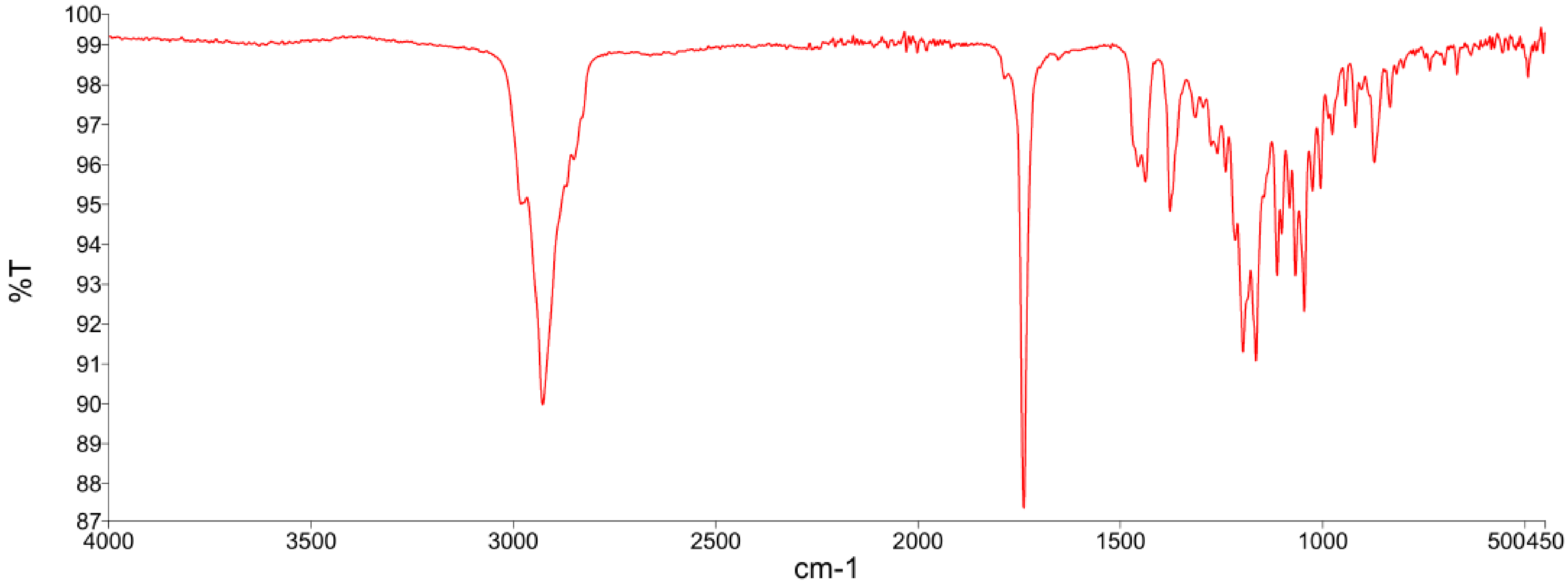

Figure S59. IR spectrum of **10**.

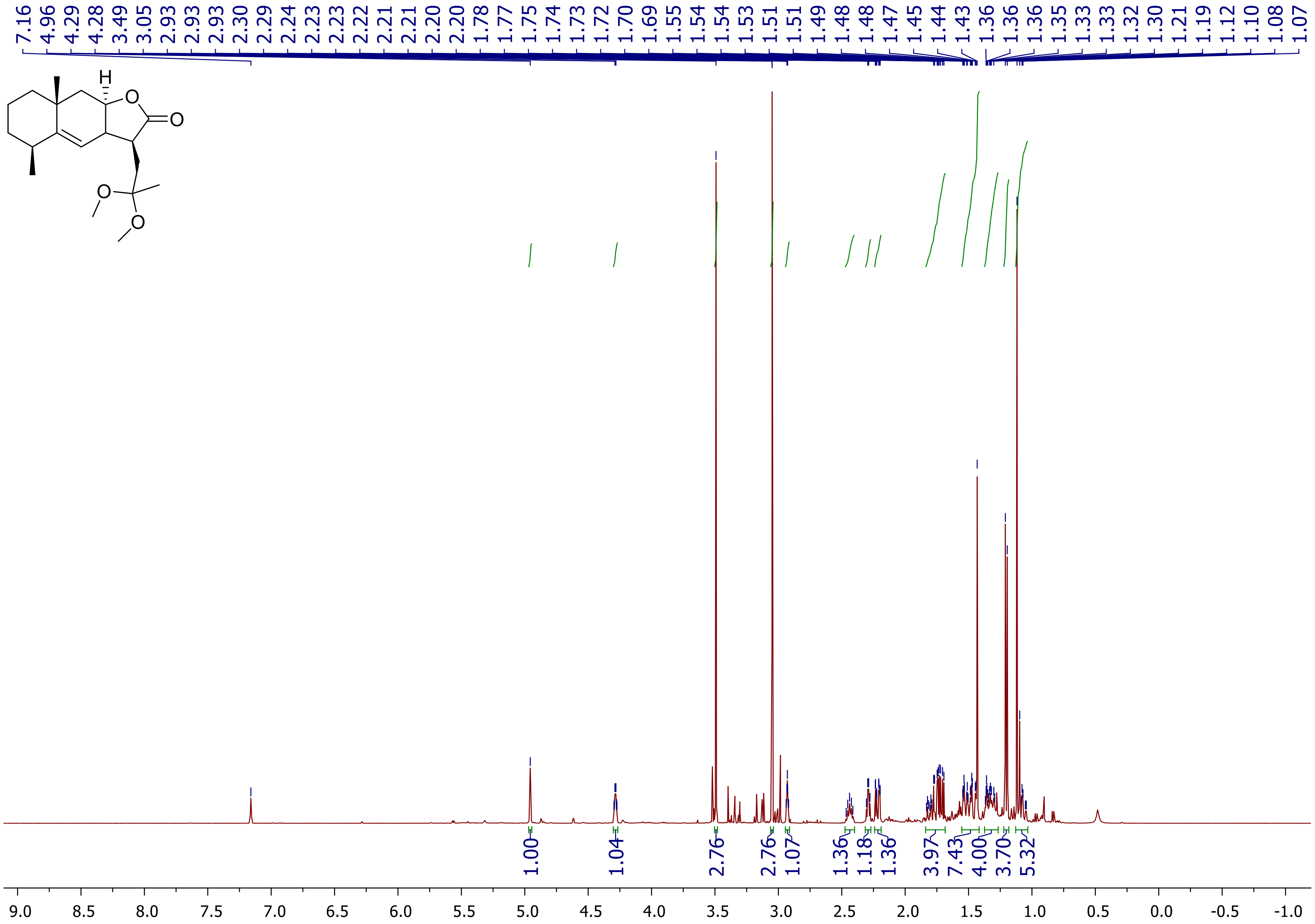

Figure S60. ^{1}H-NMR (500 MHz) spectrum of **11** in C_6D_6.

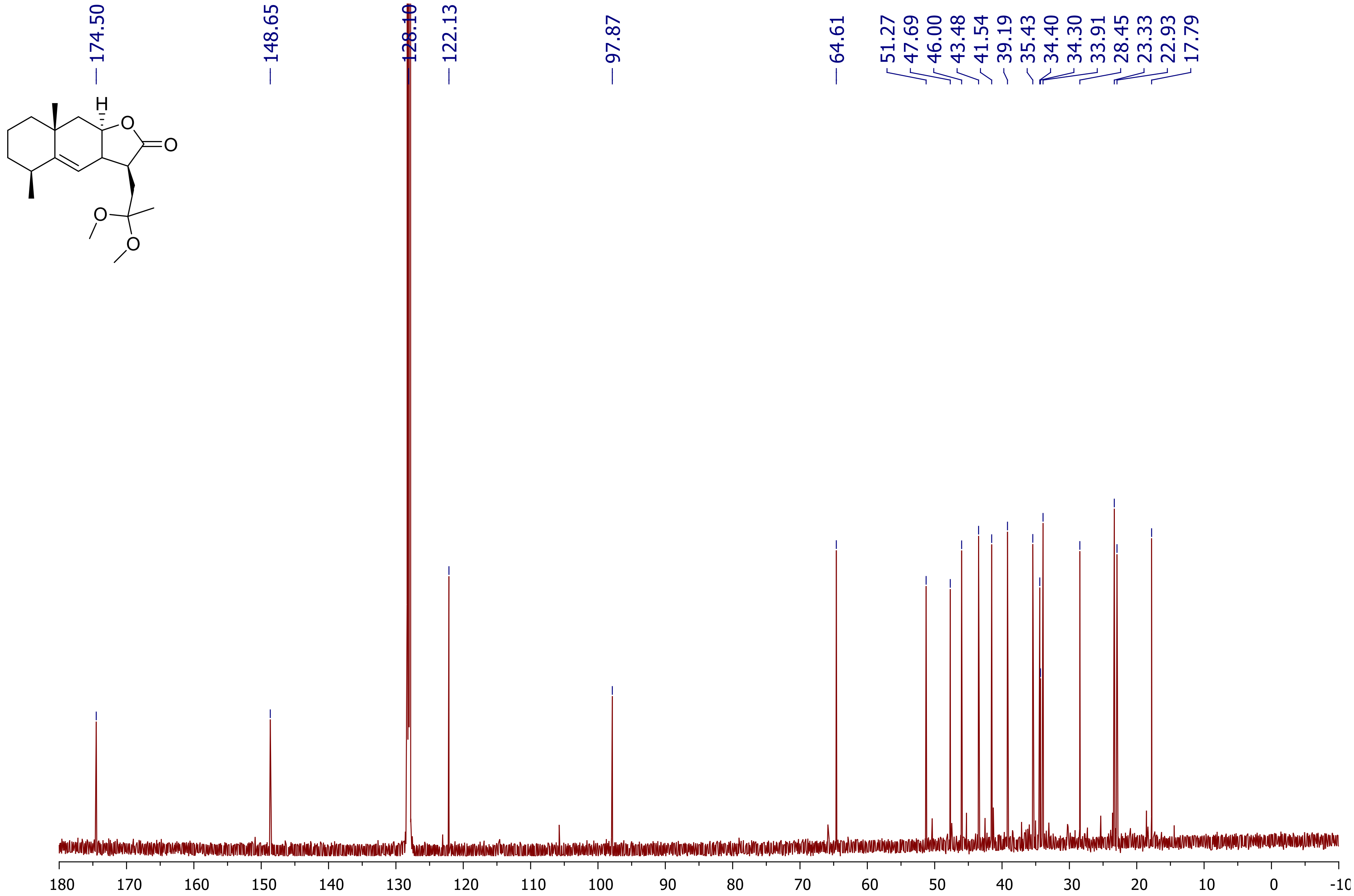

Figure S61. ^{13}C NMR (125 MHz) spectrum of **11** in C_6D_6.

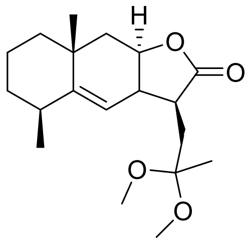

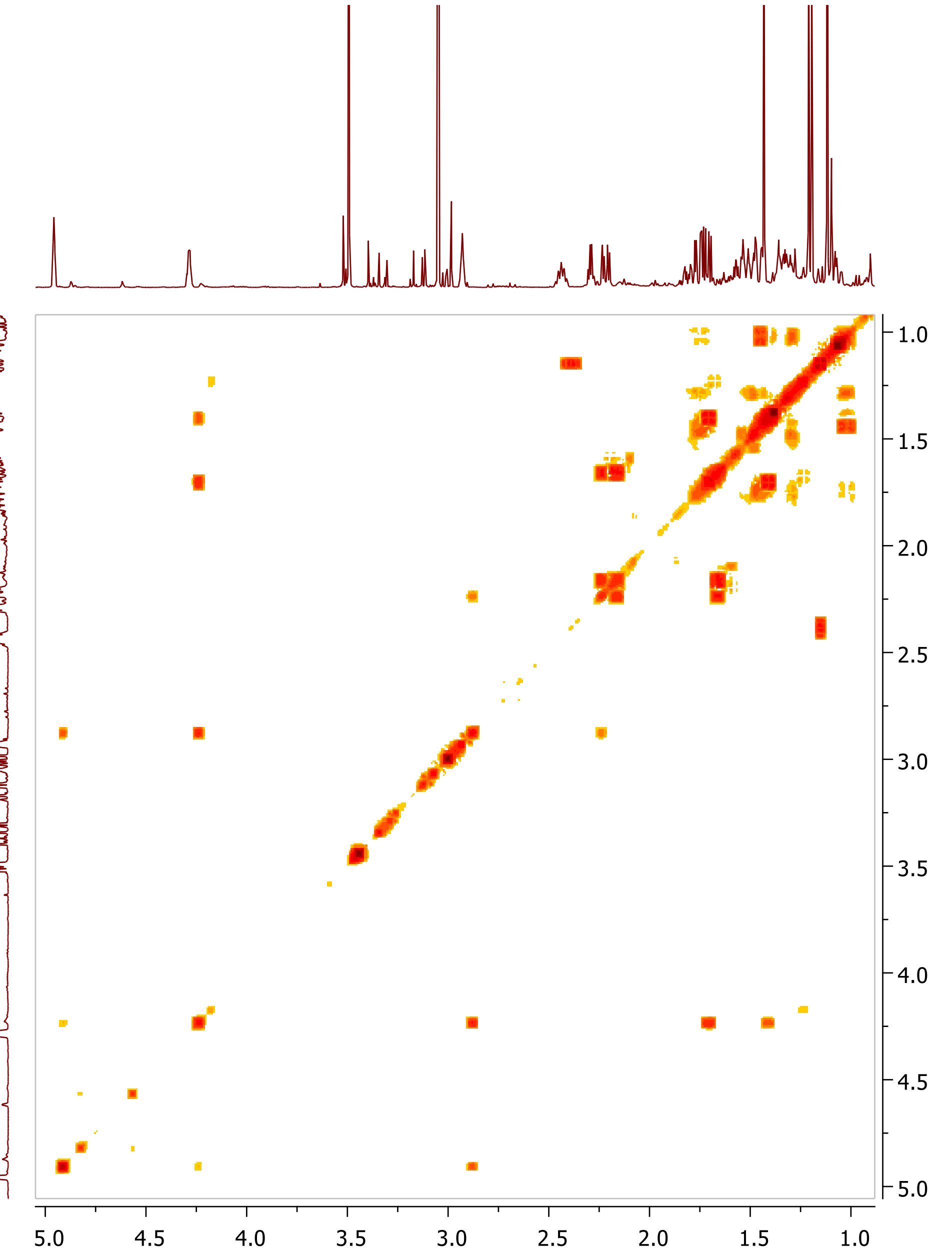

Figure S62. ^{1}H-^{1}H-COSY spectrum of **11** in C_6D_6.

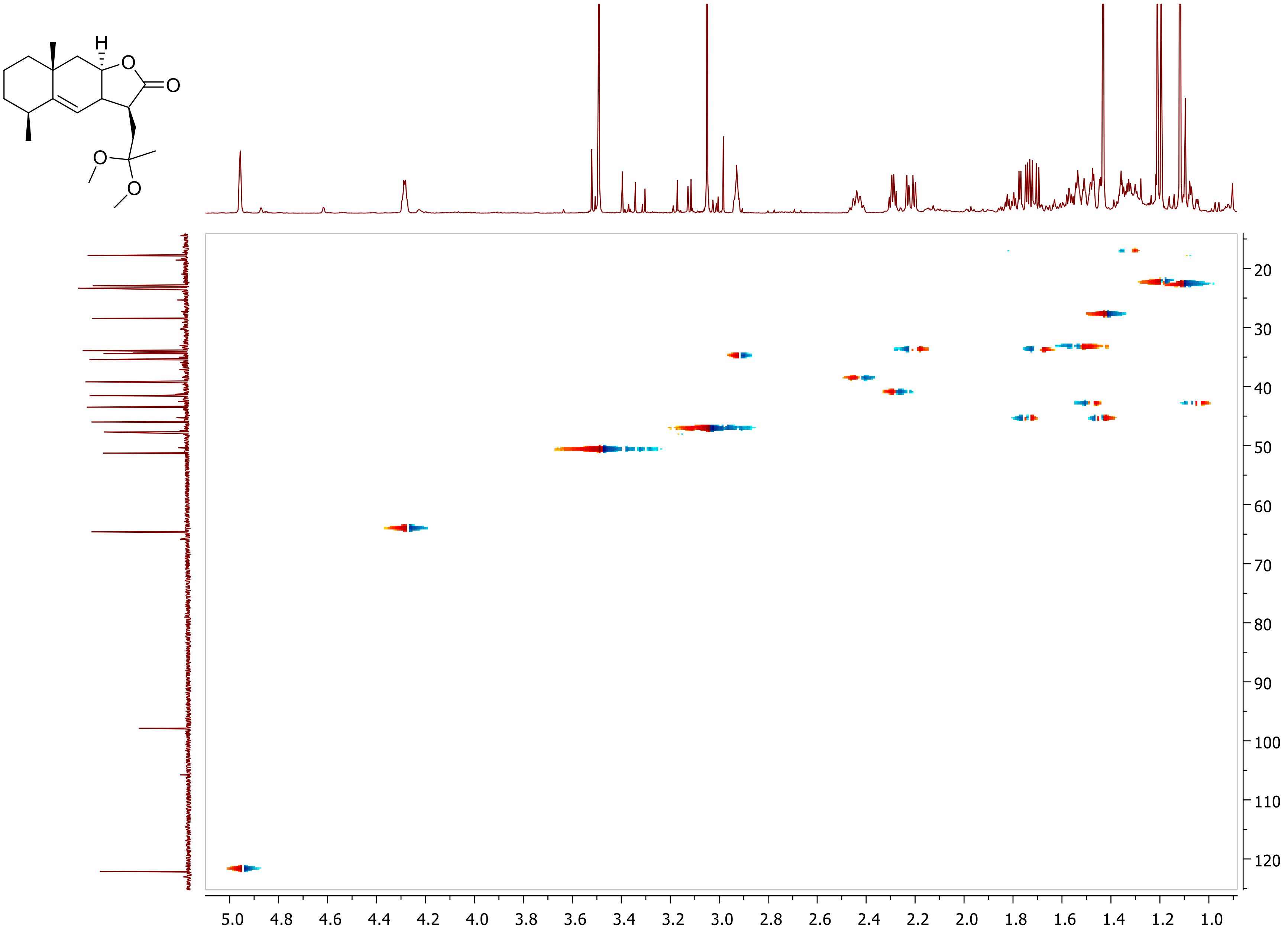

Figure S63. HSQC spectrum of **11** in C_6D_6.

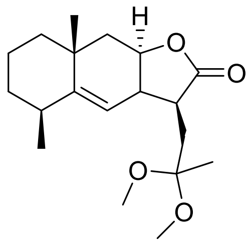

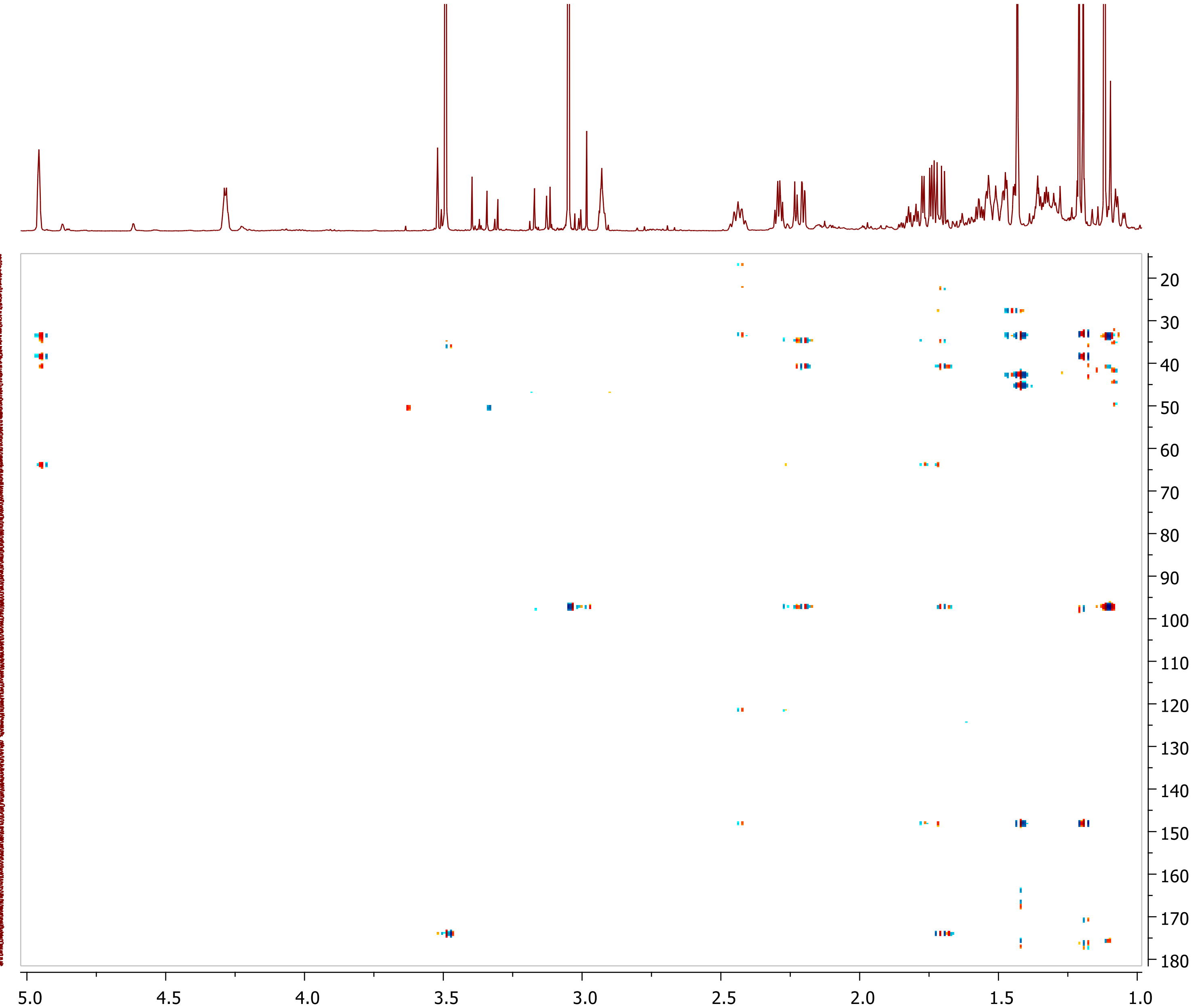

Figure S64. HMBC spectrum of **11** in C_6D_6.

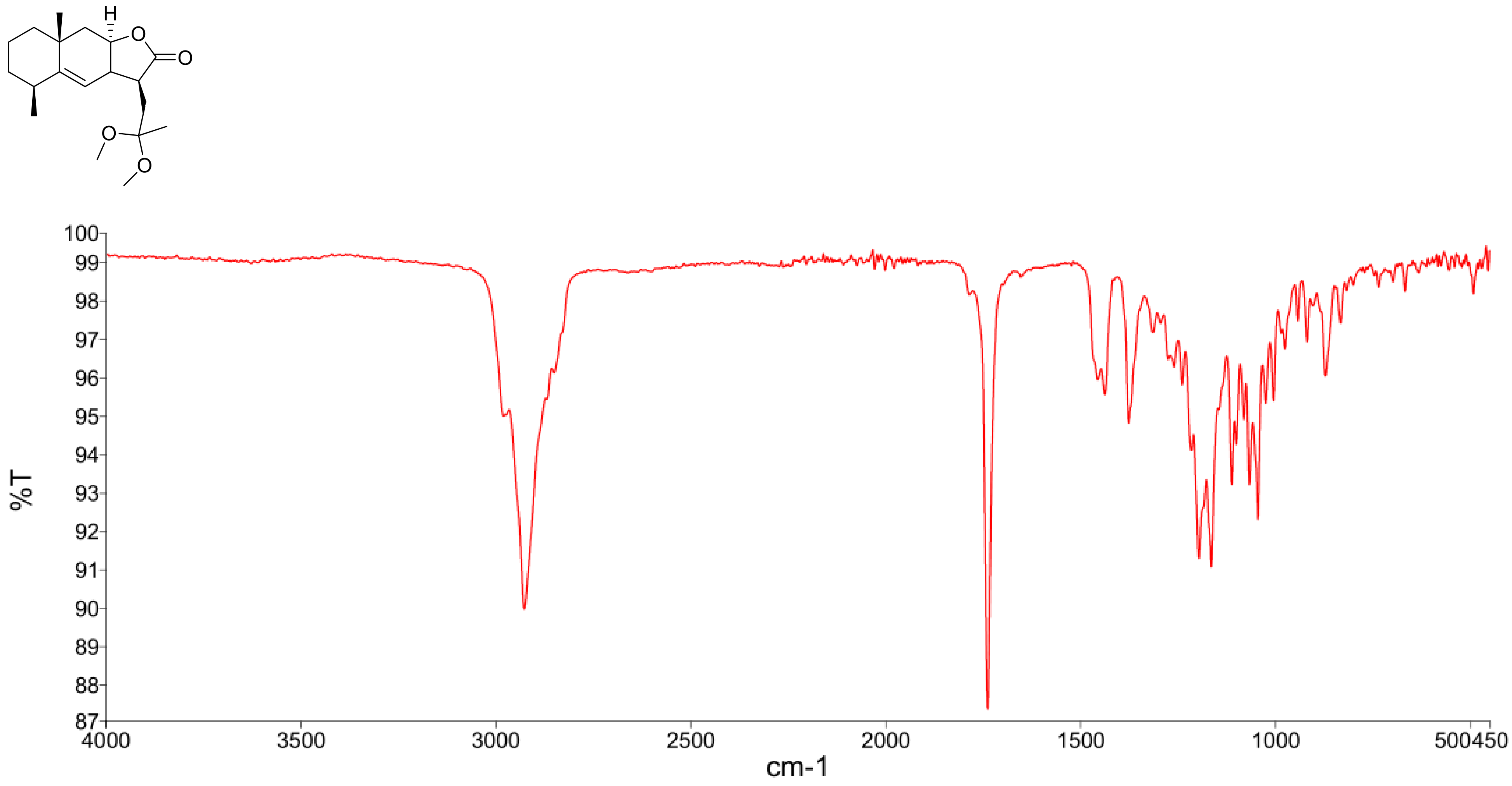

Figure S65. IR spectrum of **11**.

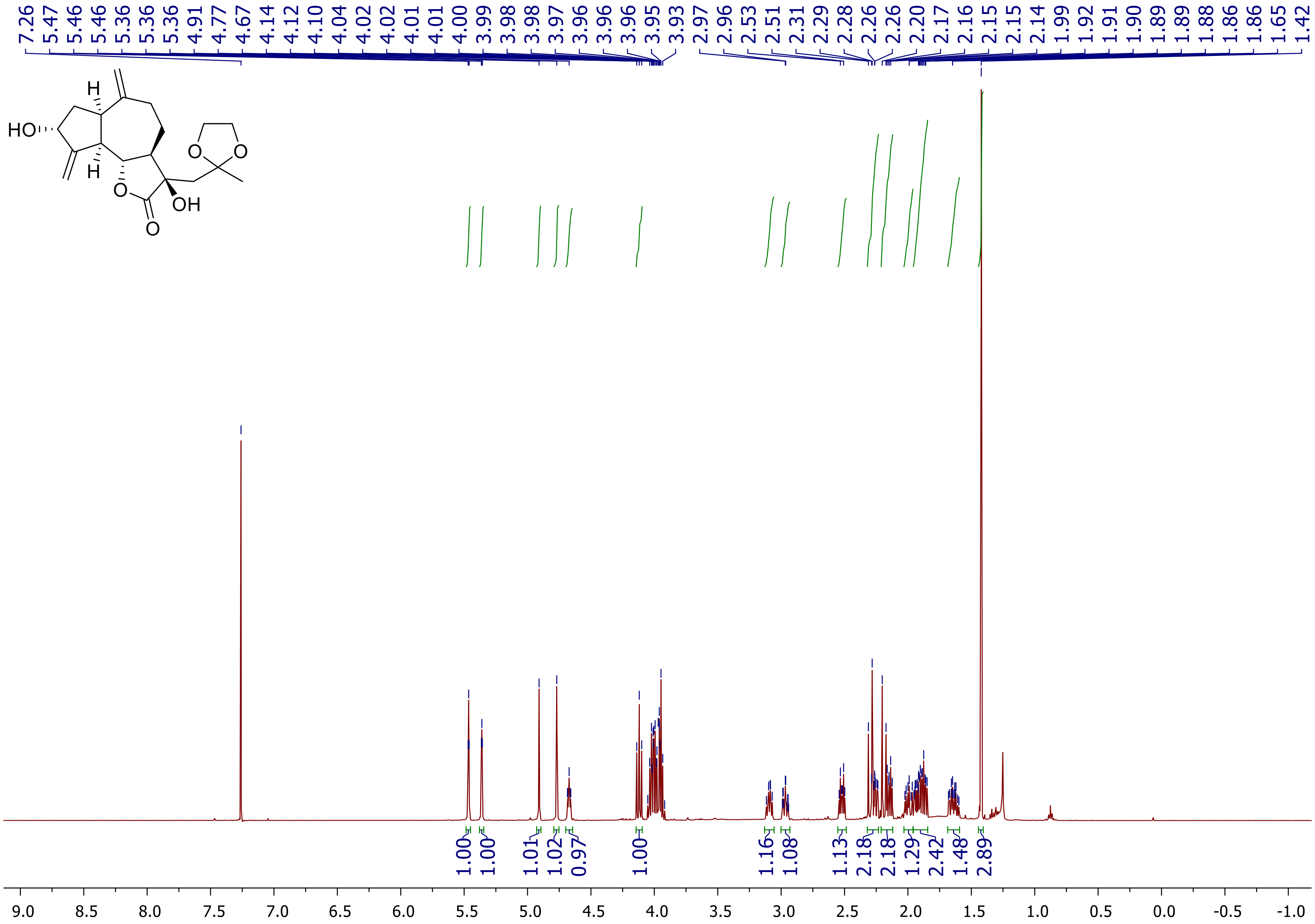

Figure S66. ^{1}H-NMR (500 MHz) spectrum of **15** in $CDCl_3$.

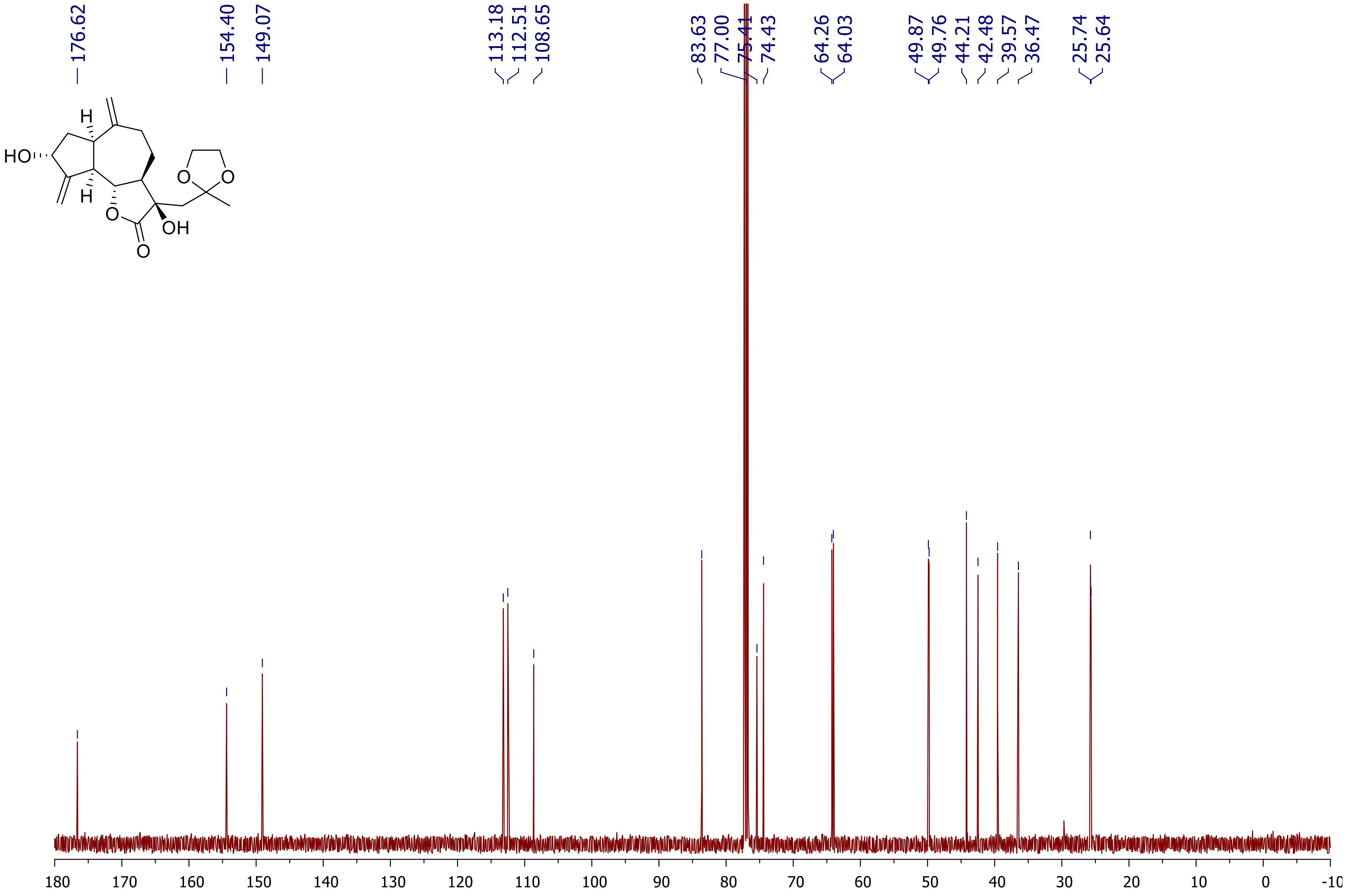

Figure S67. ^{13}C NMR (125 MHz) spectrum of **15** in $CDCl_3$.

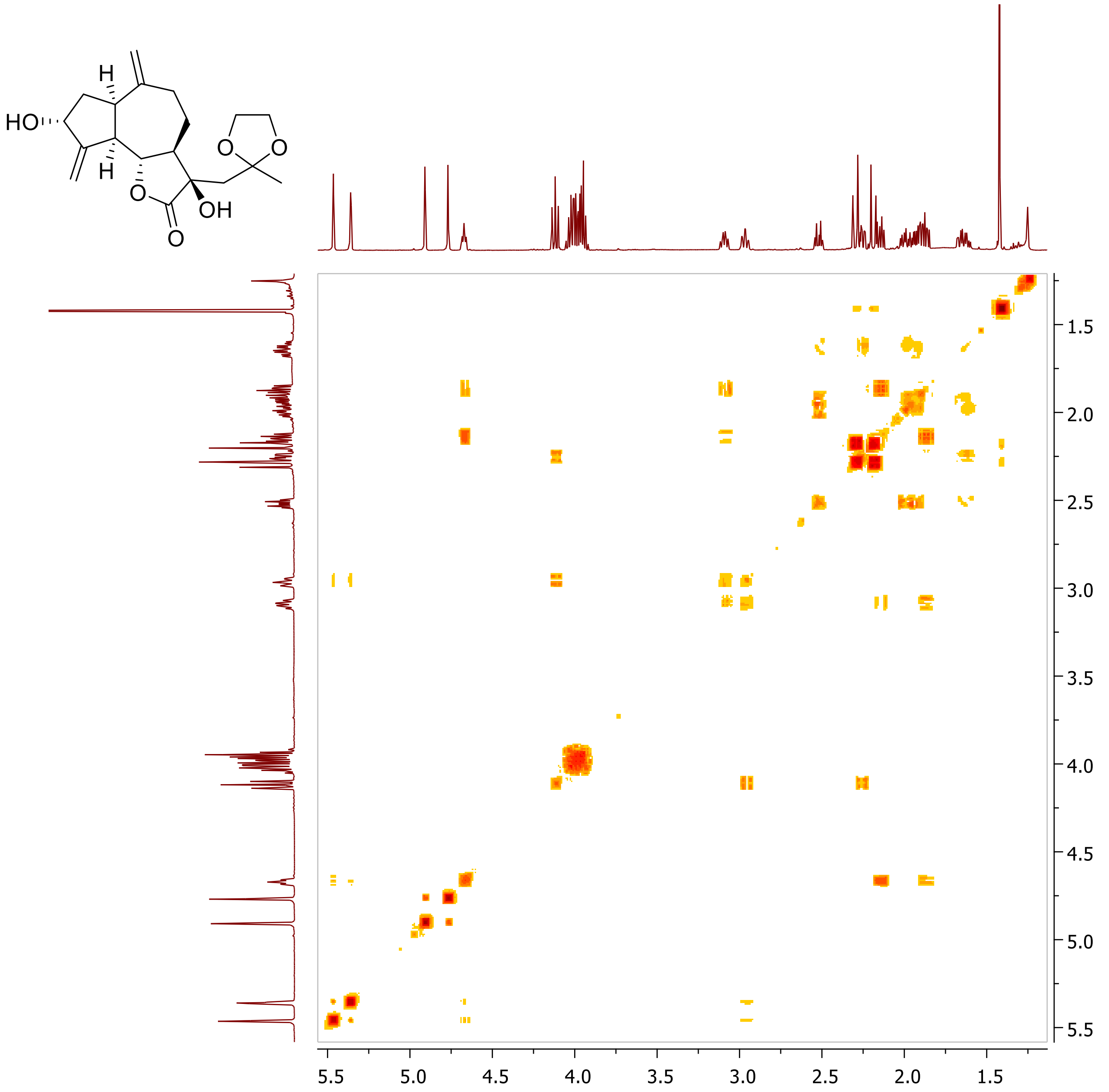

Figure S68. ^{1}H-^{1}H-COSY spectrum of **15** in $CDCl_3$.

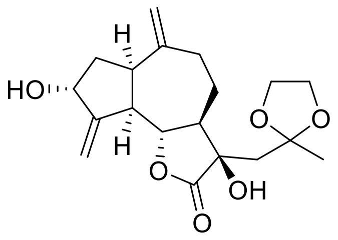

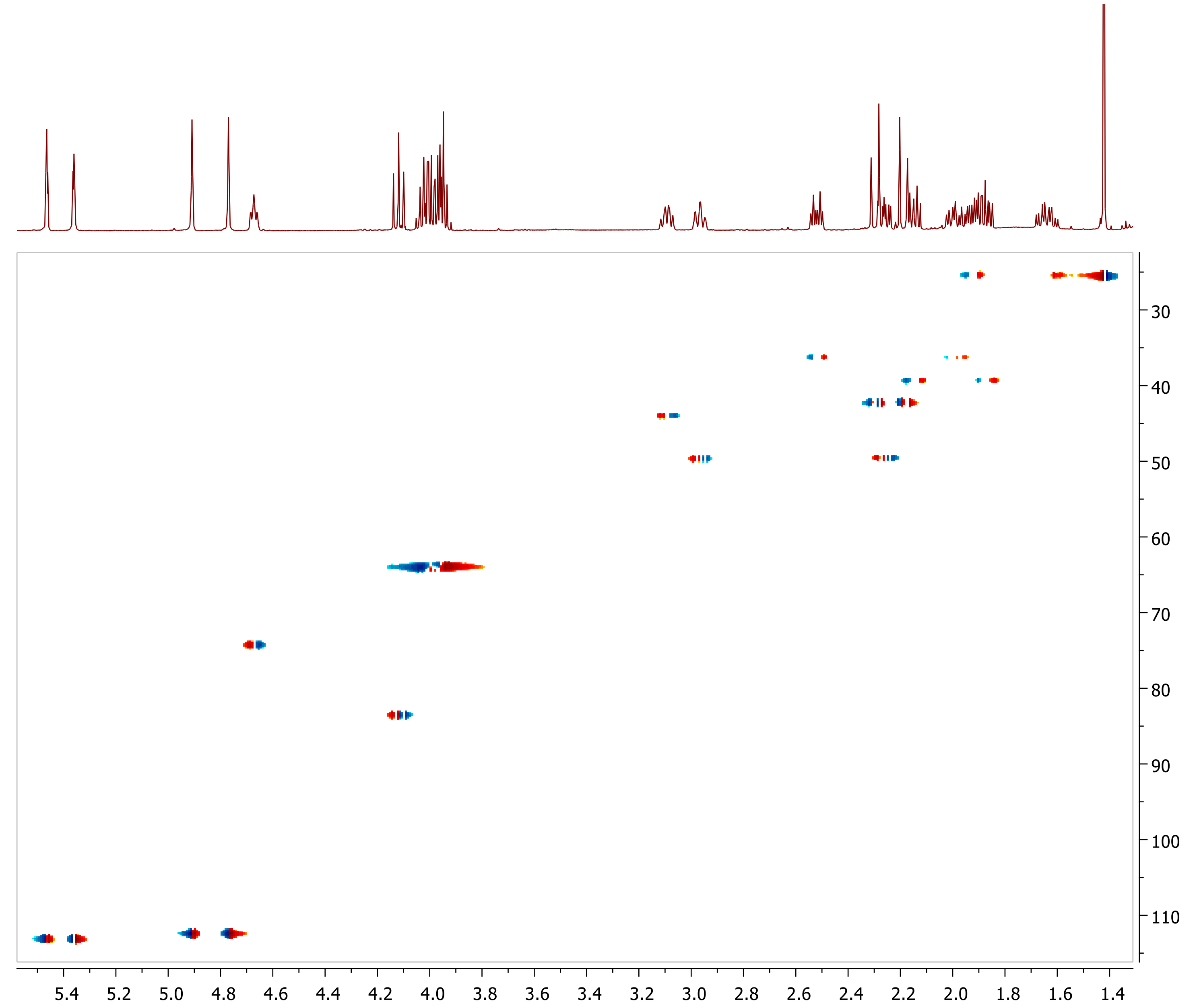

Figure S69. HSQC spectrum of **15** in $CDCl_3$.

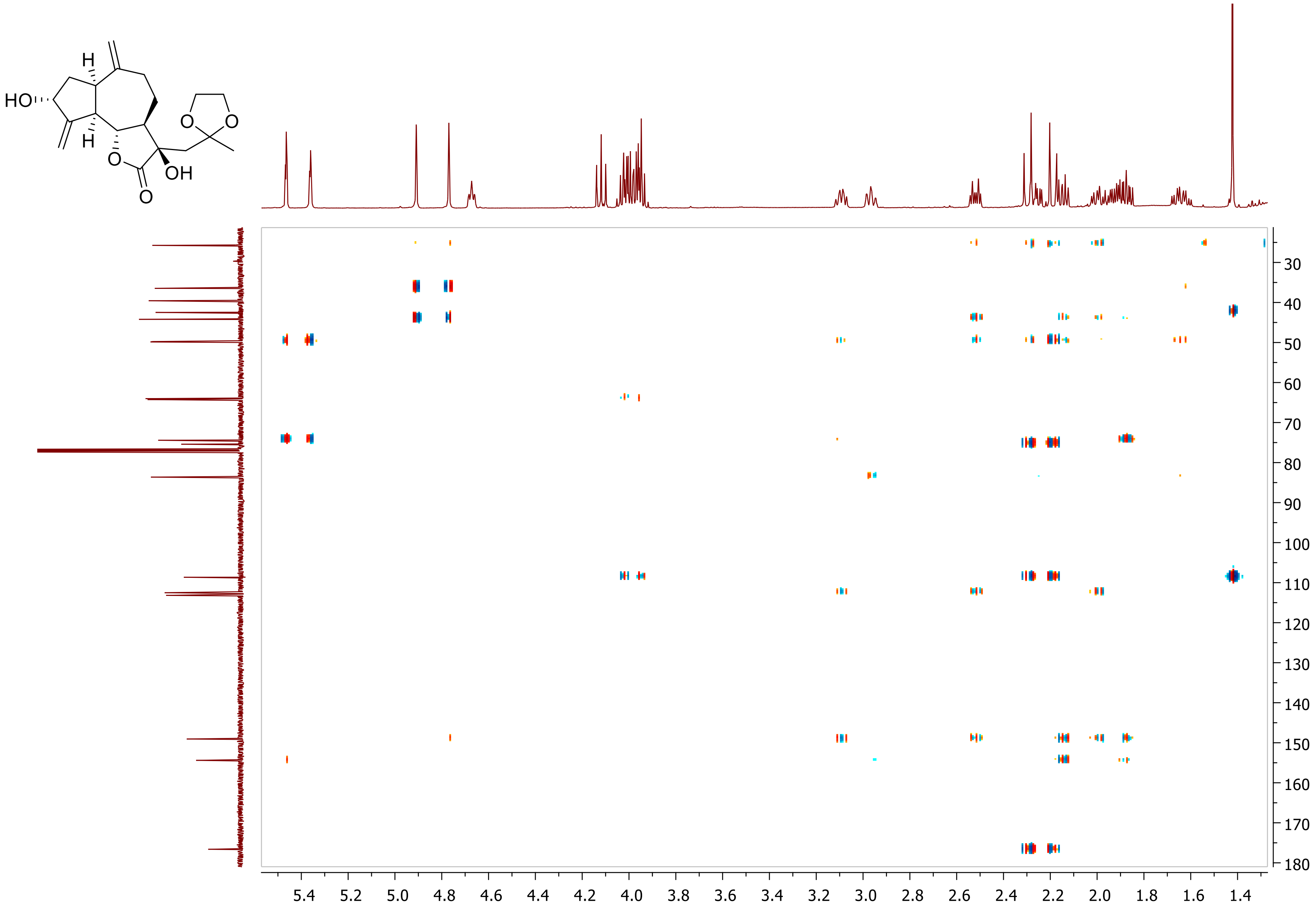

Figure S70. HMBC spectrum of **15** in $CDCl_3$.

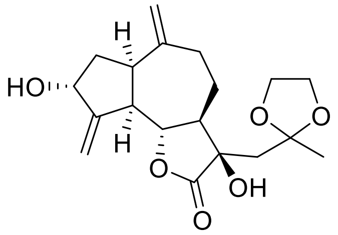

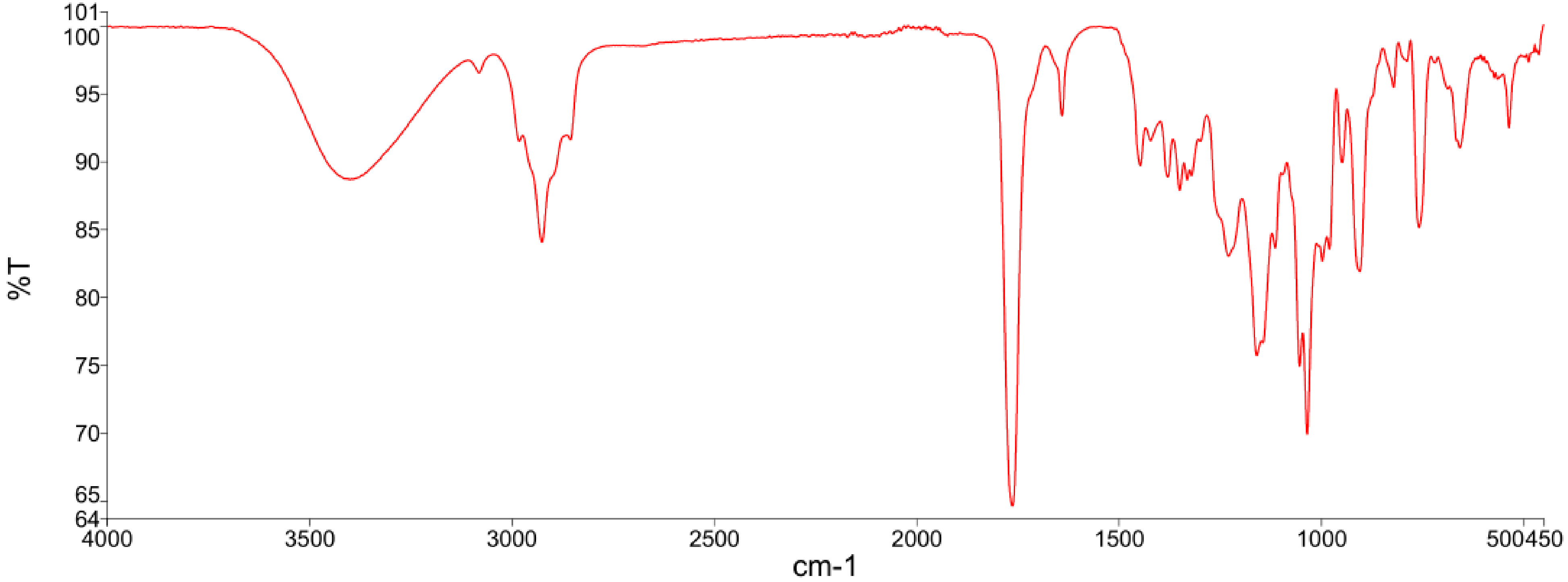

Figure S71. IR spectrum of **15**.

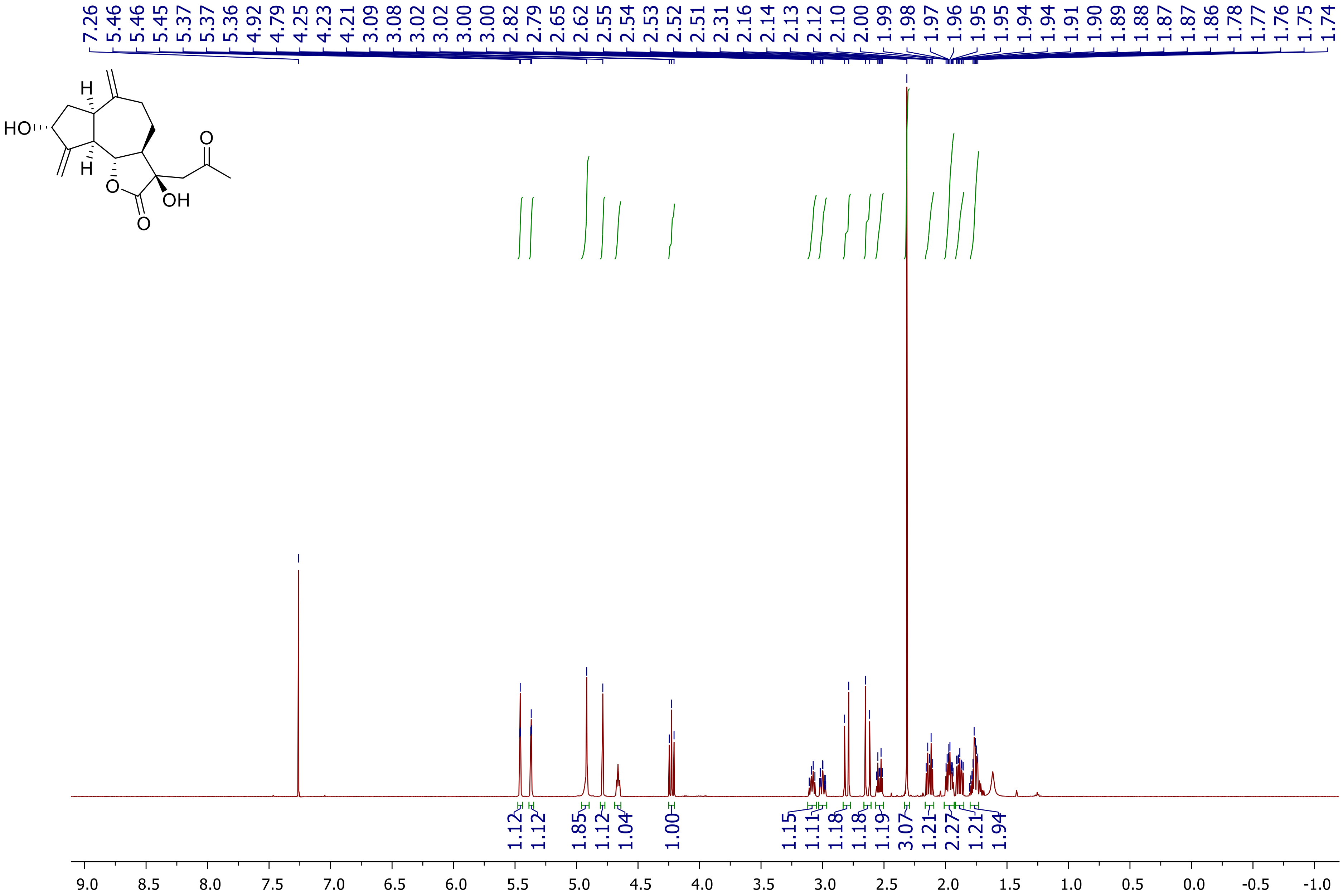

Figure S72. ^{1}H-NMR (500 MHz) spectrum of **16** in $CDCl_3$.

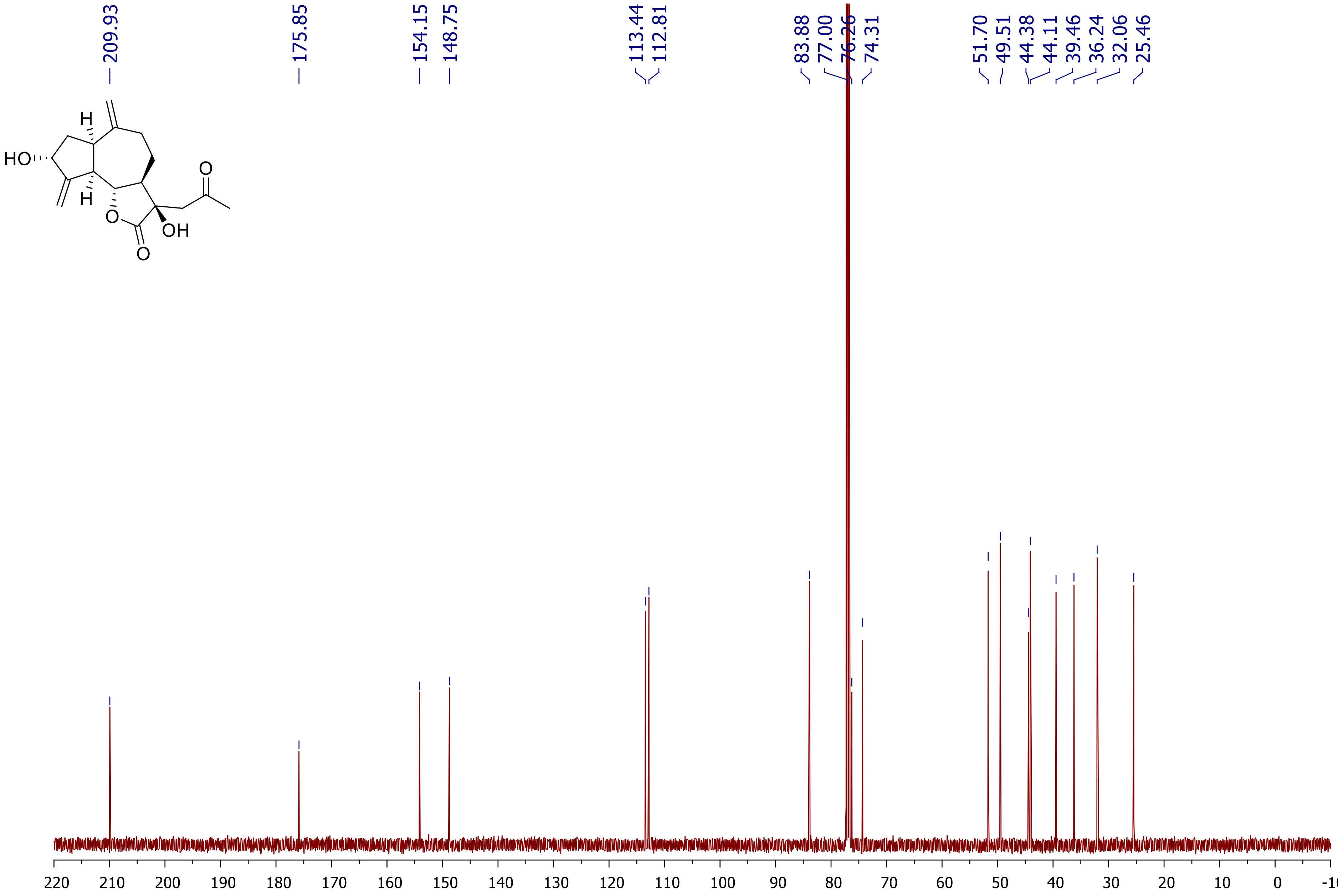

Figure S73. ^{13}C NMR (125 MHz) spectrum of **16** in $CDCl_3$.

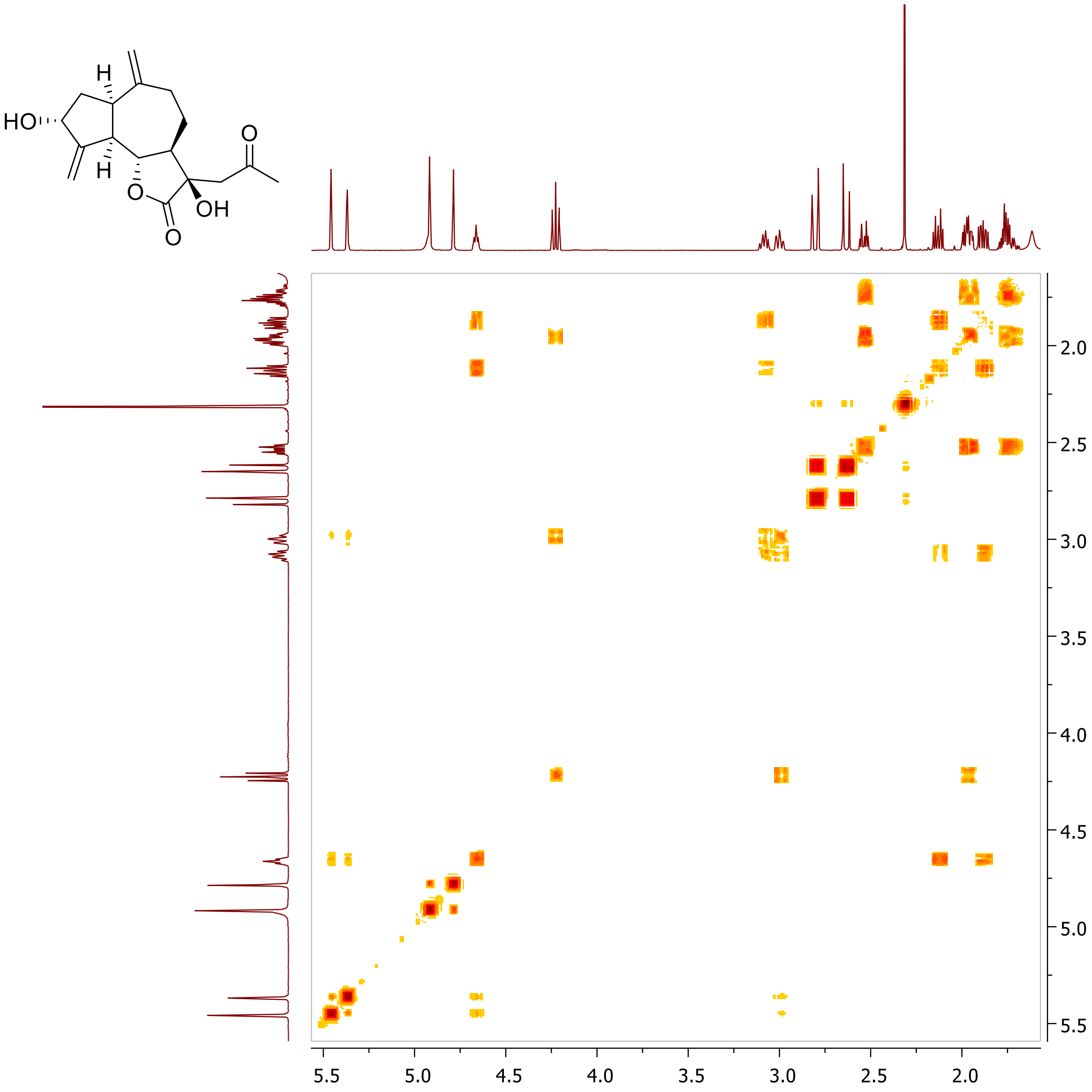

Figure S74. ^{1}H-^{1}H-COSY spectrum of **16** in $CDCl_3$.

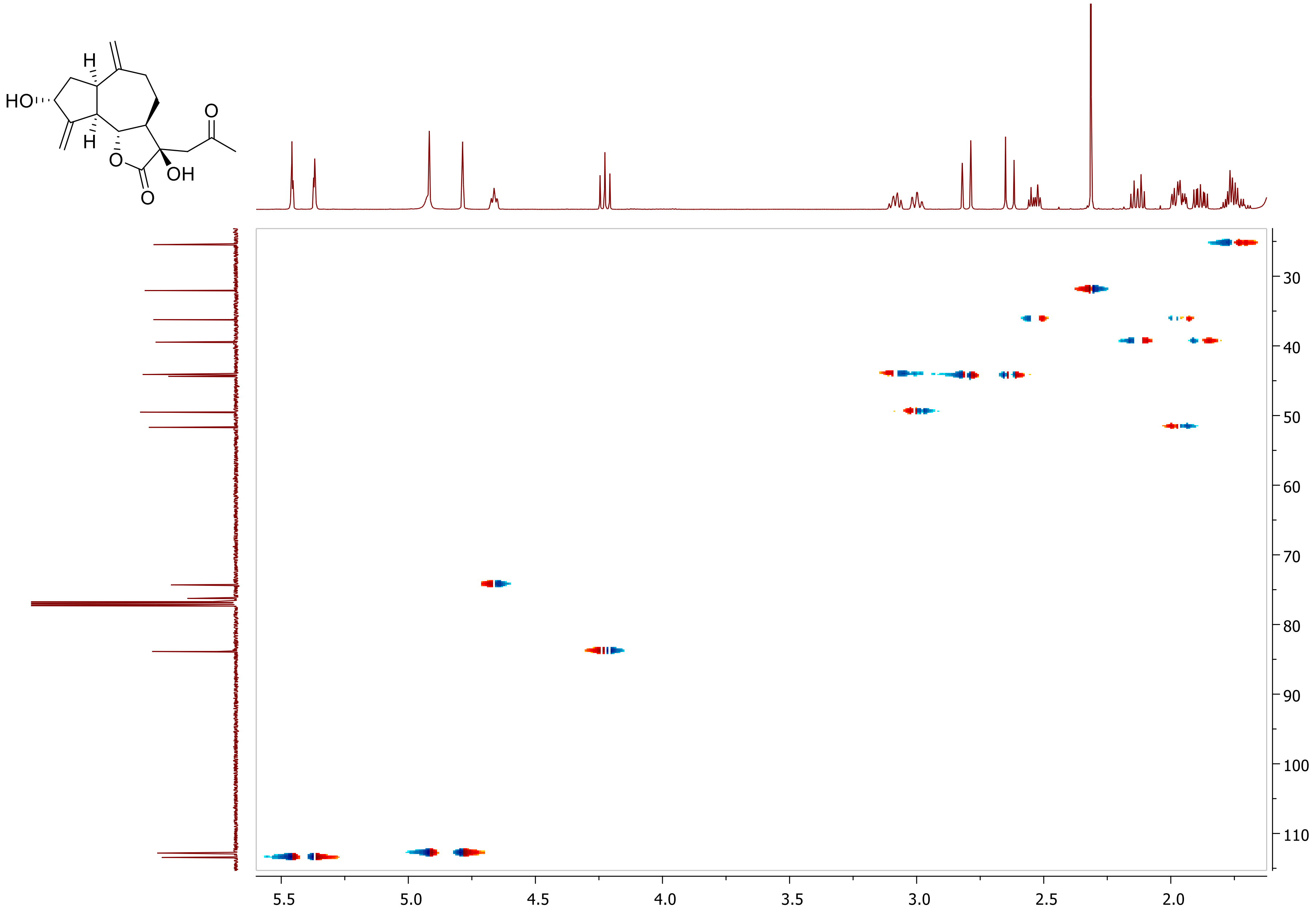

Figure S75. HSQC spectrum of **16** in $CDCl_3$.

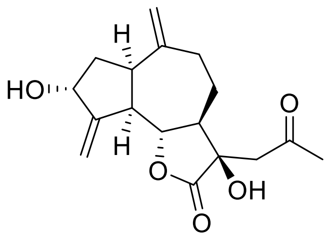

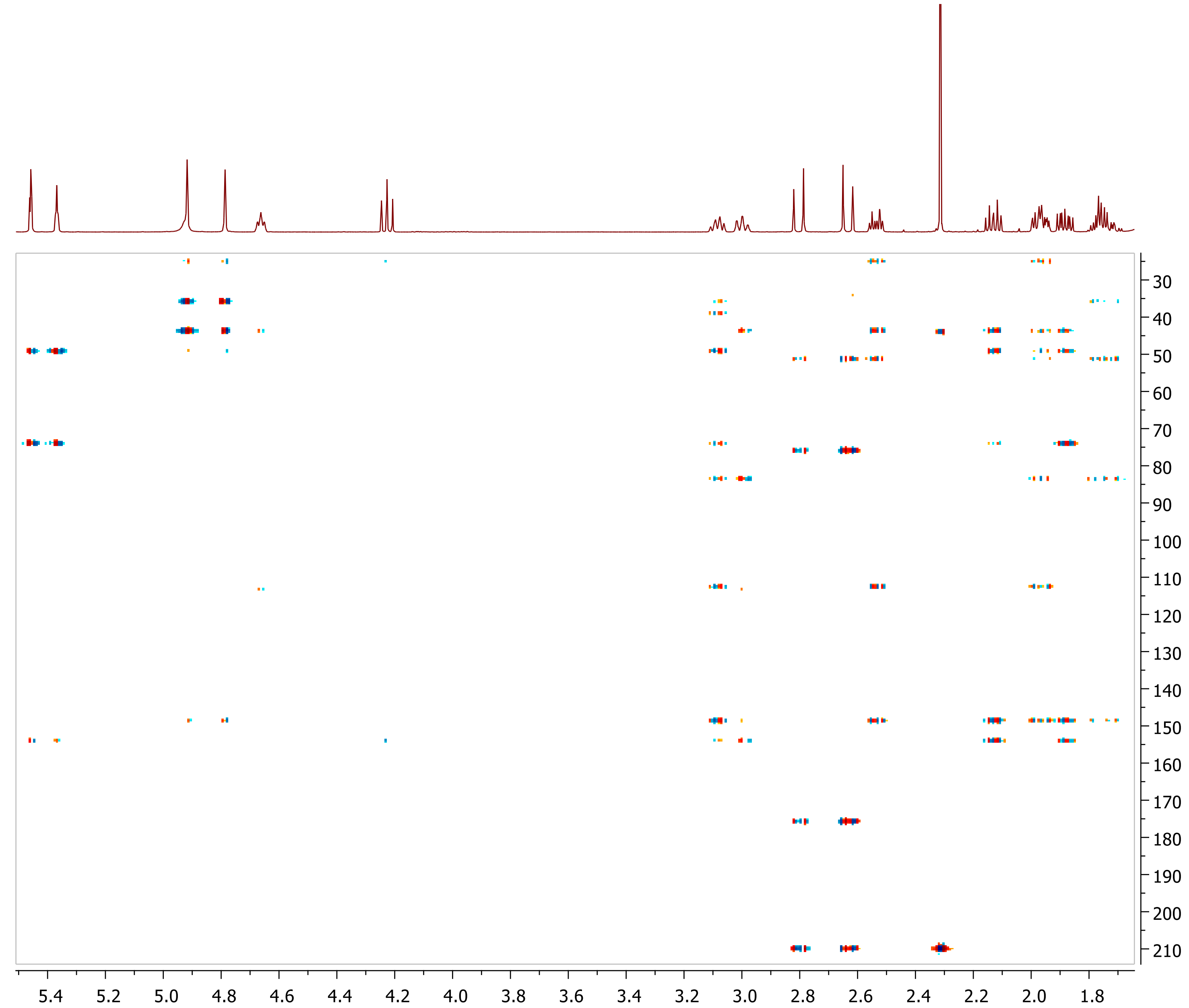

Figure S76. HMBC spectrum of **16** in $CDCl_3$.

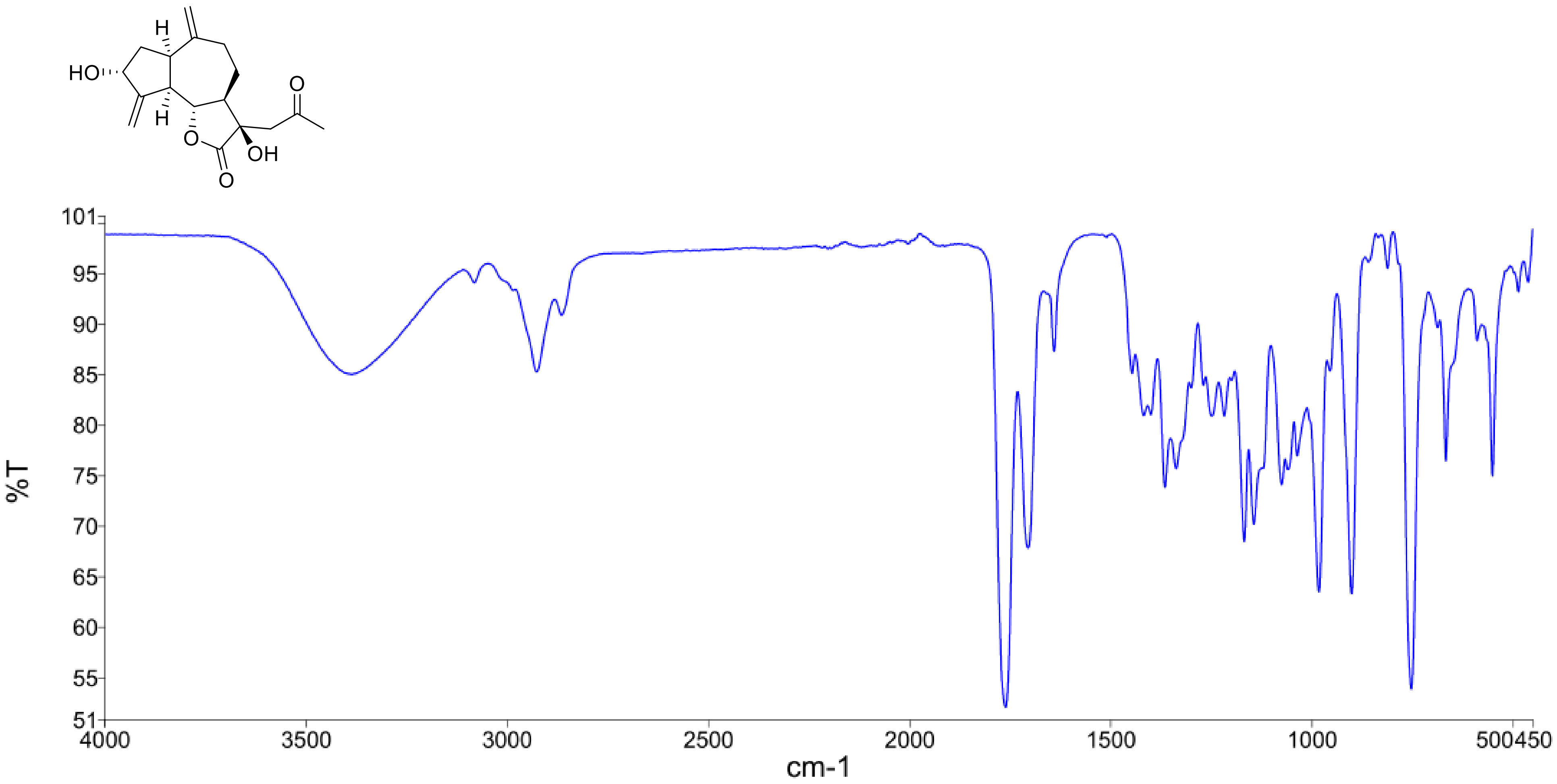

Figure S77. IR spectrum of **16**.
